# Supplementary material for: Long-range enhancer-controlled genes are hypersensitive to regulatory factor perturbations
Source: Cell Genom. 2025 Feb 25;5(3):100778. doi: 10.1016/j.xgen.2025.100778 (PMC11960515; doi:10.1016/j.xgen.2025.100778)
Supplement: Document S1. Figures S1–S9 [file mmc1.pdf]

Cell Genomics, Volume *5*

Supplemental information

Long-range enhancer-controlled genes are hypersensitive to regulatory factor perturbations

Sjoerd J.D. Tjalsma, Niels J. Rinzema, Marjon J.A.M. Verstegen, Michelle J. Robers, Andrea Nieto-Aliseda, Richard A. Gremmen, Amin Allahyar, Mauro J. Muraro, Peter H.L. Krijger, and Wouter de Laat

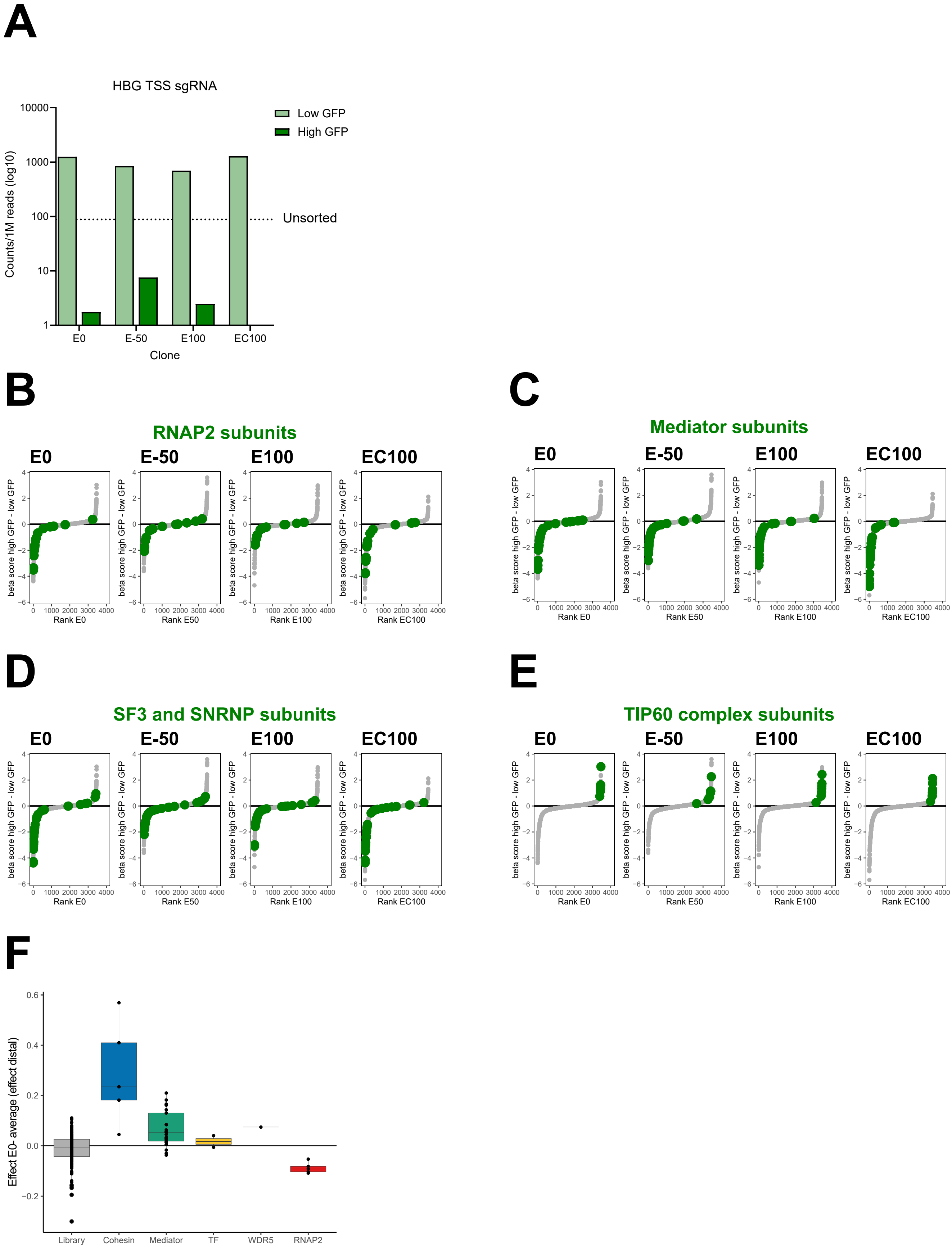

Figure S1. Reporter CRISPRi screen, related to Figure 1. (A) Read count of sgRNA directly targeting the HBG-reporter promoter, in the low GFP (light green) or high GFP (dark green) conditions, normalized for sequencing depth. The dotted line indicates the counts in the library isolated for unsorted cells. **(B-E)** Distribution of subunits of RNAP2 **(B)**, mediator **(C)**, the SF3 or SNRNP complexes **(D)**, and the TIP60 complex **(E)** in the CRISPRi screens per reporter cell line. (**F)** Box plots comparing the average distal enrichment score with the E0 score for factors affecting distal reporter expression. Factors with a normalized score of ≤ -0.1 in at least one of the distal gates were compared to E0 and ranked on the difference with the average of the three distal reporters. Each complex highlighted in figure 1 is plotted as a separate box plot.

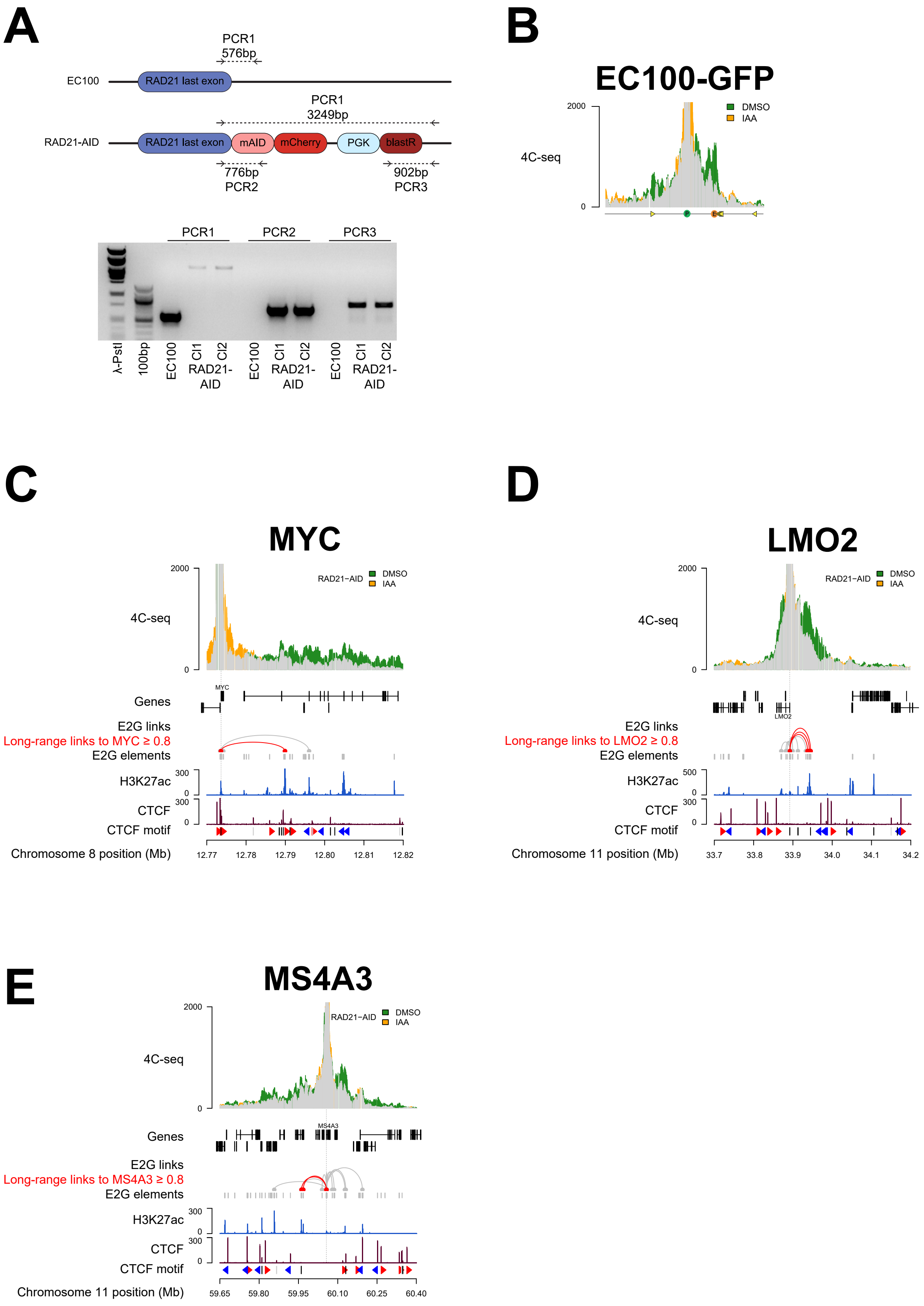

Figure S2. Generation and validation of RAD21-AID cell lines, related to Figure 2. (A) Genotyping PCRs of RAD21-AID clones. EC100 = parental cell line (untargeted). **(B-E)** 4C-seq profiles of EC100-GFP reporter cells, treated for 2 hours with DMSO or 5-Ph-IAA (IAA). The average profile of two clones is plotted as overlay of DMSO vs IAA. In green, interactions specific for DMSO samples, in yellow interactions specific for IAA samples, in grey shared interactions. Viewpoints: **(B)** EC100-GFP reporter locus. **(C)** MYC. **(D)** LMO2. **(E)** MS4A3. For **(B)** yellow triangles denote CTCF sites. E: enhancer. P: reporter promoter.

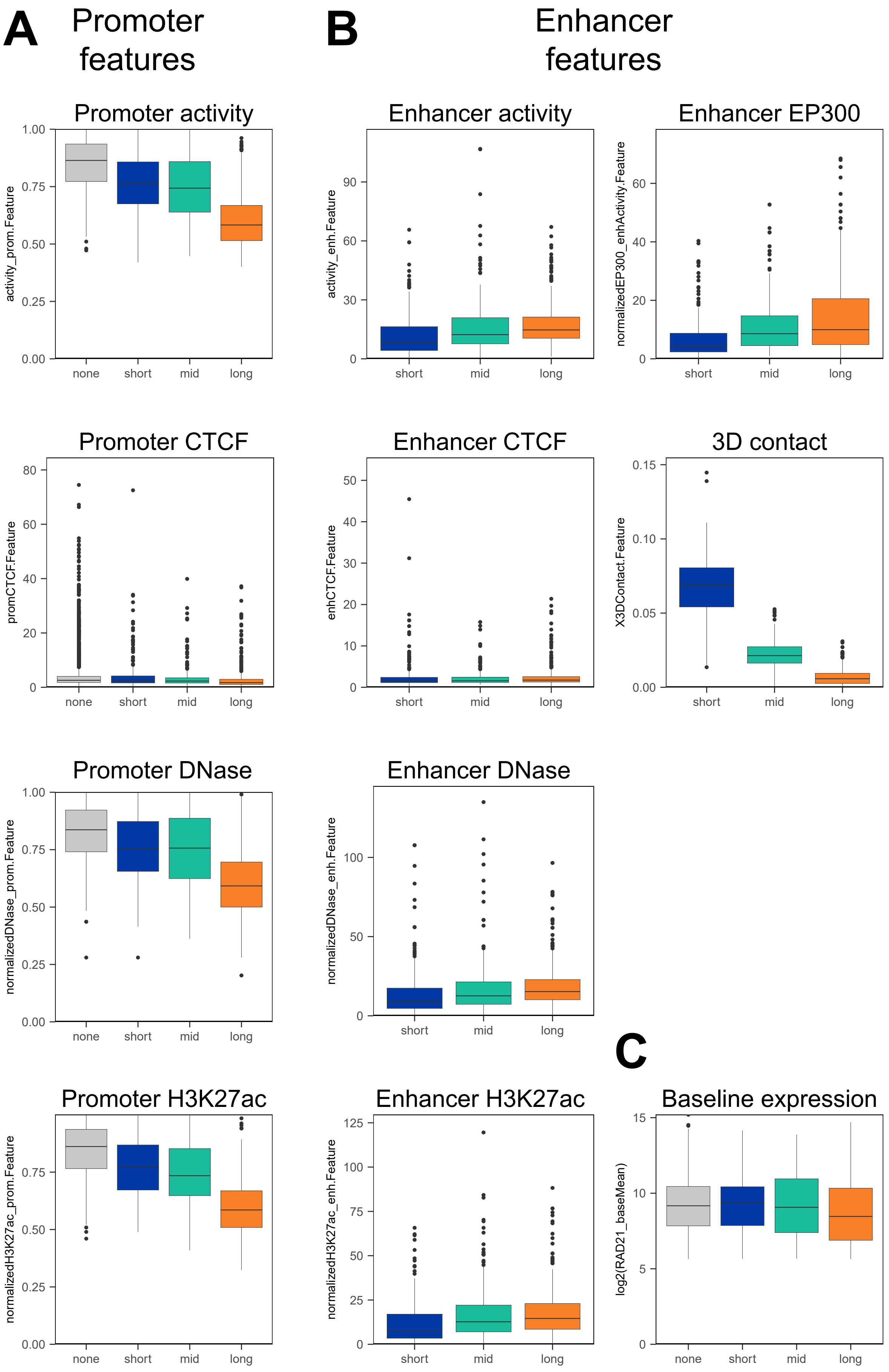

Figure S3. Characterization of enhancer-contacting genes, related to Figure 2. Features of promoters **(A)** and enhancers **(B)** in the ENCODE-rE2G datasets. For genes that contacted multiple enhancers within the same group (eg. 2 long-range enhancers both with an ABC score ≥ 0.8), the enhancer with the highest ABC score was picked for these analyses. **(C)** Baseline counts of enhancer categorized genes (baseMean counts from DEseq2).

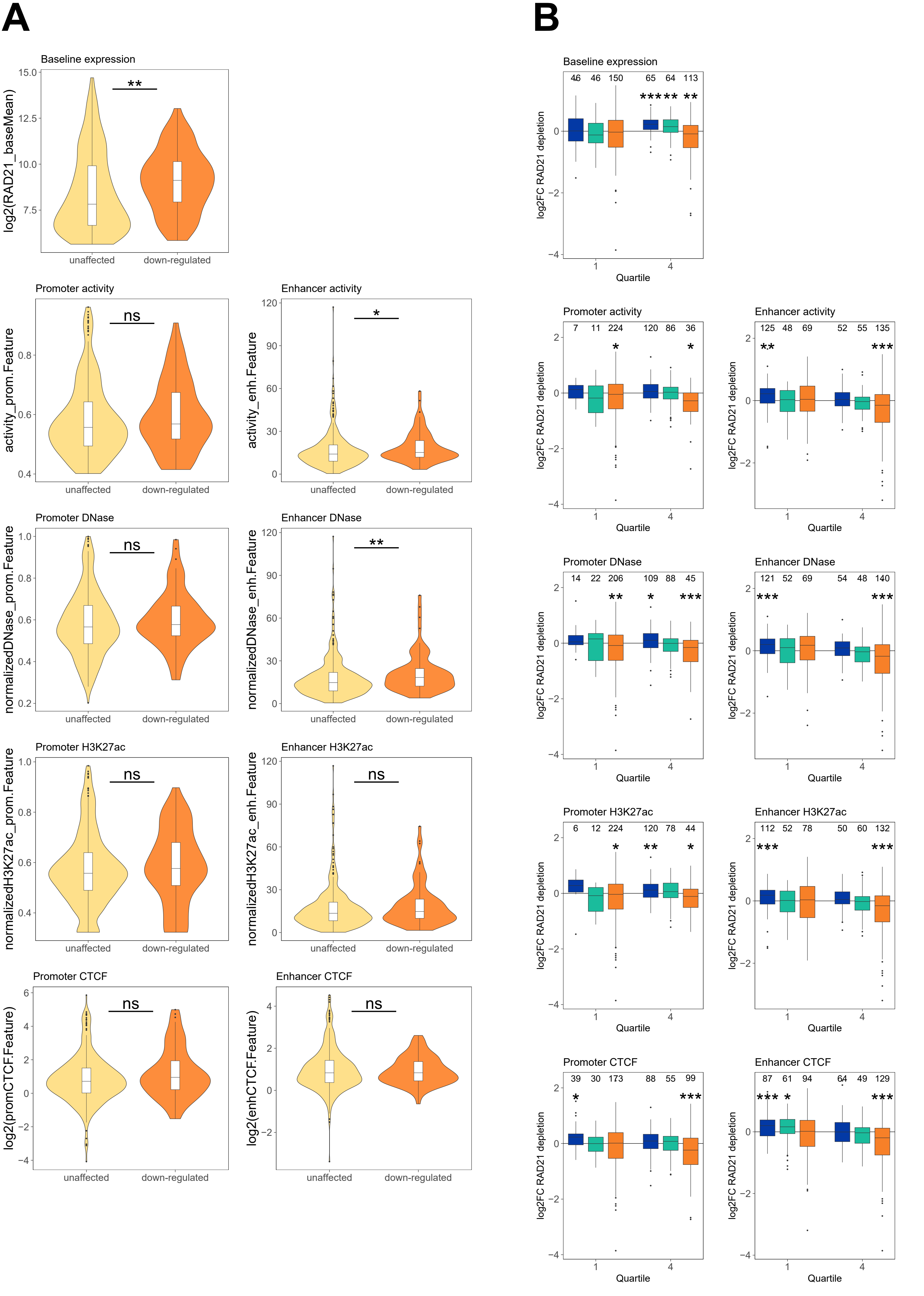
A
B
Baseline expression
log2(RAD21_baseMean)
15.0
12.5
10.0
7.5
**
unaffected
down-regulated
Promoter activity
activity_prom.Feature
ns
Enhancer activity
activity_enh.Feature
*
Promoter DNase
normalizedDNase_prom.Feature
ns
Enhancer DNase
normalizedDNase_enh.Feature
**
Promoter H3K27ac
normalizedH3K27ac_prom.Feature
ns
Enhancer H3K27ac
normalizedH3K27ac_enh.Feature
ns
Promoter CTCF
log2(promCTCF.Feature)
ns
Enhancer CTCF
log2(enhCTCF.Feature)
ns
log2FC RAD21 depletion
Quartile
1
4
46 46 150 65 64 113
7 11 224 120 86 36
125 48 69 52 55 135
14 22 206 109 88 45
121 52 69 54 48 140
6 12 224 120 78 44
112 52 78 50 60 132
39 30 173 88 55 99
87 61 94 64 49 129

Figure S4. Features of RAD21 responsive genes, related to Figure 2. (A) Comparison of enhancer features of long-range enhancers found down-regulated (log2FC < -0.8, FDR < 0.05) or unaffected (-0.2 < log2FC < 0.2) by RAD21 depletion. n=332 for unaffected, n= 72 for down-regulated. **(B)** Comparison of response to RAD21 depletion for genes connected to enhancers with specific features. For every feature, the strongest enhancer per gene in the short (blue), mid (green), and long orange) categories were pooled and ranked on the feature. Then, the lowest 25% (quartile 1) and the highest 25% (quartile 4) of these enhancers were selected, and the response of their connected genes to RAD21 depletion was depicted as log2FC. p-values calculated with 2-sided Wilcoxon rank-sum test of the specific group versus the no enhancer genes. ns=non-significant, *=p<0.05, **=p<0.01, ***=p<0.001.

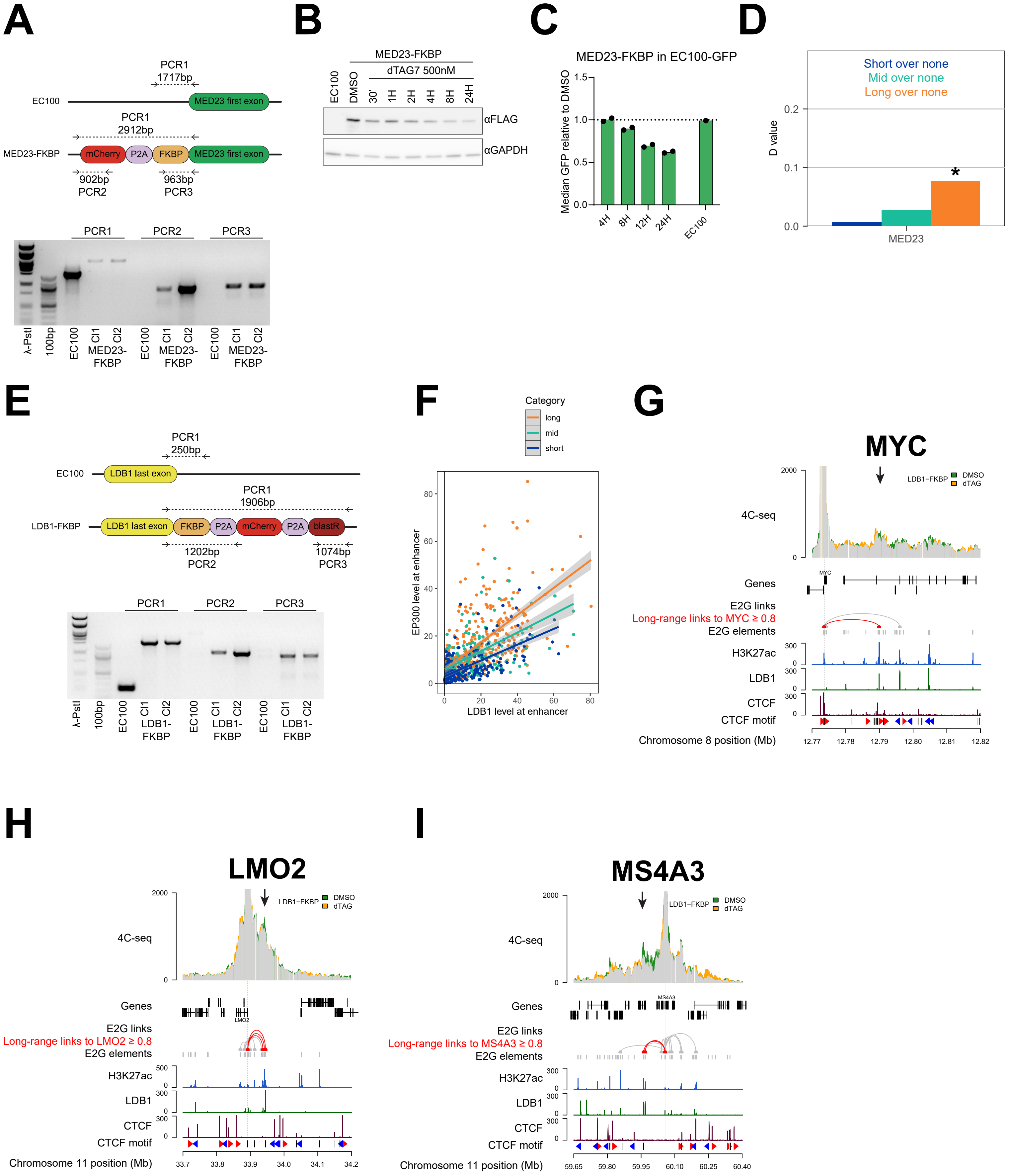

Figure S5. Generation and validation of MED23 and LDB1 degron cell lines, related to Figure 3. (A) Genotyping PCRs of FKBP-MED23 clonal cell lines. **(B)** Western blot for FLAG-tagged FKBP-MED23, fter treatment with 500nM dTAG7 for indicated time points, one representative FKBP-MED23 clone. **(C)** FACS validation of GFP reporter after MED23 depletion. Each dot represents one FKBP-MED23 clone. **(D)** D values of one-sided Komolgorov-Smirnov test, for three enhancer categories over no enhancer genes, *=p<0.05. n=2 cell lines. **(E)** Genotyping PCRs of LDB1-FKBP clonal cell lines. **(F)** Scatter plot comparing linked E2G enhancers in EP300 level (y-axis) versus LDB1 level (x-axis). **(G-I)** 4C-seq profiles of LDB1-FKBP cells, treated for 4 hours with DMSO or dTAG7 (dTAG). The average profile of two clones is plotted as overlay of DMSO vs dTAG. In green, interactions specific for DMSO samples, in yellow interactions specific for dTAG samples, in grey shared interactions. Viewpoints: **(G)** MYC, **(H)** LMO2, **(I)** MS4A3. Arrow denotes strong enhancer with LDB1 binding.

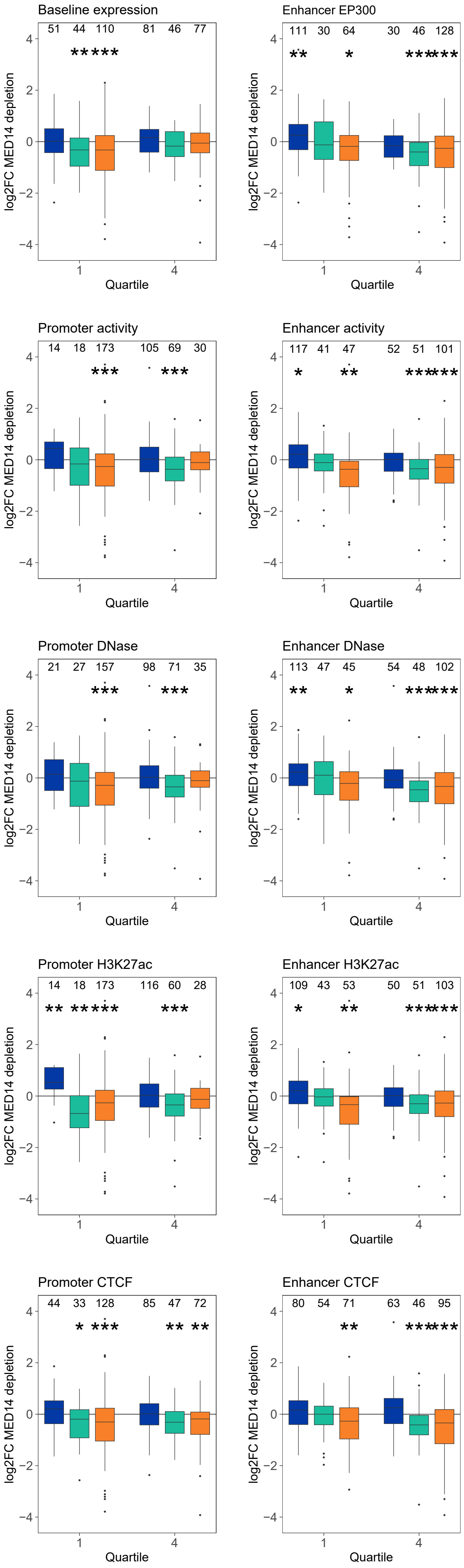

Figure S6. Features of MED14 responsive genes, related to Figure 3. Comparison of response to MED14 depletion for genes connected to enhancers with specific features. For every feature, the strongest enhancer per gene in the short, mid, and long categories were pooled and ranked on the feature. Then, the lowest 25% (quartile 1) and the highest 25% (quartile 4) of these enhancers were selected, and the response of their connected genes to MED14 depletion was depicted as log2FC. p-values calculated with 2-sided Wilcoxon rank-sum test of the specific group versus the no enhancer genes. ns=non-significant, *=p<0.05, **=p<0.01, ***=p<0.001. Only for visualization purposes, genes with a log2FC outside the (-4,4) range were not plotted.

Figure S7

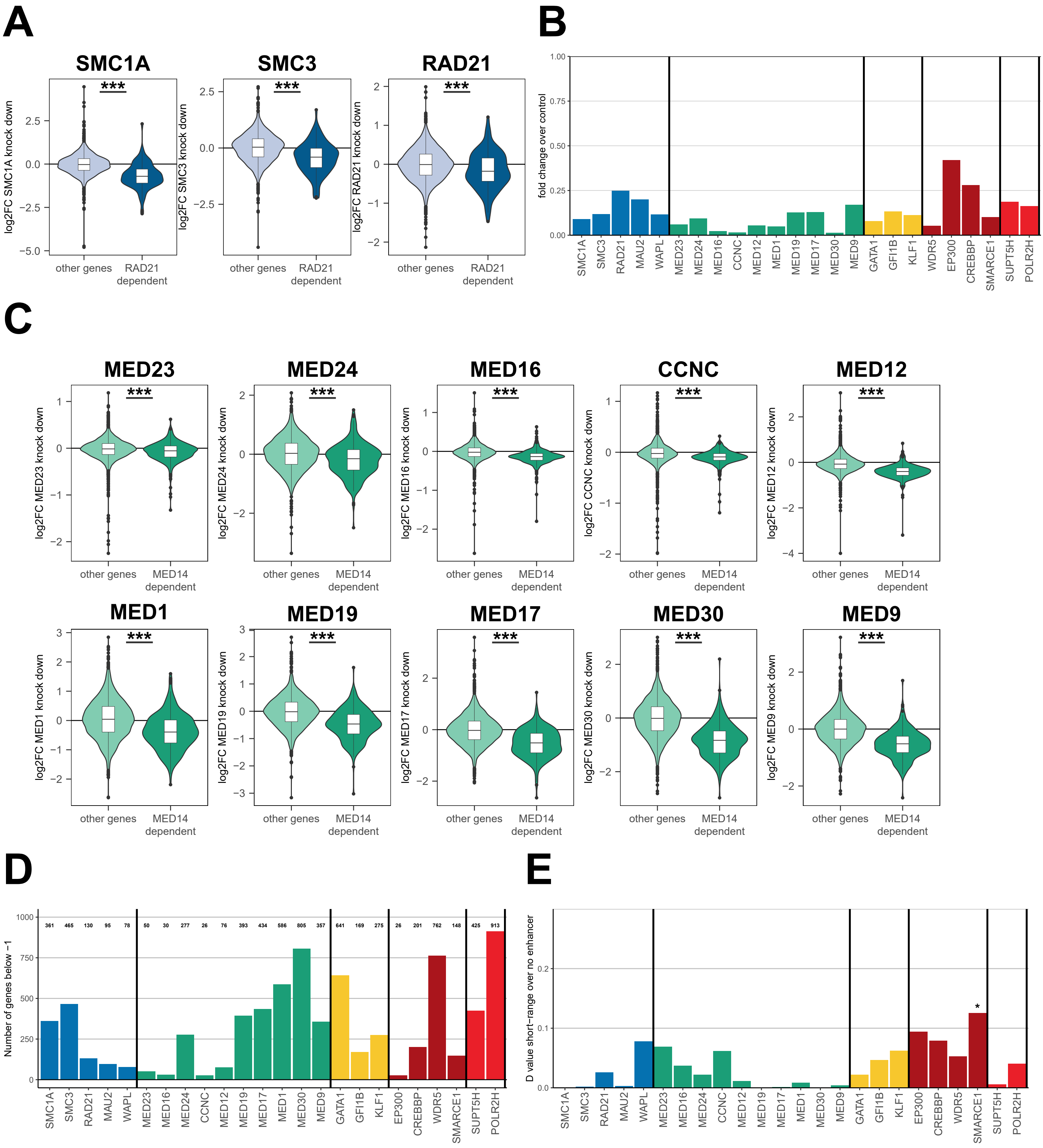

Figure S7. CRISPRi knock down and RNA-seq of regulatory factors, related to Figure 4. (A) log2 fold change of genes downregulated after acute RAD21 depletion (RAD21 dependent, log2FC <-0.8, FDR<0.05), compared to all other genes (RAD21 independent), after knock down of SMC1A, SMC3 or RAD21. 2-sided Wilcoxon rank-sum test, ***=p<0.001. **(B)** Fold change of target factors after knock down, compared to a non-targeting control sgRNA. **(C)** log2 fold change of genes downregulated after acute MED14 depletion (MED14 dependent, log2FC <-0.8, FDR<0.05), compared to all other, after knock down of subunits of the mediator complex. 2-sided Wilcoxon rank-sum test, ***=p<0.001. **(D)** Number of genes downregulated after target knock down, log2 fold change < -0.8. **(E)** Komolgorov-Smirnov D values of gene sets after knock down of target factors, short-range genes compared to no enhancer genes. One-sided test, *=p<0.05.

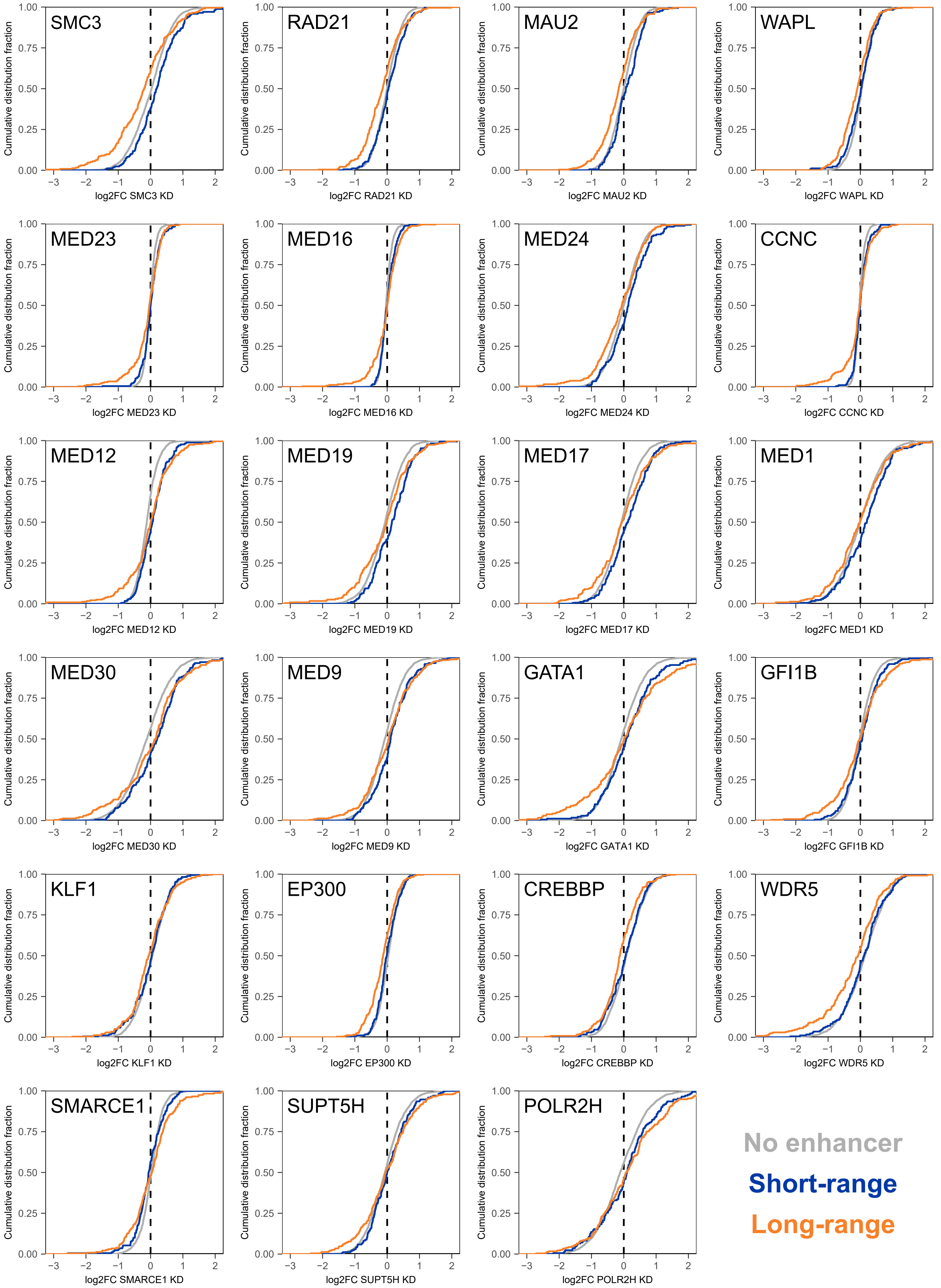

Figure S8. Cumulative distribution curves of log2 fold changes of enhancer-categorized genes after knock down of factors, related to Figure 4.

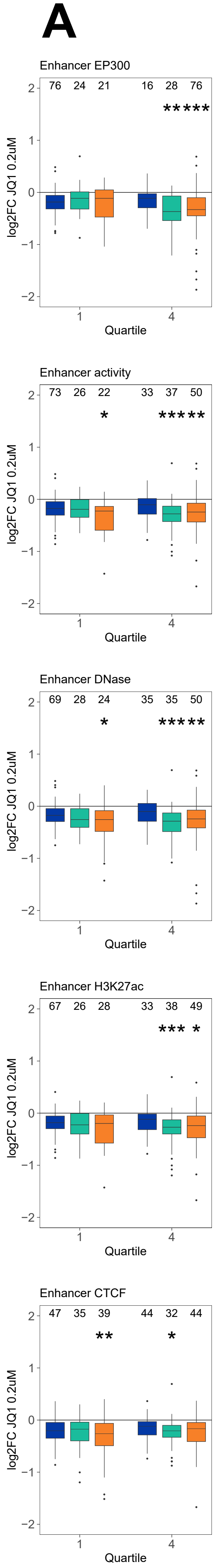

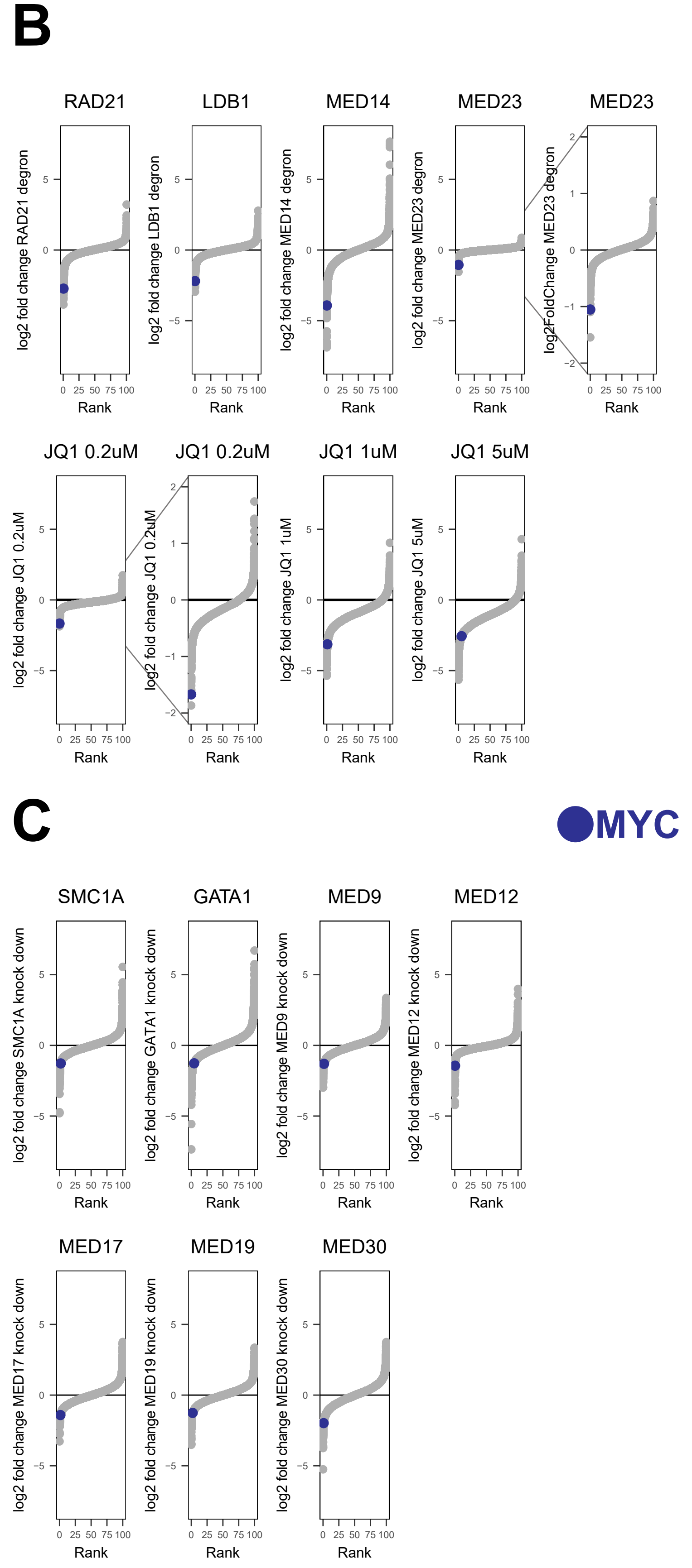

Figure S9. Features of JQ1 responsive genes, and MYC, related to Figure 4. (A) Comparison of response to JQ1 treatment (200nM) for genes connected to enhancers with specific features. For every feature, the strongest enhancer per gene in the short, mid, and long categories were pooled and ranked on the feature. Then, the lowest 25% (quartile 1) and the highest 25% (quartile 4) of these enhancers were selected, and the response of their connected genes to JQ1 treatment was depicted as log2FC. p-values calculated with 2-sided Wilcoxon rank-sum test of the specific group versus the no enhancer genes. ns=non-significant, *=p<0.05, **=p<0.01, ***=p<0.001. **(B)** All genes in nascent RNA-seq after acute factor perturbations or JQ1 treatment, or **(C)** all genes in total RNA-seq after CRISPRi factor knock down, were ranked on log2 fold change, and MYC was plotted. Ranks were plotted as a percentage of the total number of genes in the data set.
