# Supplementary material for: Long-range enhancer-controlled genes are hypersensitive to regulatory factor perturbations
Source: Cell Genom. 2025 Feb 25;5(3):100778. doi: 10.1016/j.xgen.2025.100778 (PMC11960515; doi:10.1016/j.xgen.2025.100778)
Supplement: Document S2. Article plus supplemental information [file mmc5.pdf]

Cell Genomics

Long-range enhancer-controlled genes are hypersensitive to regulatory factor perturbations

Graphical abstract

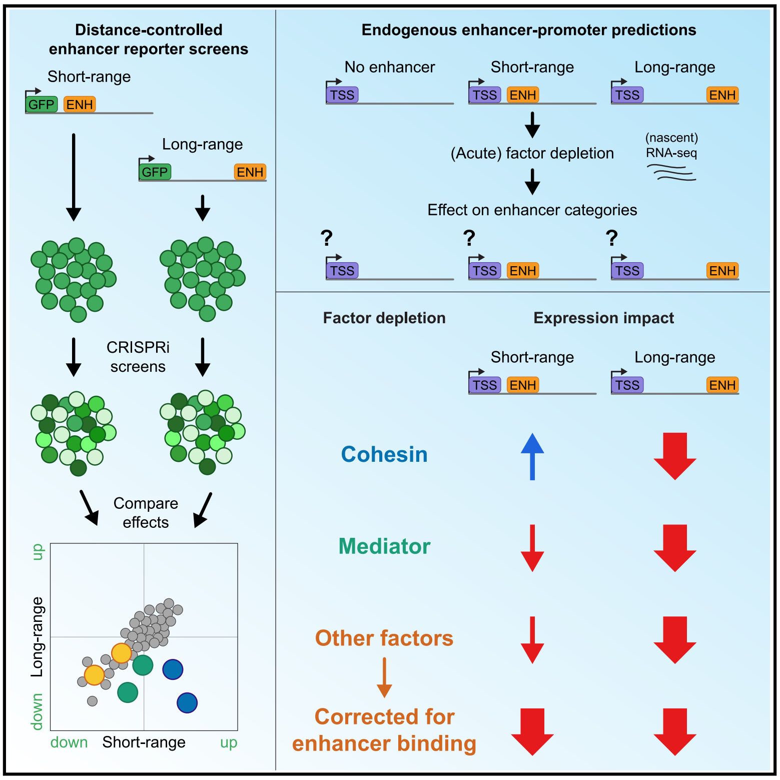

Highlights

- Reporter screens identify factors specific for long-range gene activation
- Impact of factor depletion on enhancer-distance-stratified endogenous genes
- Cohesin has long-range specific effect; mediator behaves long-range biased
- Distal enhancer-connected genes are hypersensitive to regulatory perturbations

Authors

Sjoerd J.D. Tjalsma, Niels J. Rinzema, Marjon J.A.M. Verstegen, ..., Mauro J. Muraro, Peter H.L. Krijger, Wouter de Laat

Correspondence

w.l.delaat@umcutrecht.nl

In brief

Tjalsma et al. employ distance-controlled enhancer reporter screens, acute factor depletion, and computational predictions to reveal how cohesin, mediator, and other factors govern distal enhancer-driven gene activation. Their findings show that genes contacting distal enhancers are sensitized to the disruption of regulatory factors.

Tjalsma et al., 2025, Cell Genomics 5, 100778
March 12, 2025 © 2025 The Author(s). Published by Elsevier Inc.
https://doi.org/10.1016/j.xgen.2025.100778

CellPress

Cell Genomics

CellPress
OPEN ACCESS

Article

Long-range enhancer-controlled genes are hypersensitive to regulatory factor perturbations

Sjoerd J.D. Tjalsma,[1] Niels J. Rinzema,[1] Marjon J.A.M. Verstegen,[1] Michelle J. Robers,[1] Andrea Nieto-Aliseda,[1] Richard A. Gremmen,[1] Amin Allahyar,[1] Mauro J. Muraro,[2] Peter H.L. Krijger,[1] and Wouter de Laat[1,3,*]
[1]Oncode Institute, Hubrecht Institute-KNAW and University Medical Center Utrecht, Utrecht, the Netherlands
[2]Single Cell Discoveries, Utrecht, the Netherlands
[3]Lead contact
*Correspondence: w.l.delaat@umcutrecht.nl
https://doi.org/10.1016/j.xgen.2025.100778

SUMMARY

Cell-type-specific gene activation is regulated by enhancers, sometimes located at large genomic distances from target gene promoters. Whether distal enhancers require specific factors to orchestrate gene regulation remains unclear. Here, we used enhancer distance-controlled reporter screens to find candidate factors. We depleted them and employed activity-by-contact predictions to genome-wide classify genes based on enhancer distance. Predicted distal enhancers typically control tissue-restricted genes and often are strong enhancers. We find cohesin, but also mediator, most specifically required for long-range activation, with cohesin repressing short-range gene activation and prioritizing distal over proximal *HBB* genes competing for shared enhancers. Long-range controlled genes are also most sensitive to perturbations of other regulatory proteins and to BET inhibitor JQ1, this being more a consequence of their distinct enhancer features than distance. Our work predicts that lengthening of intervening sequences can help limit the expression of target genes to specialized cells with optimal *trans*-factor environments.

INTRODUCTION

Transcription regulation is a complex process where the integration of multiple regulatory layers ensures correct tuning of gene expression.[1] The activation of specific genes is coordinated in a spatiotemporal manner by *cis*-regulatory elements called enhancers.[2] These non-coding sequences in the genome typically act in a cell-type-specific manner. They recruit tissue-specific transcription factors (TFs) and general cofactors, such as the mediator complex, that support the loading and activation of RNA polymerase II (RNAPII) on target promoters of cell-type-specific genes.[3,4] Although many enhancers are located in close linear proximity to their target gene, enhancer-promoter (E-P) combinations that are separated by long stretches of non-coding DNA are also well documented.[5] Distance between regulatory elements seems associated with biological complexity. For example, bacteria[6,7] and yeast cells[8] contain upstream activating sequences (UASs) maximally separated by a few hundred base pairs, while *Drosophila melanogaster* and mammalian enhancers reach distances to their target promoters of, respectively, tens to hundreds of kilobases.[9–11] The exact reason for the sometimes substantial linear separation of regulatory elements in the mammalian genome remains unclear, as does the precise orchestration of their communication. A major aspect of enhancer-mediated gene activation concerns the three-dimensional organization of the genome. In many cases, physical interactions between distal enhancers and promoters are coupled to gene activity.[9,12] Therefore, understanding the interplay among genome organization, the connection of distal regulatory elements, and the associated transcriptional output is highly relevant for understanding how cells activate and maintain specific gene expression programs.

During interphase, the multi-protein cohesin complex organizes genome folding via a process called loop extrusion. Cohesin forms and extrudes loops when loaded onto DNA,[13] a process that is constrained by CCCTC-binding factor (CTCF) binding at convergently oriented sites.[14,15] CTCF sites typically demarcate domains showing preferential self-interactions, or topologically associating domains (TADs).[16,17] Cohesin-mediated loop extrusion is highly dynamic,[18–20] and cohesin removal from cells rapidly results in severe disruption of chromosome folding,[21] especially at the level of CTCF boundaries.[22,23] Enhancers and target genes typically localize in the same TAD,[24] and CTCF-bound TAD boundaries serve to insulate genes from enhancers located in neighboring TADs.[25] Therefore, ongoing chromatin loop formation within TADs mediated by cohesin provides an attractive model to explain how linearly separated regulatory elements are able to contact and functionally communicate. How cohesin function relates to transcription regulation therefore has been heavily scrutinized in recent years, but it is not yet fully resolved. It has been documented at selected loci that the local manipulation of CTCF sites and loop extrusion can perturb

This is an open access article under the CC BY license (http://creativecommons.org/licenses/by/4.0/).

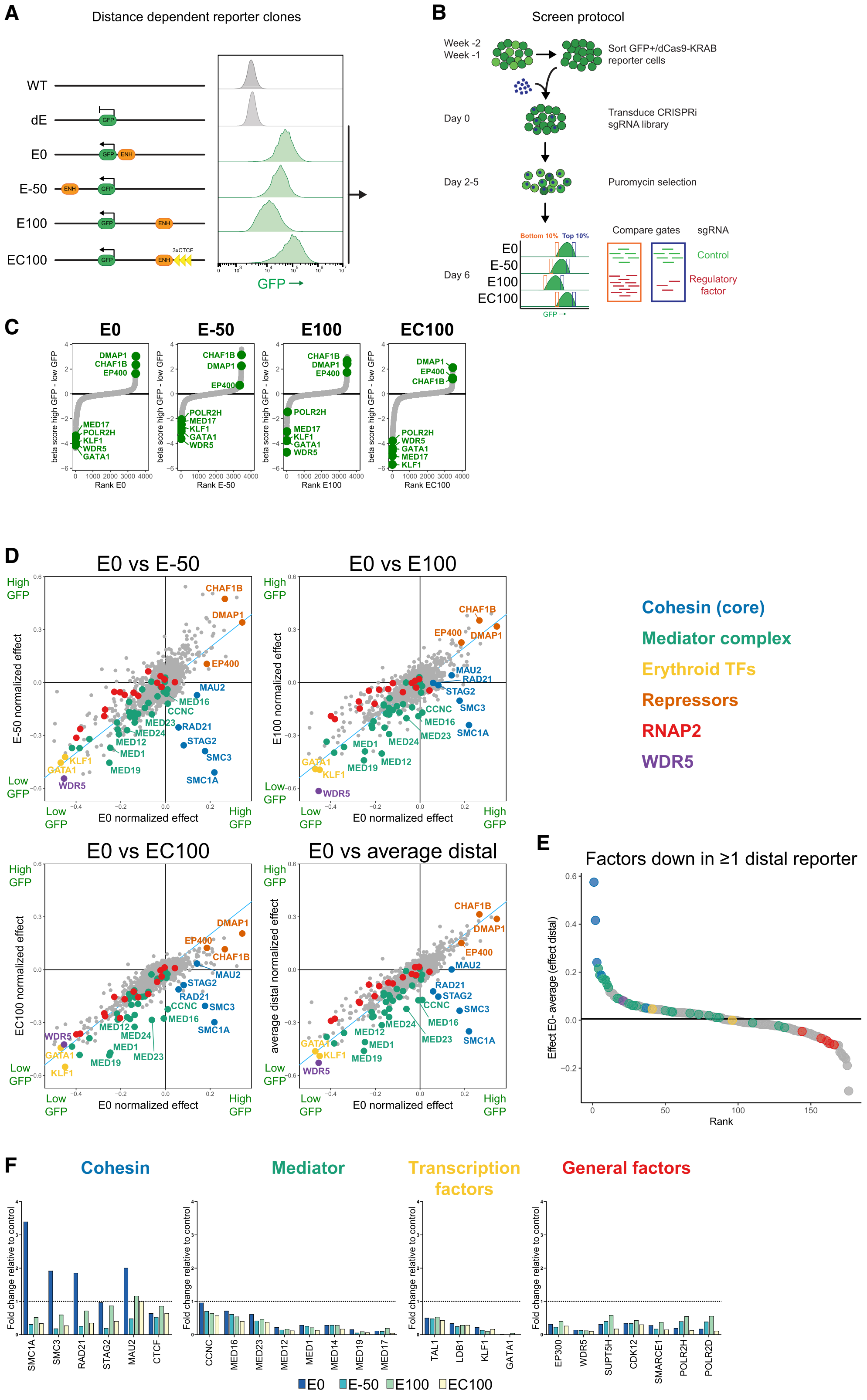

(legend on next page)

gene expression[26–28] and that, in particular settings, this has the capacity to drive disease[25,29] or cause developmental defects.[30] However, transcriptional activity of the majority of genes seems insensitive to global disruption of either cohesin or CTCF,[21,23,31] questioning the importance of loop extrusion and contact domains for gene regulation. Although a growing collection of defined E-P links has been studied in great depth, one reason it has remained difficult to dissect the interplay between genome organization and transcription and define factors supporting long-range gene activation is the complexity of linking enhancers to their cognate promoter. Using systems to vary the genomic distance between selected E-P pairs known to functionally interact, we and others have recently shown that their increased linear separation negatively impacts transcriptional output. Increased enhancer distance also creates a dependence on cohesin for enhancer-mediated gene activation.[24,32,33]

Here, we used our K562 cell lines with an enhancer at varying distances from a reporter gene, to search for *trans*-acting factors specifically supporting long-range enhancer-mediated gene activation. Using CRISPR interference (CRISPRi) screens in the different cell lines, we identify various cohesin components that are uniquely necessary for long-range, but obsolete for (even hindering) short-range, controlled reporter gene activation. Many mediator components appear unique in being long-range biased, having a measurable impact across all enhancer distances, but having a stronger impact on the long-range controlled reporter genes. To extrapolate these reporter gene-based observations to the regulation of endogenous genes, we used activity-by-contact predictions on functional E-P pairs in human K562 cells to classify genes as short-, mid-, or long-range enhancer-controlled genes or enhancer-independent genes. RNA sequencing (RNA-seq) in CRISPRi-depleted cells, as well as nascent RNA-seq following acute cohesin depletion, confirmed genome-wide that cohesin is particularly required for long-range enhancer-controlled gene expression, but that it hindered short-range enhancer-controlled gene activity. Furthermore, we show that long-range enhancer-connected genes are the most sensitive genes to various enhancer-targeting perturbations.

RESULTS

Screening for factors involved in long-range enhancer-mediated gene activation

To search for *trans*-acting factors that specifically support long-range enhancer-mediated gene activation, we utilized previously described K562 cell lines with a GFP reporter gene driven by the *HBB*-like *HBG1* promoter.[32] The reporter gene was integrated in a repressive chromatin domain and relied for expression on the presence of the locus control region (μLCR), a compact version of the *HBB* super-enhancer, which was integrated at varying distances (0-50-100 kb) from the reporter gene. Besides the three published cell lines E0, E100, and EC100, the latter having three strong CTCF binding sites inserted immediately downstream of—and oriented toward—the E100, we included a new cell line (E50) that had the μLCR integrated 50 kb away on the other side (downstream) of the reporter gene, to also test whether we could identify orientation-specific regulatory factors (Figure 1A). In each of these cell lines, we performed a CRISPRi screen with a custom-made single-guide RNA (sgRNA) library composed of ~18,000 sgRNAs targeting 3,200 nuclear protein-encoding genes (5 sgRNAs per transcription start site [TSS], plus 624 non-targeting sgRNAs). After library transduction, we isolated the top and bottom 10% GFP-expressing cells and calculated the enrichment of sgRNAs in the low versus high GFP populations, to analyze per cell line the impact of each factor on reporter gene expression (Figure 1B).

Despite the inherent experimental limitations of screens (one will miss factors if they are essential for cell survival, or if guides fail to target or silence a gene), they identified a large number of factors that were required for *HBG* gene expression in all four reporter cell lines, including many expected factors. For example, a control sgRNA against the *HBG* promoter driving GFP reporter expression was strongly enriched in all low GFP cell populations and depleted from cells with high GFP (Figure S1A). The same was true for sgRNAs targeting different RNAPII subunits and mediator components, as well as the splicing factor 3 (SF3) and small nuclear ribonucleoprotein particle (snRNP) splicing complexes (shown to impact transcription of intron-less genes[34–36]; Figures 1C and S1B–S1D). Among the factors with the strongest effect on reporter expression were WDR5, which associates with and is required for H3K4 methylation,[37] as well as the erythroid-specific TFs GATA1 and KLF1, which are known to bind to both the enhancer and the *HBG* promoter and to regulate *HBG* gene expression.[38,39] Noteworthy, besides activators, we identified factors that acted as repressors, since their knockdown reproducibly led to increased reporter gene expression. Among them were components of the Tip60-p400 complex (also known as the NuA4 histone acetyltransferase complex), a transcriptional repressor complex harboring the DNA

Figure 1. Distance-controlled reporter screens in K562 cells

(A) K562 cell lines used in this study. Promoter and enhancer elements were introduced with a GFP reporter in an inactive locus in the K562 genome.[32]

(B) Experimental setup of CRISPRi screens. Reporter cell lines expressing dCas9-KRAB were transduced with an sgRNA library targeting the TSSs of genes of interest. After selection, reporter lines were sorted for the 10% lowest and highest GFP signals, followed by sequencing. Five sgRNAs per target TSS were used.

(C) Ranking plots of enrichment scores of the different reporters. Scores were determined by subtracting the MAGeCK beta score of the GFP-high population from that of the GFP-low population per reporter line.

(D) Scatterplots of enrichment scores, normalized to an sgRNA directly targeting the HBG-GFP promoter. The scores in the E0 reporter were compared to the E50 (top left), E100 (top right), EC100 (bottom left), or the average of the scores in the E50, E100, and EC100 (average distal, bottom right).

(E) Ranking plot comparing the average distal enrichment score with the E0 score for factors affecting distal reporter expression. Factors with a normalized score of ≤ -0.1 in at least one distal gate were compared. Their average distal score was subtracted from the score in E0 and factors were ranked on this value.

(F) Single-guide validation of screen results. Values plotted are the fold change (FC) of a specific sgRNA compared to the average of two control sgRNAs. $n = 1$ per knockdown.

See also Figure S1 and Tables S1 and S2.

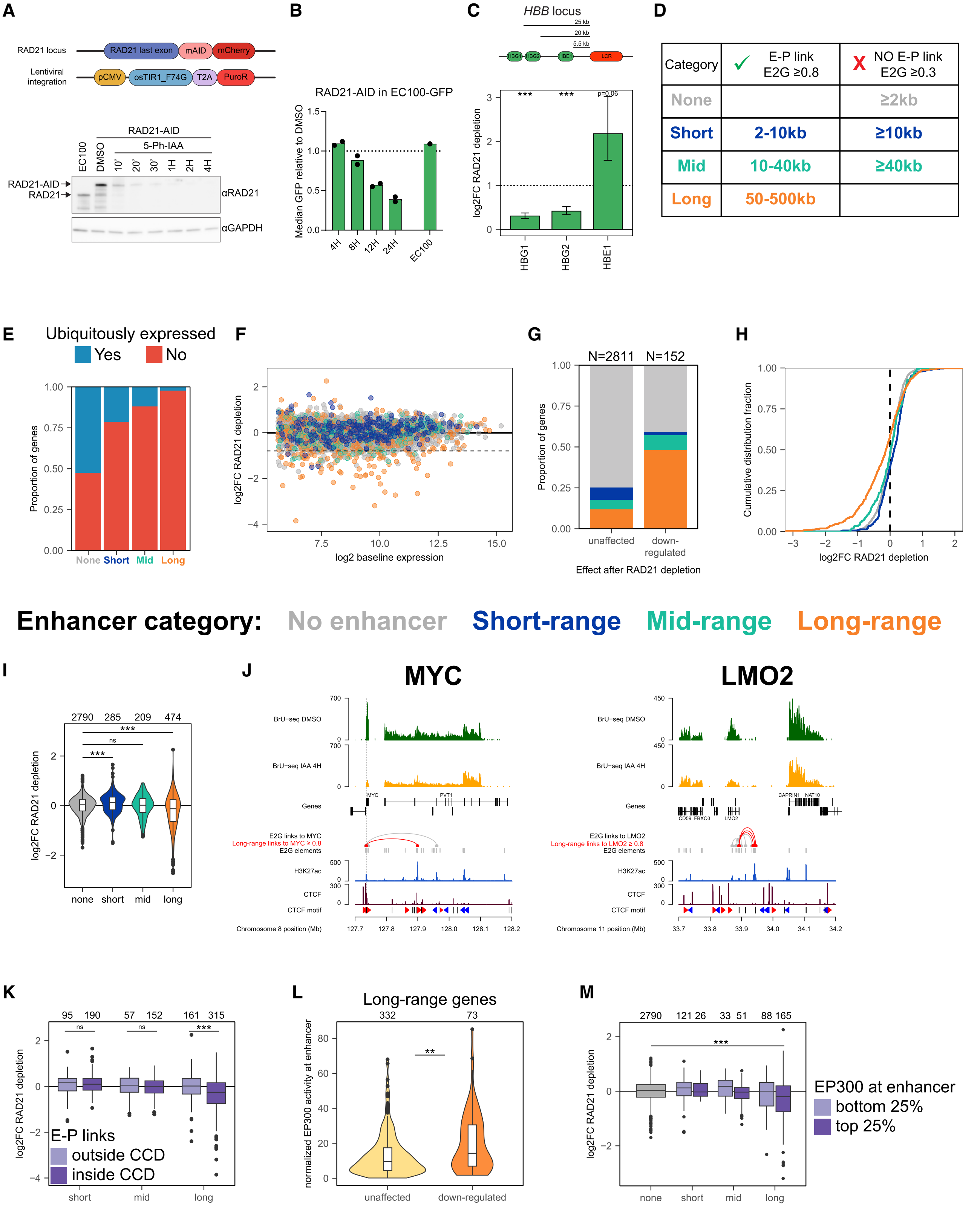

(legend on next page)

methyltransferase DMAP1[40] (Figure S1E), and both components of the chromatin assembly factor complex CAF-1, previously implicated in safeguarding cell identity through the suppression of enhancers.[41,42] Thus, each of the screens successfully identified many factors with anticipated impact on reporter gene transcription.

To uncover candidate factors with specific impact on long-range enhancer-mediated gene activation, we compared the sgRNA enrichment scores of, respectively, E50, E100, and EC100, or the average of these three distal reporters, with those of the E0 cell line (Figures 1D, 1E, and S1F). To account for differences in effect sizes between the cell lines, likely due to the range of reporter GFP levels, we normalized gRNA scores by the enrichment of the *HBG* TSS gRNA directly targeting the reporter. Four core components of the cohesin complex (SMC1A, SMC3, RAD21, and STAG2) stood out as being exclusively required for long-range enhancer activation in E50, E100, and EC100. In fact, all four factors had an opposite, inhibitory effect on reporter gene expression in E0. Additionally, we observed that there were factors that affected E-P communication over all distance ranges, but with an apparent stronger effect on the long-range controlled reporters (Figure 1D). This was particularly true for the many components of the mediator complex (Figures 1E and S1F, green).

For validation, we performed a series of CRISPRi knockdown experiments, targeting many of these factors individually in all four cell lines. For all tested factors, the reporter genes responded as predicted from the screen results (Figure 1F). Thus, the reporter was upregulated in E0 and downregulated in E50, E100, and EC100 upon knockdown of the core cohesin components SMC1A, SMC3, and RAD21, and upon depletion of MAU2, a cohesin loading and processivity factor. A similarly long-range specific, but milder, transcriptional response was observed following the depletion of STAG2, another core cohesin component. Following the knockdown of eight different mediator components, the general trend was that wild-type mediator levels were more important for the longer-range E-P reporters. Targeted knockdown of the tissue-specific TFs TAL1, LDB1, KLF1, and GATA1 and of a series of more general factors known to act on enhancers and/or promoters, caused reporter gene downregulation in all four configurations but without a clear bias toward a more short- or long-range configuration (Figure 1F).

We concluded that our screens reliably identified factors with general and distance-dependent impact on the four E-P reporter configurations. The cohesin complex uniquely stood out as long-range specific, while mediator components showed a long-range biased impact on reporter gene expression.

Cohesin is specifically required for genes controlled by distal enhancers

The screens employed CRISPRi depletion of factors, measuring transcriptional responses 6 days after initiating protein depletion, which may give rise to confounding indirect effects that influence results. To better enable studying the direct contribution of cohesin, we generated an AID2 degron cell line for its subunit RAD21 in the EC100 reporter line (Figure S2A). As described,[43] addition of 5-Ph-IAA (hereafter, IAA) to the medium induced rapid degradation of RAD21 (Figure 2A). As expected, it also caused reduction of EC100 reporter expression (Figure 2B). 4C-seq showed that this was accompanied by a rapid collapse of long-range chromatin contacts in the reporter locus (Figure S2B).

To measure the effect of acute perturbation of cohesin levels on transcription in the genome, we performed nascent RNA-seq (bromouridine [BrU]-seq) after 4 h of RAD21 depletion. In our reporter experiments based on *HBB* gene regulatory elements, we observed that cohesin supported expression only when the enhancer was located at a distance. Interestingly, we found a similar impact of cohesin on gene regulation at the endogenous *HBB* locus. K562 cells express the embryonic (*HBE1*) and fetal (*HBG1*/*HBG2*) *HBB*-like genes, which are activated by the LCR, but with *HBE1* being located more proximal to the LCR than the *HBG* genes. After acute depletion of RAD21, we observed that the distal *HBG1*/*HBG2* genes were downregulated, while proximal *HBE1* expression was increased

Figure 2. RAD21 controls expression of genes connected to long-range enhancers

(A) Generation and validation of RAD21-AID line in K562 EC100 reporter cells. Top: schematic of AID targeting strategy. Bottom: western blot validation of acute RAD21 depletion, one representative clone. $n = 2$ clonal cell lines were used for all degron experiments.

(B) FACS measurement of reporter GFP after RAD21 depletion, normalized to DMSO. Parental EC100 was used as a control to exclude the effect of IAA ligand on reporter after 24 h of treatment.

(C) Effect of RAD21 depletion on endogenous *HBB* genes plotted as FC. Error bars represent standard error. ***$p < 0.001$, false discovery rate (FDR)-adjusted Wald test.

(D) Categorization of enhancer genes based on ENCODE-rE2G model.

(E) Fraction of genes per category that was designated ubiquitously expressed in E2G dataset.

(F) BrU-seq after 4 h of acute RAD21 depletion. Only genes categorized as in (D) are plotted. Y axis depicts log2FC after RAD21 depletion; x axis depicts counts at baseline expression (DEseq2 baseMean).

(G) Proportion of genes per category that was found downregulated (log2FC < -0.8, FDR <0.05) or unaffected ($-0.2 <$ log2FC < 0.2) by RAD21 depletion.

(H) Cumulative distribution curves of log2FCs of enhancer-categorized genes after RAD21 depletion.

(I) Violin plots of log2FCs per enhancer category after RAD21 depletion.

(J) Examples of long-range connected genes affected by RAD21 depletion. In red arcs, long-range E2G links with an E2G score ≥ 0.8. Dotted line represents the location of the TSS of *MYC* and *LMO2*.

(K) Log2FC per category. Within each group, outside CCD links were compared to inside CCD links.

(L) EP300 levels on long-range enhancers connected to unaffected or downregulated genes after RAD21 depletion.

(M) Log2FC per enhancer category, separated on the enhancers being in the lowest or highest 25% of EP300-enhancer levels. Enhancer-regulated groups were compared to no-enhancer genes. Only the p value for the group significantly downregulated compared to no-enhancer genes is displayed.

For (I) and (K)–(M), p values determined with two-sided Wilcoxon rank-sum test. ns, non-significant; *$p < 0.05$; **$p < 0.01$; ***$p < 0.001$. See also Figures S2–S4.

(Figure 2C). This supported the idea that cohesin helps enhancers to act over a distance and showed that cohesin itself has no impact on the intrinsic activity of enhancers.

To study the impact of cohesin and other factors on the regulation of endogenous enhancer-controlled genes in the genome, we aimed to define long- and short-range enhancer-controlled genes in K562 cells. For this, we employed the latest version of the activity-by-contact (ABC) model,[44] called ENCODE-rE2G (enhancer-to-gene), hereafter referred to as E2G model.[10] This model has been trained by machine learning on ENCODE data to combine epigenomics data like enhancer activity and accessibility marks ("activity") with Hi-C E-P contact frequencies ("contact"), to quantify E-P links. E-P pairs thereby receive an E2G score ranging from 0 to 1, being a predictive measure for the regulatory relevance of an E-P pair. Performance of the E2G model was extensively benchmarked on multiple large datasets of functionally validated E-P pairs, providing remarkable accuracy, obtaining an area under the precision recall curve of 0.76, with 70% precision at 70% recall.[10]

We used E2G predicted functional E-P pairs in K562 cells to distinguish four categories of expressed genes (Figure 2D). First are the promoter-autonomously expressed genes (n = 2,790), which are genes without a link to an enhancer with an E2G score $\geq$ 0.3. Second and third are the short-range (n = 285) and mid-range (n = 209) controlled genes, which are genes linked to an enhancer with E2G $\geq$ 0.8 at, respectively, 2–10 kb (short) or 10–40 kb (mid) from the promoter and without having links with E2G $\geq$0.3 to more distal enhancers. The fourth category was defined as long-range controlled genes, which are genes (n = 474) that had a link to at least one enhancer (E2G $\geq$ 0.8) at a distance between 50 and 500 kb from the promoter (Figure 2D). Genes that did not specifically fit one of these categories (e.g., genes contacting multiple enhancers with lower E2G scores and at variable distances) were considered ambiguous and therefore ignored.

We analyzed these gene categories in more depth. Approximately half of the promotor-autonomously expressed genes were ubiquitously expressed, while the enhancer-contacting genes were increasingly more tissue restricted the farther they were separated from their enhancer (Figure 2E). We then considered a large series of enhancer and promoter features that were provided in the E2G dataset for all E-P pairs, including their promoter and enhancer activity, their H3K27Ac, DNase, or EP300 signal and other features.[10] Based on this information, long-range controlled genes appeared to have weaker promoters than short-range controlled genes, but they were connected to stronger enhancers, with higher EP300 and H3K27Ac levels and higher DNase signal (Figure S3). This aligns well with a recent study showing that intrinsic enhancer strength is critical for them to activate genes over larger distances.[45]

Intersecting the four gene categories with the genes found downregulated upon acute RAD21 depletion revealed that the long-range enhancer-controlled genes were highly enriched among the RAD21 target genes (Figures 2F–2H). This was also the only category that was collectively downregulated after RAD21 depletion (Figure 2I). As examples, *MYC*, *LMO2*, and *MS4A3* were among the long-range defined genes that were found downregulated upon cohesin depletion (Figure 2J). The effect on their expression was associated with a loss in domain structure and reduction in E-P interactions (Figures S2C–S2E).

For short-range genes, we observed the opposite: acute RAD21 depletion caused a significant increase in their gene expression as compared to other categories of genes (Figure 2I), confirming our observations in the reporter cell lines (Figures 1D and 2C[32]).

We then analyzed whether promoter or enhancer features were additionally distinctive for the cohesin-responsive long-range genes. Two features stood out. First, it was particularly those long-range controlled genes of which the enhancer was located within the same CTCF-contact domain (CCD) that were affected by the loss of cohesin. CCDs strongly overlap with TADs,[46] and genes connected to a distal enhancer inside these domains were significantly more downregulated than those with the distal enhancer outside their CCD (Figure 2K). Second, enhancer strength, in particular, the EP300 level, was significantly higher for responding than non-responding long-range genes (Figures 2L and S4A). When stratified for the different enhancer and promoter features, short-range genes remained insensitive for cohesin depletion, implying that it is not simply the different nature of long-range enhancers that explains their sensitivity to cohesin (Figures 2M and S4B).

Collectively these results showed that the typical gene that requires cohesin for activated expression has a distal enhancer with high activity (high EP300, H3K27Ac, and DNase levels) located in the same CCD. In general, cohesin does not support and even represses transcription activation when genes are controlled by a nearby enhancer.

Genes controlled by distal enhancers are also more sensitive to perturbations in mediator levels

We wished to use this approach to analyze whether there were other factors with a dominant effect on distal enhancer-controlled genes. In our CRISPRi screens, we identified subunits of the mediator complex as having a bias for long-range genes. Therefore, we generated a degron line for the tail subunit MED23, one of the subunits with the strongest distal bias in our screens (Figures 1D and S5A–S5D). However, the effect size of acute degradation of MED23 in our cells was very small, possibly due to redundancy among mediator components[47] or to suboptimal depletion (Figure S5B). Nevertheless, we observed that the direct target genes of MED23 were enriched for long-range controlled genes (Figures 3A and S5D). To further investigate this for the mediator complex, we analyzed published thiol(SH)-linked alkylation for the metabolic sequencing of RNA (SLAM)-seq data (nascent RNA-seq) collected 4 h after acute depletion of the core mediator subunit MED14 from K562 cells (Figure 3B).[48] To quantify gene set enrichments in transcriptomes, we calculated the Kolmogorov-Smirnov D (distance) value as a measure of the degree and significance by which a category of genes was overrepresented among the downregulated genes (Figure 3C). In contrast to RAD21, MED14 depletion significantly affected both long- and mid-range-controlled genes (Figure 3C). MED14 depletion had no significant impact on short-range controlled genes even when stratified for distinctive

CellPress
OPEN ACCESS

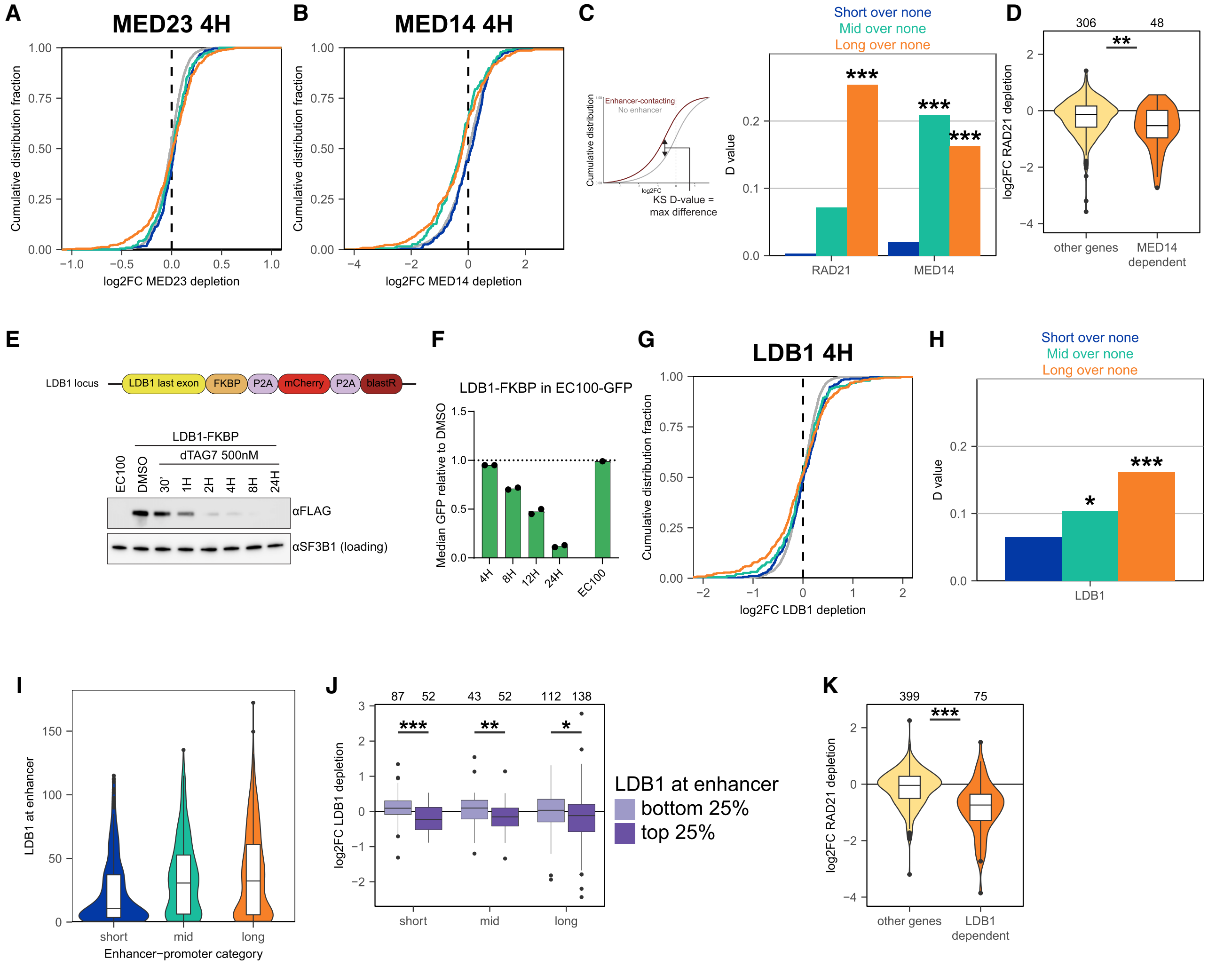

Figure 3. Regulation of distal-enhancer connected genes by mediator and LDB1

(A and B) Cumulative distribution curves of log2FCs of enhancer-categorized genes after 4 h MED23 depletion (BrU-seq) (A) or 4 h MED14 depletion (SLAM-seq)[48] (B).

(C) Kolmogorov-Smirnov D value, and D values of enhancer categories compared to no-enhancer genes after acute RAD21 or MED14 depletion. One-sided Kolmogorov-Smirnov test was performed; $***p < 0.001$.

(D) Effect of RAD21 depletion for long-range genes downregulated after MED14 depletion (log2FC <-0.3, FDR <0.05) compared to unaffected long-range genes. Two-sided Wilcoxon rank-sum test; $**p < 0.01$.

(E) Schematic and western blot validation of LDB1-FKBP degron lines in K562 EC100 cells, one representative clone. $n = 2$ clonal cell lines were used for all degron experiments.

(F) FACS validation of EC100-GFP reporter after LDB1 depletion.

(G) Cumulative distribution curves of log2FC of enhancer-categorized genes in BrU-seq, after 4 h LDB1 depletion.

(H) Kolmogorov-Smirnov D values (one-sided test) after LDB1 depletion of enhancer genes versus no-enhancer genes. $*p < 0.05$; $***p < 0.001$.

(I) LDB1 level on enhancers based on LDB1 ChIP-seq.[49]

(J) Effect of acute LDB1 depletion stratified for LDB1 level present at the enhancers. Quartiles were determined by ranking all enhancers defined in the three categories on LDB1 level, and then genes were assigned to be connected to either the LDB1 lowest (light purple) or highest (dark purple) 25% LDB1 bound at the enhancer. Two-sided Wilcoxon rank-sum test; $*p < 0.05$; $**p < 0.01$; $***p < 0.001$.

(K) Effect of RAD21 depletion for long-range genes downregulated after LDB1 depletion (log2FC < -0.3, FDR < 0.05) compared to unaffected long-range genes. Two-sided Wilcoxon rank-sum test; $***p < 0.001$.

See also Figures S5 and S6.

promoter and enhancer features (Figure S6). Thus, genes regulated by more distal enhancers appeared more sensitive to perturbations in mediator levels than genes controlled by proximal enhancers. Among the long-range controlled genes, the MED14-sensitive genes were also significantly more sensitive to RAD21 depletion (Figure 3D). This showed that cohesin and mediator shared many of their long-range controlled target genes.

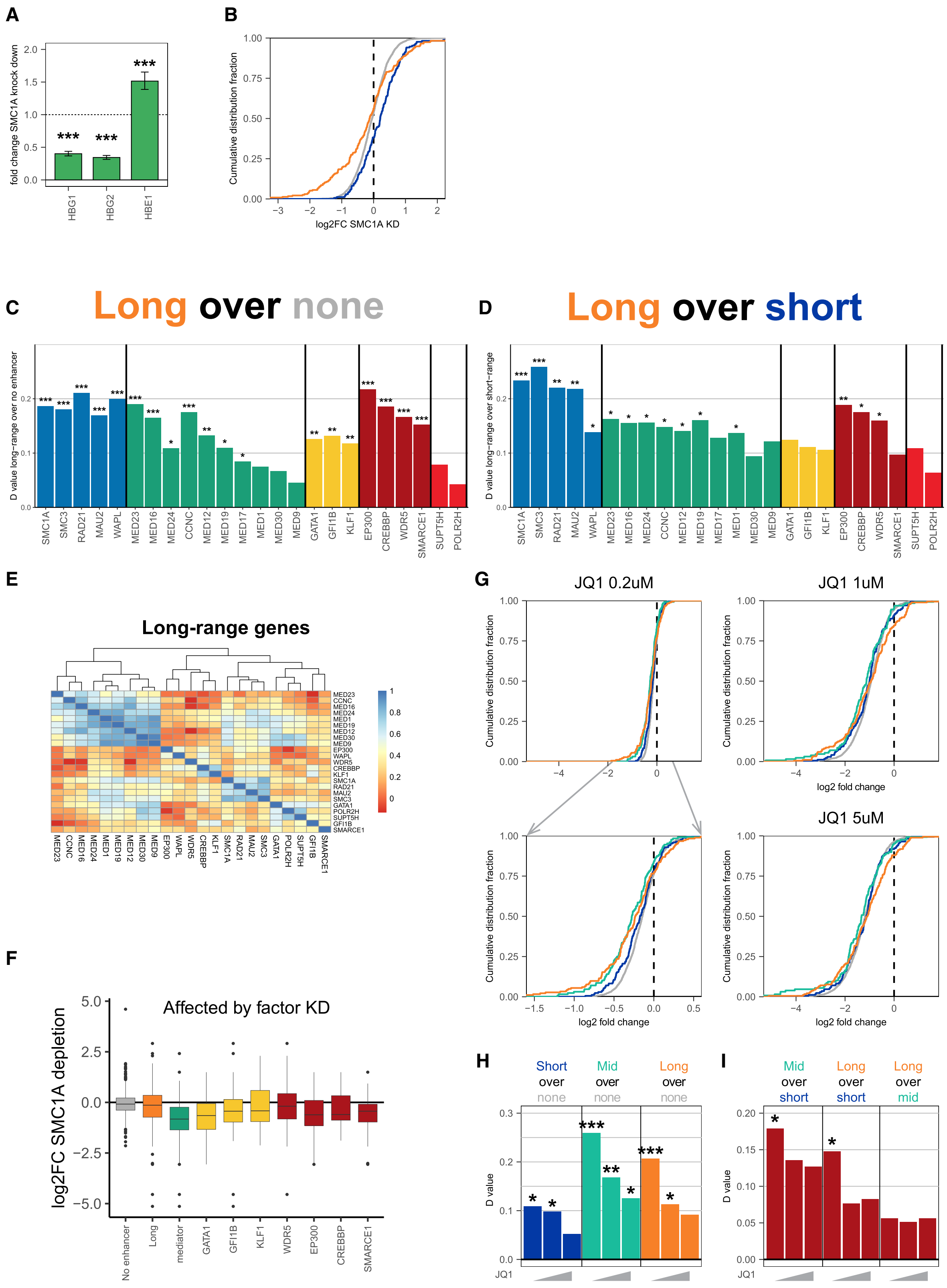

(legend on next page)

LDB1 accumulation at distal enhancers explains increased LDB1 sensitivity of long-range genes

We turned to LDB1, a tissue-specific transcription co-factor that has an important role in erythroid transcription and has been implicated in chromatin looping and long-range gene activation.[50,51] Targeted knockdown of LDB1 by CRISPRi strongly affected the expression levels of our reporter gene in a manner that seemed independent of the E-P distance (Figure 1F). We generated an FKBP degron line for LDB1 in our EC100 reporter cell line (Figures 3E and S5E) and confirmed that depletion of LDB1 led to reduced reporter GFP levels (Figure 3F). We then performed BrU-seq after 4 h of depletion and intersected the identified direct target genes of LDB1 with the aforementioned categories of enhancer-connected genes. As seen for cohesin and mediator, loss of LDB1 was foremost sensed by the long-range controlled genes, followed by the mid-range group (Figures 3G and 3H). To investigate this further, we used published LDB1 chromatin immunoprecipitation (ChIP)-seq data[49] and observed that long-range E-P pairs recruited the most and short-range pairs the least LDB1 to their enhancers (Figure 3I). To analyze whether this biased the results, we stratified all enhancer-controlled genes based on the amount of LDB1 bound to their enhancers, and then re-analyzed the responses of these genes to LDB1 depletion. LDB1 binding to the corresponding enhancer appeared highly predictive for genes to respond to the loss of LDB1 (Figure 3J). This can be considered to be independent evidence for the accuracy of the E2G model in predicting functional E-P pairs across distance ranges. It showed, however, that the increased sensitivity of long-range enhancer-controlled genes to LDB1 loss was mostly the consequence of these enhancers recruiting more LDB1 than their shorter-range counterparts. Indeed, LDB1 binding seemed associated with EP300 levels at enhancers (Figure S5F), suggesting that these are strong enhancers and that LDB1 binding may aid enhancer strength. Long-range target genes of LDB1 were collectively sensitive for cohesin perturbations (Figure 3K), implying that LDB1-mediated long-range gene activation often also depends on cohesin loop extrusion. 4C-seq applied to *MYC*, *LMO2*, and *MS4A3*, three loci with distal enhancers that structurally collapsed upon cohesin depletion (Figures S2C–S2E), revealed that the loss of LDB1 also induced a specific but more focal loss of contacts between the genes and LDB1-bound enhancers (Figures S5G–S5I). This confirmed the recent demonstration that LDB1 promotes E-P contacts genome-wide.[52] This same study also showed that cohesin may aid in the formation of a subset of LDB1-dependent loops, but that often these loops are formed independent of cohesin. Our example loci appeared to require both cohesin and LDB1 for efficient E-P interactions and for activated gene expression.

Long-range enhancer-controlled genes are exquisitely sensitive to *trans*-factor perturbations

We then wanted to investigate for a larger set of transcription-related factors whether a reduction in their protein levels was foremost sensed by long-range enhancer-controlled genes. We therefore turned to CRISPRi knockdown experiments as they offered an easier and faster alternative to the creation of many different degron lines. We first tested whether CRISPRi experiments were suitable for this analysis by repeating the CRISPRi knockdown experiments for cohesin, targeting SMC1A, SMC3, and RAD21 in the E50 cell line and performing RNA-seq. Genes that showed reduced expression immediately after acute RAD21 protein depletion were also found downregulated 6 days after CRISPRi knockdown of SMC1A, SMC3, and RAD21 (Figure S7A). As seen after acute RAD21 depletion, *HBB* genes showed a distance-dependent competitive response to the loss of cohesin; the gene most proximal to the LCR, *HBE1*, was upregulated, while the more distal *HBG* genes were downregulated (Figure 4A). As CRISPRi RNA-seq datasets may expose direct and indirect target genes of a depleted factor and may be noisier than BrU-seq datasets generated immediately after acute depletion, we simplified our genome-wide analysis by focusing only on three categories of genes: the long- and short-range enhancer-controlled genes and the promoter-autonomously expressed (no enhancer) genes. Also, we only considered E-P pairs present in the same CCD domain. With these criteria, we found also in all the cohesin CRISPRi knockdown transcriptomes an enrichment of long-range controlled genes among the (downregulated) target genes (Figures 4B and S8).

This encouraged us to use CRISPRi to analyze whether other factors had a specific impact on genes controlled by long-range enhancers. In addition to core cohesin subunits, we selected the cohesin auxiliary proteins MAU2 and WAPL; 10 mediator subunits; the cell-type-specific TFs GATA1, GFI1B, and KLF1; and several other factors associated with enhancer biology or transcription in general (EP300, CREBBP [CBP], WDR5, SMARCE1, SUPT5H, POLR2H). Following CRISPRi, RNA-seq was performed. In all cases, knockdown was confirmed to result in a strong reduction in transcripts of the gene of interest (Figure S7B). We confirmed that the direct target genes of MED14,

Figure 4. Genes contacting long-range enhancers are sensitized to perturbation of regulatory factors

(A) FC of *HBB* genes after SMC1A knockdown. Error bars represent standard error. ***$p < 0.001$, FDR-adjusted Wald test.

(B) Cumulative distribution curves of log2FCs of enhancer-categorized genes after SMC1A knockdown.

(C and D) Kolmogorov-Smirnov D values of gene sets after knockdown of target factors.

(C) Long-range genes compared to no-enhancer genes.

(D) Long-range genes compared to short-range genes. Only links within a CCD were used. $n = 2,614$ no-enhancer genes, 170 short-range genes, and 233 long-range genes.

(E) Pearson correlation of effect on long-range genes after knockdown of target factors.

(F) Log2FC after SMC1A knockdown of downregulated genes (log2FC < -0.5) of specific targets. No-enhancer: all no enhancer genes. Long: all long-range enhancer genes. Mediator: genes that have a log2FC < -0.5 in at least two mediator subunits.

(G) Cumulative distribution curves of log2FCs of enhancer-categorized genes after different doses of JQ1 treatment.

(H and I) Kolmogorov-Smirnov D values of gene sets after increasing doses of JQ1 treatment (0.2, 1, and 5 μM). One-sided tests. *$p < 0.05$; **$p < 0.01$; ***$p < 0.001$. See also Figures S7–S9.

identified immediately after its acute depletion (Figures 3B and 3C), were collectively downregulated in the CRISPRi transcriptomes of all 10 mediator subunits (Figure S7C), showing that CRISPRi transcriptomes still contained the signature of the mediator's direct target genes.

We then intersected all transcriptomes with the three categories of differently regulated genes. The depletion of factors involved in basal transcription (SUPT5H, POLR2H) each caused the downregulation of many genes (Figure S7D), but without any of these factors showing preference for a specific category of genes (Figures 4C and 4D). The downregulation of the chromatin remodeling component SMARCE1 also influenced both the short- and long-range enhancer-controlled genes more than the promoter-autonomous genes (Figures 4C, 4D, and S7E).

In contrast, and as seen for the cohesin core subunits SMC1A, SMC3, and RAD21, the auxiliary cohesin factors MAU2 and WAPL did have a pronounced and highly significant impact on long-range enhancer-controlled genes (Figures 4C and 4D). WDR5 has been implicated in development and is seen mostly at active gene promoters, where it binds H3K4me3.[37] Surprisingly, the depletion of WDR5 had a significantly higher impact on long-range controlled genes (Figures 4C and 4D). The same trend was also clearly visible for all other tested factors, which were previously reported to be associated with enhancer biology. Thus, most mediator subunits, the tissue-specific TFs GATA1, GFI1B, and (to a lesser extent) KLF1, but also the enhancer-specific histone lysine acetyltransferases EP300 and CBP, had the strongest impact on the long-range enhancer-controlled genes (Figures 4C, 4D, and S8).

Unsupervised clustering based on the transcriptional responses of long-range enhancer-controlled genes showed that factors belonging to the same protein complex (cohesin, mediator, or the basal transcription machinery) grouped together, confirming that subunits of the same protein complex shared the same target genes (Figure 4E). As seen before for MED14 and LDB1, the long-range target genes of GATA1, GFI1B, KLF1, EP300, CREBBP, and WDR5, and the mediator subunits generally also required cohesin for normal expression (Figure 4F).

Collectively, this demonstrated that long-range enhancer-controlled genes are exquisitely sensitive to protein-level perturbations, not only of cohesin but also of many of the factors that are recruited to enhancers to support their activity.

Long-range genes are the first to respond to low doses of JQ1 BET inhibitor

Finally, this led us to investigate if long-range genes were perhaps also preferred targets of JQ1, a BET (bromodomain and extraterminal domain) bromodomain inhibitor implicated in (super)enhancer regulation and general transcription.[53,54] JQ1 and other BET inhibitors are used in the clinic as anti-cancer drugs, for example, for hematopoietic cancers, as they repress expression of oncogenes like *MYC*. We used a published dataset that employed SLAM-seq in K562 cells after 30-min exposure to different doses of JQ1.[53] At high JQ1 doses (1–5 μM), transcription was found globally reduced, but at low doses (200 nM), there was more selective gene repression. Here, we intersected the gene expression effects after treatment with 200 nM, 1 μM, and 5 μM JQ1, with our categories of differently connected genes. At high doses (1–5 μM), all categories of genes were similarly affected, but at the low dose of JQ1 (200 nM), mid- and long-range controlled genes were found to be hypersensitive to JQ1 (Figures 4G–4I). Stratification for typical enhancer features showed that the overall higher activity of distal enhancers likely contributes to but does not fully explain the exquisite sensitivity of their connected genes to acute JQ1 treatment (Figure S9A).

DISCUSSION

Here, we dissect the contribution of genomic distance to enhancer-mediated gene activation, using CRISPRi screens, targeted factor perturbations, and an E2G-based analysis of transcriptomes. We find that regulatory factors differentially affect enhancer-driven gene expression in a distance-dependent manner (Figure 5). To elaborate on our cohesin finding, we find that reductions in core cohesin subunits, as well as auxiliary factors, specifically drive the downregulation of genes controlled by distal enhancers, while upregulating genes with proximal enhancers. This confirms and substantiates recent observations[32,33,55–57] on a genome-wide scale. We find that particularly EP300-high long-range enhancers require cohesin to act over distance. For short-range enhancers, EP300 levels are not predictive for cohesin sensitivity. Enhancers have been proposed to serve as chromatin entry sites for cohesin.[32,58–61] Assuming this is particularly true for the EP300-high enhancers, also classified as super-enhancers, it may explain their increased dependence on cohesin when acting over a distance.[21] Whether EP300-low enhancers can act over distance in a cohesin-independent manner, whether they also need cohesin but perhaps are more resistant to fluctuating cohesin levels, or whether they are in fact not bona fide long-range enhancers predicted by the E2G model remains to be investigated. At the *HBB* locus, we find that cohesin levels determine the outcome of the competition between the distal and more proximal *HBB*-like genes for activation by their shared super-enhancer, akin to observations made at the protocadherin locus.[62,63] It also shows that cohesin does not influence the intrinsic strength of an enhancer per se; it increases its capacity to act over distance.

A striking observation was that for many factors supporting enhancer activity, we nearly always found that their loss was foremost sensed by long-range controlled genes. For the mediator complex, this was most appreciable for MED16, MED23, and MED24, subunits of the Tail module, and for CCNC and MED12, both part of the CDK/kinase module (Figures 1D, 1E, 4C, and 4D).[4] A recent study found that these modules are indeed necessary for a much smaller set of target genes than the other, more central modules of mediator.[48] In agreement, studies in yeast have shown that the central subunits of mediator directly associate with gene promoters and are more ubiquitously required for gene activity, while Tail subunits are more selectively associated with UAS-based gene activation.[64] Our data are consistent with a more restricted, enhancer-centered function of the Tail and CDK/kinase modules of mediator and show that fluctuations in mediator protein levels are first sensed by genes controlled by linearly separated enhancers that are located at tens to hundreds of kilobases from the gene.

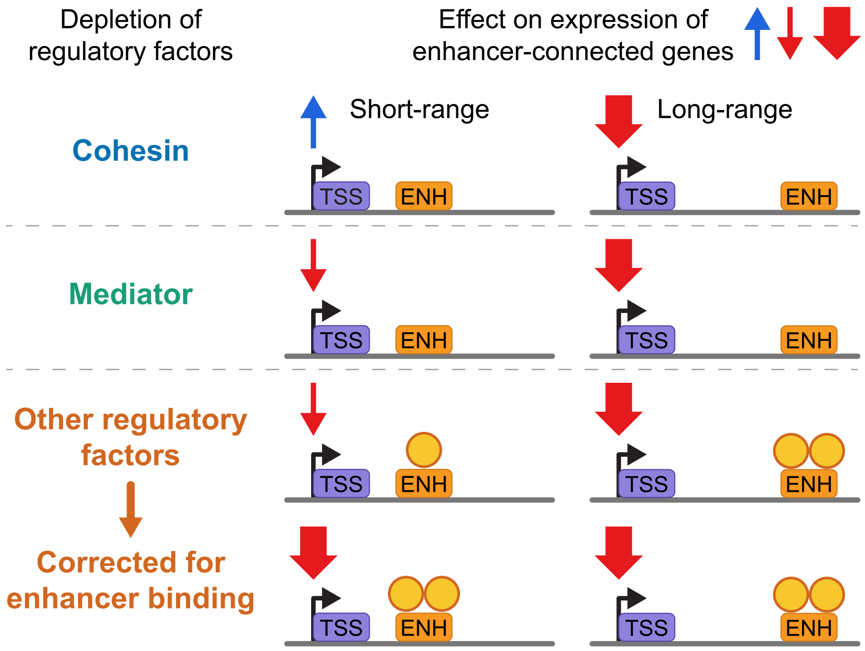

Figure 5. Summary of key results
Cohesin regulates genes connected to enhancers in a distance-specific manner, being necessary for long-range activation but hindering short-range enhancer function. Mediator generally affects gene expression but displays a bias for long-range connected genes. This is also the case for other factors like LDB1, where this is due to long-range enhancers binding higher levels of such regulatory proteins.

Increased enhancer distance is known to generally result in decreased transcriptional output.[24,32] It has also been shown that the exact effect of linear separation relates to enhancer strength[24,65] and promoter type.[66,67] Our work now further shows that at larger distances from the gene promoter, enhancers become more dependent on an optimal *trans*-acting environment for their *cis*-regulatory function. This conclusion is in line with observations recently made in the *PITX1* locus during mouse development[68] and with a previous study in which we showed that a reporter gene inserted in a repressive chromatin environment was better protected against silencing by proximally than by distally inserted enhancers.[32] We propose that modulating distances between *cis*-regulatory DNA elements like enhancers and promoters is a principle by which evolution can limit gene expression to subsets of (highly specialized) cells. Our work therefore also implies that the long stretches of intrinsically non-regulatory DNA in our genome do have a regulatory function—by altering the linear separation, they can influence the responsiveness of genes to regulatory DNA elements like enhancers. Indeed, in a parallel study we find that forced linear recruitment of an enhancer, by the deletion of large intervening stretches of non-regulatory DNA sequences, results in reactivation of developmentally silenced fetal globin genes in adult cells. Apparently, the linear separation of the enhancer is a necessity for these genes to establish and maintain silencing during development.[69]

Why would long-range enhancer-controlled genes be more sensitive to fluctuating protein levels than other genes, including short-range enhancer-controlled genes? One reason could be that they are more often located in gene deserts, where they will be less densely surrounded by other supporting active regulatory DNA elements and more often surrounded by repressive chromatin. Clustering of enhancers on chromosomes can support their individual activity and helps protect them against silencing.[70,71] Another possibility is that long-range controlled genes more often rely on strong enhancers. As implicit to the ABC model and also intuitive, weaker enhancers can only impact expression when they are in sufficient close linear proximity to frequently contact the gene.[45] Assuming that strong enhancers depend more on the cooperative binding and action of multiple TFs than weak enhancers, it stands to reason that perturbations in any of these factors will affect larger numbers of long-range than short-range controlled genes. This principle was exemplified by LDB1, whose depletion had more impact on long-range controlled genes, not because LDB1-bound proximal enhancers were more resistant to LDB1 depletion, but simply because long-range enhancers recruit more LDB1 than do short-range enhancers.

We predict that the notion that long-range enhancer-controlled genes are most vulnerable to reduced levels of enhancer biology proteins will also be relevant for disease. Diseases where cohesin factors are mutated (Cornelia de Lange syndrome [CdlS]) are associated with misexpression of developmentally important genes. Interestingly, mutations in BRD4, a target of JQ1, have been linked to cohesin and this type of disease,[72–75] which is also the case for EP300.[76,77] This raises the interesting possibility that at least part of the cohesin-driven CdlS etiology is mediated via strong, long-range, cell-type-specific enhancers.[55]

As the coordination of cell differentiation and proliferation is essential for cancer development, it follows that misregulation of long-range gene expression also impacts this balance. Indeed, studies in hematopoietic stem cells and associated cancers have shown that cohesin depletion or STAG2 mutations impair differentiation-associated gene expression and promote a self-renewal state,[78,79] while the induction of signaling, often mediated by distal enhancers, can also have a strong dependence on cohesin.[80–84] We speculate that disrupted cell differentiation and induced cell proliferation because of the inappropriate activation of long-range controlled differentiation genes may also be part of the explanation why critical enhancer-associated factors like LDB1,[85,86] EP300,[87,88] mediator,[89,90] and others may be linked to cancer.

An example of the convergence of distal enhancers and cancer is the *MYC* oncogene. *MYC* is ubiquitously expressed but relies for its expression in different cell types on cell-type-specific distal enhancers, indeed, partially dependent on cohesin and three-dimensional (3D) genome.[91–94] Overexpressed in more than 50% of all human cancers and in 70% of the hematopoietic cancers, *MYC* is therefore the prime target of cancer therapeutic agents like JQ1.[95] JQ1 targets BET bromodomain proteins like BRD4, but it is unclear why this preferentially targets the *MYC* oncogene. Our data suggest this may at least partially be explained by the fact that *MYC* is controlled by long-range enhancers, making the gene more sensitive for disrupted enhancer function. Indeed, *MYC* expression was found hypersensitive to acute depletion of RAD21, LDB1, MED14, and MED23 (Figure S9B), and to the knockdown of many other factors, including GATA1, SMC1A, and mediator subunits (Figure S9C). Possibly, therefore, there may be more proteins that can serve as targets for anti-cancer medication.

Limitations of the study

In the CRISPRi screen, we might have missed distance-specific phenotypes, due to using a targeted library, imperfect knockdown, or dilution of distance dependence due to indirect effects.

We focused our E2G-based analysis on genes contacting at least one strong enhancer within a certain distance. However, many genes are in contact with enhancers with various strengths at different distances, which also might be bound by different proteins. How these genes respond to perturbation of a specific factor, and how enhancers at different distances cooperate or buffer one another, requires further study.

The genes in the different enhancer-distance groups also vary in other features of their enhancers and promoters. Although we aimed to control for this, it remains difficult to fully dissect, and more experiments where E-P distance is the only variable are necessary.

We performed 4C-seq on a selection of long-range enhancer-controlled genes with strong phenotypes after RAD21 and LDB1 depletion. To determine how these factors and mediator more generally connect 3D chromosome organization with transcription, more (genome-wide) high-resolution chromosome conformation capture (3C)-based experiments would be necessary.

RESOURCE AVAILABILITY

Lead contact

Further information and requests should be directed to the lead contact, Wouter de Laat (w.l.delaat@umcutrecht.nl).

Materials availability

Plasmids and cell lines used in this study are available upon request to the lead contact.

Data and code availability

- CRISPRi screen, BrU-seq, RNA-seq, and 4C-seq data generated in this study were deposited to GEO under the following accession numbers: CRISPRi screen (GEO: GSE281575), BrU-seq (GEO: GSE281753), RNA-seq (GEO: GSE281752 and GSE280672), and 4C-seq (GEO: GSE281419, GSE281420, and GSE281576).
- Previously published data: ENCODE-rE2G predictions, K562 (ENCODE: ENCFF202FID), MED14-AID SLAM-seq (GEO: GSE198944), JQ1 SLAM-seq (GEO: GSE100708), and LDB1 ChIP-seq (GEO: GSE142228).
- The code to analyze sequencing data has been deposited to Zenodo (https://doi.org/10.5281/zenodo.14731771).
- Raw western blot images of acute degradation of RAD21, MED23, or LDB1 were uploaded to Mendeley Data (https://doi.org/10.17632/r9878ks8nm.1).

ACKNOWLEDGMENTS

We thank De Laat lab members for useful discussions and the Hubrecht Flow Cytometry facility for help with the fluorescence-activated cell sorting (FACS)-based experiments. We thank the Engreitz lab for generating the ENCODE-rE2G model and Jesse Engreitz for referring to this dataset. This work is part of the Oncode Institute. We acknowledge the Utrecht Sequencing Facility (USEQ) for providing sequencing service and data. USEQ is subsidized by the University Medical Center Utrecht and The Netherlands X-omics Initiative (Netherlands Organisation for Scientific Research [NWO] project 184.034.019). The research in the laboratory of W.d.L. was financially supported by an NWO Groot grant (2019.012) from the NWO, Oncode Institute base funding to W.d.L., and ZonMW Enabling Technologies Hotel, grant no. 435005023 (to W.d.L. and Single Cell Discoveries).

AUTHOR CONTRIBUTIONS

S.J.D.T. and N.J.R. generated the dCas9-KRAB cell lines, performed and analyzed the CRISPRi screens, and performed single-guide CRISPRi validations. S.J.D.T., M.J.A.M.V., and M.J.R. generated the degron cell lines and performed the depletion experiments. S.J.D.T. performed the BrU-seq experiments. M.J.A.M.V. performed the 4C-seq experiments. S.J.D.T., N.J.R., and M.J.M. performed the total RNA-seq experiments. P.H.L.K. performed the screen, BrU-seq, total RNA-seq, and 4C-seq analyses. R.A.G. performed the ChIP-seq and RNA-seq analyses. S.J.D.T., A.N.-A., and A.A. performed the ABC-based analyses. S.J.D.T., N.J.R., P.H.L.K., and W.d.L. conceived the study and designed the experiments. S.J.D.T. and W.d.L. wrote the manuscript, with input from all authors.

DECLARATION OF INTERESTS

M.J.M. is the co-founder of Single Cell Discoveries, a commercial transcriptomics service provider.

STAR★METHODS

Detailed methods are provided in the online version of this paper and include the following:

- KEY RESOURCES TABLE
- EXPERIMENTAL MODEL AND STUDY PARTICIPANT DETAILS
- METHOD DETAILS
  - CRISPRi sgRNA library cloning
  - CRISPRi screen
  - Single-guide CRISPRi knock down
  - mAID tagging of RAD21
  - FKBP tagging of MED23 and LDB1
  - Protein depletion
  - Western blot
  - Reporter flow cytometry after acute depletion
  - 4C-seq
  - Nascent RNA-sequencing (BrU-seq)
  - Poly(A) RNA-sequencing
  - High-throughput bulk RNA-sequencing
  - Analysis of CRISPRi screen
  - Analysis of 4C-seq
  - Nascent RNA-sequencing (BrU-seq) analysis
  - Poly(A) RNA-sequencing analysis
  - High-throughput bulk RNA-sequencing analysis

- ○ E2G-based analysis
- ○ Published SLAM-seq data
- ○ ChIP-seq analysis
- QUANTIFICATION AND STATISTICAL ANALYSIS

SUPPLEMENTAL INFORMATION

Supplemental information can be found online at https://doi.org/10.1016/j.xgen.2025.100778.

Received: October 31, 2024
Revised: December 18, 2024
Accepted: January 29, 2025
Published: February 25, 2025

REFERENCES

1. Kim, S., and Wysocka, J. (2023). Deciphering the multi-scale, quantitative cis-regulatory code. Mol. Cell *83*, 373–392. https://doi.org/10.1016/j.molcel.2022.12.032.
2. Yang, J.H., and Hansen, A.S. (2024). Enhancer selectivity in space and time: from enhancer-promoter interactions to promoter activation. Nat. Rev. Mol. Cell Biol. *25*, 574–591. https://doi.org/10.1038/s41580-024-00710-6.
3. Malik, S., and Roeder, R.G. (2023). Regulation of the RNA polymerase II pre-initiation complex by its associated coactivators. Nat. Rev. Genet. *24*, 767–782. https://doi.org/10.1038/s41576-023-00630-9.
4. Richter, W.F., Nayak, S., Iwasa, J., and Taatjes, D.J. (2022). The Mediator complex as a master regulator of transcription by RNA polymerase II. Nat. Rev. Mol. Cell Biol. *23*, 732–749. https://doi.org/10.1038/s41580-022-00498-3.
5. Lettice, L.A., Heaney, S.J.H., Purdie, L.A., Li, L., de Beer, P., Oostra, B.A., Goode, D., Elgar, G., Hill, R.E., and de Graaff, E. (2003). A long-range Shh enhancer regulates expression in the developing limb and fin and is associated with preaxial polydactyly. Hum. Mol. Genet. *12*, 1725–1735. https://doi.org/10.1093/hmg/ddg180.
6. Bush, M., and Dixon, R. (2012). The role of bacterial enhancer binding proteins as specialized activators of sigma54-dependent transcription. Microbiol. Mol. Biol. Rev. *76*, 497–529. https://doi.org/10.1128/MMBR.00006-12.
7. Wedel, A., Weiss, D.S., Popham, D., Dröge, P., and Kustu, S. (1990). A bacterial enhancer functions to tether a transcriptional activator near a promoter. Science *248*, 486–490. https://doi.org/10.1126/science.1970441.
8. Rossi, M.J., Kuntala, P.K., Lai, W.K.M., Yamada, N., Badjatia, N., Mittal, C., Kuzu, G., Bocklund, K., Farrell, N.P., Blanda, T.R., et al. (2021). A high-resolution protein architecture of the budding yeast genome. Nature *592*, 309–314. https://doi.org/10.1038/s41586-021-03314-8.
9. Chen, Z., Snetkova, V., Bower, G., Jacinto, S., Clock, B., Dizehchi, A., Barozzi, I., Mannion, B.J., Alcaina-Caro, A., Lopez-Rios, J., et al. (2024). Increased enhancer-promoter interactions during developmental enhancer activation in mammals. Nat. Genet. *56*, 675–685. https://doi.org/10.1038/s41588-024-01681-2.
10. Gschwind, A.R., Mualim, K.S., Karbalayghareh, A., Sheth, M.U., Dey, K.K., Jagoda, E., Nurtdinov, R.N., Xi, W., Tan, A.S., Jones, H., et al. (2023). An encyclopedia of enhancer-gene regulatory interactions in the human genome. Preprint at bioRxiv. https://doi.org/10.1101/2023.11.09.563812.
11. Ghavi-Helm, Y., Klein, F.A., Pakozdi, T., Ciglar, L., Noordermeer, D., Huber, W., and Furlong, E.E.M. (2014). Enhancer loops appear stable during development and are associated with paused polymerase. Nature *512*, 96–100. https://doi.org/10.1038/nature13417.
12. Tolhuis, B., Palstra, R.J., Splinter, E., Grosveld, F., and de Laat, W. (2002). Looping and interaction between hypersensitive sites in the active beta-globin locus. Mol. Cell *10*, 1453–1465.
13. Davidson, I.F., Bauer, B., Goetz, D., Tang, W., Wutz, G., and Peters, J.M. (2019). DNA loop extrusion by human cohesin. Science *366*, 1338–1345. https://doi.org/10.1126/science.aaz3418.
14. de Wit, E., Vos, E.S.M., Holwerda, S.J.B., Valdes-Quezada, C., Verstegen, M.J.A.M., Teunissen, H., Splinter, E., Wijchers, P.J., Krijger, P.H.L., and de Laat, W. (2015). CTCF Binding Polarity Determines Chromatin Looping. Mol. Cell *60*, 676–684. https://doi.org/10.1016/j.molcel.2015.09.023.
15. Davidson, I.F., Barth, R., Zaczek, M., van der Torre, J., Tang, W., Nagasaka, K., Janissen, R., Kerssemakers, J., Wutz, G., Dekker, C., and Peters, J.M. (2023). CTCF is a DNA-tension-dependent barrier to cohesin-mediated loop extrusion. Nature *616*, 822–827. https://doi.org/10.1038/s41586-023-05961-5.
16. Dixon, J.R., Selvaraj, S., Yue, F., Kim, A., Li, Y., Shen, Y., Hu, M., Liu, J.S., and Ren, B. (2012). Topological domains in mammalian genomes identified by analysis of chromatin interactions. Nature *485*, 376–380. https://doi.org/10.1038/nature11082.
17. Nora, E.P., Lajoie, B.R., Schulz, E.G., Giorgetti, L., Okamoto, I., Servant, N., Piolot, T., van Berkum, N.L., Meisig, J., Sedat, J., et al. (2012). Spatial partitioning of the regulatory landscape of the X-inactivation centre. Nature *485*, 381–385. https://doi.org/10.1038/nature11049.
18. Szabo, Q., Donjon, A., Jerković, I., Papadopoulos, G.L., Cheutin, T., Bonev, B., Nora, E.P., Bruneau, B.G., Bantignies, F., and Cavalli, G. (2020). Regulation of single-cell genome organization into TADs and chromatin nanodomains. Nat. Genet. *52*, 1151–1157. https://doi.org/10.1038/s41588-020-00716-8.
19. Gabriele, M., Brandão, H.B., Grosse-Holz, S., Jha, A., Dailey, G.M., Cattoglio, C., Hsieh, T.H.S., Mirny, L., Zechner, C., and Hansen, A.S. (2022). Dynamics of CTCF- and cohesin-mediated chromatin looping revealed by live-cell imaging. Science *376*, 496–501. https://doi.org/10.1126/science.abn6583.
20. Mach, P., Kos, P.I., Zhan, Y., Cramard, J., Gaudin, S., Tünnermann, J., Marchi, E., Eglinger, J., Zuin, J., Kryzhanovska, M., et al. (2022). Cohesin and CTCF control the dynamics of chromosome folding. Nat. Genet. *54*, 1907–1918. https://doi.org/10.1038/s41588-022-01232-7.
21. Rao, S.S.P., Huang, S.C., Glenn St Hilaire, B., Engreitz, J.M., Perez, E.M., Kieffer-Kwon, K.R., Sanborn, A.L., Johnstone, S.E., Bascom, G.D., Bochkov, I.D., et al. (2017). Cohesin Loss Eliminates All Loop Domains. Cell *171*, 305–320. https://doi.org/10.1016/j.cell.2017.09.026.
22. Liu, Y., and Dekker, J. (2022). CTCF-CTCF loops and intra-TAD interactions show differential dependence on cohesin ring integrity. Nat. Cell Biol. *24*, 1516–1527. https://doi.org/10.1038/s41556-022-00992-y.
23. Hsieh, T.H.S., Cattoglio, C., Slobodyanyuk, E., Hansen, A.S., Darzacq, X., and Tjian, R. (2022). Enhancer-promoter interactions and transcription are largely maintained upon acute loss of CTCF, cohesin, WAPL or YY1. Nat. Genet. *54*, 1919–1932. https://doi.org/10.1038/s41588-022-01223-8.
24. Zuin, J., Roth, G., Zhan, Y., Cramard, J., Redolfi, J., Piskadlo, E., Mach, P., Kryzhanovska, M., Tihanyi, G., Kohler, H., et al. (2022). Nonlinear control of transcription through enhancer-promoter interactions. Nature *604*, 571–577. https://doi.org/10.1038/s41586-022-04570-y.
25. Flavahan, W.A., Drier, Y., Liau, B.B., Gillespie, S.M., Venteicher, A.S., Stemmer-Rachamimov, A.O., Suvà, M.L., and Bernstein, B.E. (2016). Insulator dysfunction and oncogene activation in IDH mutant gliomas. Nature *529*, 110–114. https://doi.org/10.1038/nature16490.
26. Vos, E.S.M., Valdes-Quezada, C., Huang, Y., Allahyar, A., Verstegen, M.J.A.M., Felder, A.-K., van der Vegt, F., Uijttewaal, E.C.H., Krijger, P.H.L., and de Laat, W. (2021). Interplay between CTCF boundaries and a super enhancer controls cohesin extrusion trajectories and gene expression. Mol. Cell *81*, 3082–3095. https://doi.org/10.1016/j.molcel.2021.06.008.

27. Guo, C., Yoon, H.S., Franklin, A., Jain, S., Ebert, A., Cheng, H.L., Hansen, E., Despo, O., Bossen, C., Vettermann, C., et al. (2011). CTCF-binding elements mediate control of V(D)J recombination. Nature *477*, 424–430. https://doi.org/10.1038/nature10495.

28. Rekaik, H., Lopez-Delisle, L., Hintermann, A., Mascrez, B., Bochaton, C., Mayran, A., and Duboule, D. (2023). Sequential and directional insulation by conserved CTCF sites underlies the Hox timer in stembryos. Nat. Genet. *55*, 1164–1175. https://doi.org/10.1038/s41588-023-01426-7.

29. Hnisz, D., Weintraub, A.S., Day, D.S., Valton, A.L., Bak, R.O., Li, C.H., Goldmann, J., Lajoie, B.R., Fan, Z.P., Sigova, A.A., et al. (2016). Activation of proto-oncogenes by disruption of chromosome neighborhoods. Science *351*, 1454–1458. https://doi.org/10.1126/science.aad9024.

30. Lupianez, D.G., Kraft, K., Heinrich, V., Krawitz, P., Brancati, F., Klopocki, E., Horn, D., Kayserili, H., Opitz, J.M., Laxova, R., et al. (2015). Disruptions of topological chromatin domains cause pathogenic rewiring of gene-enhancer interactions. Cell *161*, 1012–1025. https://doi.org/10.1016/j.cell.2015.04.004.

31. Nora, E.P., Goloborodko, A., Valton, A.L., Gibcus, J.H., Uebersohn, A., Abdennur, N., Dekker, J., Mirny, L.A., and Bruneau, B.G. (2017). Targeted Degradation of CTCF Decouples Local Insulation of Chromosome Domains from Genomic Compartmentalization. Cell *169*, 930–944. https://doi.org/10.1016/j.cell.2017.05.004.

32. Rinzema, N.J., Sofiadis, K., Tjalsma, S.J.D., Verstegen, M.J.A.M., Oz, Y., Valdes-Quezada, C., Felder, A.K., Filipovska, T., van der Elst, S., de Andrade Dos Ramos, Z., et al. (2022). Building regulatory landscapes reveals that an enhancer can recruit cohesin to create contact domains, engage CTCF sites and activate distant genes. Nat. Struct. Mol. Biol. *29*, 563–574. https://doi.org/10.1038/s41594-022-00787-7.

33. Kane, L., Williamson, I., Flyamer, I.M., Kumar, Y., Hill, R.E., Lettice, L.A., and Bickmore, W.A. (2022). Cohesin is required for long-range enhancer action at the Shh locus. Nat. Struct. Mol. Biol. *29*, 891–897. https://doi.org/10.1038/s41594-022-00821-8.

34. Spiluttini, B., Gu, B., Belagal, P., Smirnova, A.S., Nguyen, V.T., Hébert, C., Schmidt, U., Bertrand, E., Darzacq, X., and Bensaude, O. (2010). Splicing-independent recruitment of U1 snRNP to a transcription unit in living cells. J. Cell Sci. *123*, 2085–2093. https://doi.org/10.1242/jcs.061358.

35. Castillo-Guzman, D., Hartono, S.R., Sanz, L.A., and Chédin, F. (2020). SF3B1-targeted Splicing Inhibition Triggers Global Alterations in Transcriptional Dynamics and R-Loop Metabolism. Preprint at bioRxiv. https://doi.org/10.1101/2020.06.08.130583.

36. Caizzi, L., Monteiro-Martins, S., Schwalb, B., Lysakovskaia, K., Schmitzova, J., Sawicka, A., Chen, Y., Lidschreiber, M., and Cramer, P. (2021). Efficient RNA polymerase II pause release requires U2 snRNP function. Mol. Cell *81*, 1920–1934. https://doi.org/10.1016/j.molcel.2021.02.016.

37. Wysocka, J., Swigut, T., Milne, T.A., Dou, Y., Zhang, X., Burlingame, A.L., Roeder, R.G., Brivanlou, A.H., and Allis, C.D. (2005). WDR5 associates with histone H3 methylated at K4 and is essential for H3 K4 methylation and vertebrate development. Cell *121*, 859–872. https://doi.org/10.1016/j.cell.2005.03.036.

38. Wijgerde, M., Gribnau, J., Trimborn, T., Nuez, B., Philipsen, S., Grosveld, F., and Fraser, P. (1996). The role of EKLF in human beta-globin gene competition. Genes Dev. *10*, 2894–2902. https://doi.org/10.1101/gad.10.22.2894.

39. Welch, J.J., Watts, J.A., Vakoc, C.R., Yao, Y., Wang, H., Hardison, R.C., Blobel, G.A., Chodosh, L.A., and Weiss, M.J. (2004). Global regulation of erythroid gene expression by transcription factor GATA-1. Blood *104*, 3136–3147. https://doi.org/10.1182/blood-2004-04-1603.

40. Doyon, Y., Selleck, W., Lane, W.S., Tan, S., and Côté, J. (2004). Structural and functional conservation of the NuA4 histone acetyltransferase complex from yeast to humans. Mol. Cell Biol. *24*, 1884–1896. https://doi.org/10.1128/MCB.24.5.1884-1896.2004.

41. Cheloufi, S., Elling, U., Hopfgartner, B., Jung, Y.L., Murn, J., Ninova, M., Hubmann, M., Badeaux, A.I., Euong Ang, C., Tenen, D., et al. (2015). The histone chaperone CAF-1 safeguards somatic cell identity. Nature *528*, 218–224. https://doi.org/10.1038/nature15749.

42. Volk, A., Liang, K., Suraneni, P., Li, X., Zhao, J., Bulic, M., Marshall, S., Pulakanti, K., Malinge, S., Taub, J., et al. (2018). A CHAF1B-Dependent Molecular Switch in Hematopoiesis and Leukemia Pathogenesis. Cancer Cell *34*, 707–723. https://doi.org/10.1016/j.ccell.2018.10.004.

43. Yesbolatova, A., Saito, Y., Kitamoto, N., Makino-Itou, H., Ajima, R., Nakano, R., Nakaoka, H., Fukui, K., Gamo, K., Tominari, Y., et al. (2020). The auxin-inducible degron 2 technology provides sharp degradation control in yeast, mammalian cells, and mice. Nat. Commun. *11*, 5701. https://doi.org/10.1038/s41467-020-19532-z.

44. Fulco, C.P., Nasser, J., Jones, T.R., Munson, G., Bergman, D.T., Subramanian, V., Grossman, S.R., Anyoha, R., Doughty, B.R., Patwardhan, T.A., et al. (2019). Activity-by-contact model of enhancer-promoter regulation from thousands of CRISPR perturbations. Nat. Genet. *51*, 1664–1669. https://doi.org/10.1038/s41588-019-0538-0.

45. Thomas, H.F., Feng, S., Haslhofer, F., Huber, M., García Gallardo, M., Loubiere, V., Vanina, D., Pitasi, M., Stark, A., and Buecker, C. (2025). Enhancer cooperativity can compensate for loss of activity over large genomic distances. Mol. Cell *85*, 362–375. https://doi.org/10.1016/j.molcel.2024.11.008.

46. Luo, R., Yan, J., Oh, J.W., Xi, W., Shigaki, D., Wong, W., Cho, H.S., Murphy, D., Cutler, R., Rosen, B.P., et al. (2023). Dynamic network-guided CRISPRi screen identifies CTCF-loop-constrained nonlinear enhancer gene regulatory activity during cell state transitions. Nat. Genet. *55*, 1336–1346. https://doi.org/10.1038/s41588-023-01450-7.

47. Jaeger, M.G., Schwalb, B., Mackowiak, S.D., Velychko, T., Hanzl, A., Imrichova, H., Brand, M., Agerer, B., Chorn, S., Nabet, B., et al. (2020). Selective Mediator dependence of cell-type-specifying transcription. Nat. Genet. *52*, 719–727. https://doi.org/10.1038/s41588-020-0635-0.

48. Bell, C.C., Balic, J.J., Talarmain, L., Gillespie, A., Scolamiero, L., Lam, E.Y.N., Ang, C.S., Faulkner, G.J., Gilan, O., and Dawson, M.A. (2024). Comparative cofactor screens show the influence of transactivation domains and core promoters on the mechanisms of transcription. Nat. Genet. *56*, 1181–1192. https://doi.org/10.1038/s41588-024-01749-z.

49. Guo, X., Plank-Bazinet, J., Krivega, I., Dale, R.K., and Dean, A. (2020). Embryonic erythropoiesis and hemoglobin switching require transcriptional repressor ETO2 to modulate chromatin organization. Nucleic Acids Res. *48*, 10226–10240. https://doi.org/10.1093/nar/gkaa736.

50. Deng, W., Rupon, J.W., Krivega, I., Breda, L., Motta, I., Jahn, K.S., Reik, A., Gregory, P.D., Rivella, S., Dean, A., and Blobel, G.A. (2014). Reactivation of developmentally silenced globin genes by forced chromatin looping. Cell *158*, 849–860. https://doi.org/10.1016/j.cell.2014.05.050.

51. Song, S.H., Hou, C., and Dean, A. (2007). A positive role for NLI/Ldb1 in long-range beta-globin locus control region function. Mol. Cell *28*, 810–822. https://doi.org/10.1016/j.molcel.2007.09.025.

52. Aboreden, N.G., Lam, J.C., Goel, V.Y., Wang, S., Wang, X., Midla, S.C., Quijano, A., Keller, C.A., Giardine, B.M., Hardison, R.C., et al. (2025). LDB1 establishes multi-enhancer networks to regulate gene expression. Mol. Cell *85*, 376–393. https://doi.org/10.1016/j.molcel.2024.11.037.

53. Muhar, M., Ebert, A., Neumann, T., Umkehrer, C., Jude, J., Wieshofer, C., Rescheneder, P., Lipp, J.J., Herzog, V.A., Reichholf, B., et al. (2018). SLAM-seq defines direct gene-regulatory functions of the BRD4-MYC axis. Science *360*, 800–805. https://doi.org/10.1126/science.aao2793.

54. Filippakopoulos, P., Qi, J., Picaud, S., Shen, Y., Smith, W.B., Fedorov, O., Morse, E.M., Keates, T., Hickman, T.T., Felletar, I., et al. (2010). Selective inhibition of BET bromodomains. Nature *468*, 1067–1073. https://doi.org/10.1038/nature09504.

55. Calderon, L., Weiss, F.D., Beagan, J.A., Oliveira, M.S., Georgieva, R., Wang, Y.F., Carroll, T.S., Dharmalingam, G., Gong, W., Tossell, K., et al. (2022). Cohesin-dependence of neuronal gene expression relates to chromatin loop length. Elife *11*, e76539. https://doi.org/10.7554/eLife.76539.

56. Guckelberger, P., Doughty, B.R., Munson, G., Rao, S.S.P., Tan, Y., Cai, X.S., Fulco, C.P., Nasser, J., Mualim, K.S., Bergman, D.T., et al. (2024). Cohesin-mediated 3D contacts tune enhancer-promoter regulation. Preprint at bioRxiv. https://doi.org/10.1101/2024.07.12.603288.

57. Hansen, K.L., Adachi, A.S., Braccioli, L., Kadvani, S., Boileau, R.M., Pokorny, B., Shah, R., Anderson, E.C., Martinovic, M., Zhang, K., et al. (2024). Synergy between cis-regulatory elements can render cohesin dispensable for distal enhancer function. Preprint at bioRxiv. https://doi.org/10.1101/2024.10.04.615095.

58. Kim, J., Wang, H., and Ercan, S. (2024). Cohesin organizes 3D DNA contacts surrounding active enhancers in C. elegans. Preprint at bioRxiv. https://doi.org/10.1101/2023.09.18.558239.

59. Liu, N.Q., Maresca, M., van den Brand, T., Braccioli, L., Schijns, M.M.G.A., Teunissen, H., Bruneau, B.G., Nora, E.P., and de Wit, E. (2021). WAPL maintains a cohesin loading cycle to preserve cell-type-specific distal gene regulation. Nat. Genet. *53*, 100–109. https://doi.org/10.1038/s41588-020-00744-4.

60. Vian, L., Pękowska, A., Rao, S.S.P., Kieffer-Kwon, K.R., Jung, S., Baranello, L., Huang, S.C., El Khattabi, L., Dose, M., Pruett, N., et al. (2018). The Energetics and Physiological Impact of Cohesin Extrusion. Cell *175*, 292–294. https://doi.org/10.1016/j.cell.2018.09.002.

61. Guo, Y., Al-Jibury, E., Garcia-Millan, R., Ntagiantas, K., King, J.W.D., Nash, A.J., Galjart, N., Lenhard, B., Rueckert, D., Fisher, A.G., et al. (2022). Chromatin jets define the properties of cohesin-driven in vivo loop extrusion. Mol. Cell *82*, 3769–3780. https://doi.org/10.1016/j.molcel.2022.09.003.

62. Kiefer, L., Chiosso, A., Langen, J., Buckley, A., Gaudin, S., Rajkumar, S.M., Servito, G.I.F., Cha, E.S., Vijay, A., Yeung, A., et al. (2023). WAPL functions as a rheostat of Protocadherin isoform diversity that controls neural wiring. Science *380*, eadf8440. https://doi.org/10.1126/science.adf8440.

63. Kiefer, L., Gaudin, S., Rajkumar, S.M., Servito, G.I.F., Langen, J., Mui, M.H., Nawsheen, S., and Canzio, D. (2024). Tuning cohesin trajectories enables differential readout of the Pcdhα cluster across neurons. Science *385*, eadm9802. https://doi.org/10.1126/science.adm9802.

64. Warfield, L., Donczew, R., Mahendrawada, L., and Hahn, S. (2022). Yeast Mediator facilitates transcription initiation at most promoters via a Tail-independent mechanism. Mol. Cell *82*, 4033–4048. https://doi.org/10.1016/j.molcel.2022.09.016.

65. Thomas, H., Feng, S., Huber, M., Loubiere, V., Vanina, D., Pitasi, M., Stark, A., and Buecker, C. (2023). Enhancer cooperativity can compensate for loss of activity over large genomic distances.570399 https://doi.org/10.1101/2023.12.06.570399

66. Bergman, D.T., Jones, T.R., Liu, V., Ray, J., Jagoda, E., Siraj, L., Kang, H.Y., Nasser, J., Kane, M., Rios, A., et al. (2022). Compatibility rules of human enhancer and promoter sequences. Nature *607*, 176–184. https://doi.org/10.1038/s41586-022-04877-w.

67. Martinez-Ara, M., Comoglio, F., van Arensbergen, J., and van Steensel, B. (2022). Systematic analysis of intrinsic enhancer-promoter compatibility in the mouse genome. Mol. Cell *82*, 2519–2531. https://doi.org/10.1016/j.molcel.2022.04.009.

68. Bompadre, O., Rouco, R., Darbellay, F., Rauseo, A., Guerard-Millet, F., Gentile, C., Kmita, M., and Andrey, G. (2024). Liebenberg syndrome severity arises from variations in *Pitx1* locus topology and ectopically transcribing cells. Preprint at bioRxiv. https://doi.org/10.1101/2024.03.04.582675.

69. Felder, A.-K., Tjalsma, S.J., Verhagen, H.J., Majied, R., Verstegen, M.J., Verheul, T.C., Mohnani, R., Gremmen, R., Krijger, P.H., Philipsen, S., et al. (2025). Reactivation of developmentally silenced globin genes through genomic deletions reveals that enhancer distance matters. Preprint at bioRxiv. https://doi.org/10.1101/2025.01.13.632719.

70. Blayney, J.W., Francis, H., Rampasekova, A., Camellato, B., Mitchell, L., Stolper, R., Cornell, L., Babbs, C., Boeke, J.D., Higgs, D.R., and Kassouf, M. (2023). Super-enhancers include classical enhancers and facilitators to fully activate gene expression. Cell *186*, 5826–5839. https://doi.org/10.1016/j.cell.2023.11.030.

71. Grosveld, F., van Assendelft, G.B., Greaves, D.R., and Kollias, G. (1987). Position-independent, high-level expression of the human beta-globin gene in transgenic mice. Cell *51*, 975–985. https://doi.org/10.1016/0092-8674(87)90584-8.

72. Linares-Saldana, R., Kim, W., Bolar, N.A., Zhang, H., Koch-Bojalad, B.A., Yoon, S., Shah, P.P., Karnay, A., Park, D.S., Luppino, J.M., et al. (2021). BRD4 orchestrates genome folding to promote neural crest differentiation. Nat. Genet. *53*, 1480–1492. https://doi.org/10.1038/s41588-021-00934-8.

73. Olley, G., Ansari, M., Bengani, H., Grimes, G.R., Rhodes, J., von Kriegsheim, A., Blatnik, A., Stewart, F.J., Wakeling, E., Carroll, N., et al. (2018). BRD4 interacts with NIPBL and BRD4 is mutated in a Cornelia de Lange–like syndrome. Nat. Genet. *50*, 329–332. https://doi.org/10.1038/s41588-018-0042-y.

74. Alesi, V., Dentici, M.L., Loddo, S., Genovese, S., Orlando, V., Calacci, C., Pompili, D., Dallapiccola, B., Digilio, M.C., and Novelli, A. (2019). Confirmation of BRD4 haploinsufficiency role in Cornelia de Lange–like phenotype and delineation of a 19p13.12p13.11 gene contiguous syndrome. Ann. Hum. Genet. *83*, 100–109. https://doi.org/10.1111/ahg.12289.

75. Weiss, F.D., Calderon, L., Wang, Y.F., Georgieva, R., Guo, Y., Cvetesic, N., Kaur, M., Dharmalingam, G., Krantz, I.D., Lenhard, B., et al. (2021). Neuronal genes deregulated in Cornelia de Lange Syndrome respond to removal and re-expression of cohesin. Nat. Commun. *12*, 2919. https://doi.org/10.1038/s41467-021-23141-9.

76. Cucco, F., Sarogni, P., Rossato, S., Alpa, M., Patimo, A., Latorre, A., Magnani, C., Puisac, B., Ramos, F.J., Pié, J., and Musio, A. (2020). Pathogenic variants in EP300 and ANKRD11 in patients with phenotypes overlapping Cornelia de Lange syndrome. Am. J. Med. Genet. *182*, 1690–1696. https://doi.org/10.1002/ajmg.a.61611.

77. Woods, S.A., Robinson, H.B., Kohler, L.J., Agamanolis, D., Sterbenz, G., and Khalifa, M. (2014). Exome sequencing identifies a novel EP300 frame shift mutation in a patient with features that overlap Cornelia de Lange syndrome. Am. J. Med. Genet. *164A*, 251–258. https://doi.org/10.1002/ajmg.a.36237.

78. Fischer, A., Hernández-Rodríguez, B., Mulet-Lazaro, R., Nuetzel, M., Hölzl, F., van Herk, S., Kavelaars, F.G., Stanewsky, H., Ackermann, U., Niang, A.H., et al. (2024). STAG2 mutations reshape the cohesin-structured spatial chromatin architecture to drive gene regulation in acute myeloid leukemia. Cell Rep. *43*, 114498. https://doi.org/10.1016/j.celrep.2024.114498.

79. Sasca, D., Yun, H., Giotopoulos, G., Szybinski, J., Evan, T., Wilson, N.K., Gerstung, M., Gallipoli, P., Green, A.R., Hills, R., et al. (2019). Cohesin-dependent regulation of gene expression during differentiation is lost in cohesin-mutated myeloid malignancies. Blood *134*, 2195–2208. https://doi.org/10.1182/blood.2019001553.

80. Rinaldi, L., Fettweis, G., Kim, S., Garcia, D.A., Fujiwara, S., Johnson, T.A., Tettey, T.T., Ozbun, L., Pegoraro, G., Puglia, M., et al. (2022). The glucocorticoid receptor associates with the cohesin loader NIPBL to promote long-range gene regulation. Sci. Adv. *8*, eabj8360. https://doi.org/10.1126/sciadv.abj8360.

81. Mayayo-Peralta, I., Gregoricchio, S., Schuurman, K., Yavuz, S., Zaalberg, A., Kojic, A., Abbott, N., Geverts, B., Beerthuijzen, S., Siefert, J., et al. (2023). PAXIP1 and STAG2 converge to maintain 3D genome architecture and facilitate promoter/enhancer contacts to enable stress hormone-dependent transcription. Nucleic Acids Res. *51*, 9576–9593. https://doi.org/10.1093/nar/gkad267.

82. Cuartero, S., Weiss, F.D., Dharmalingam, G., Guo, Y., Ing-Simmons, E., Masella, S., Robles-Rebollo, I., Xiao, X., Wang, Y.F., Barozzi, I., et al. (2018). Control of inducible gene expression links cohesin to hematopoietic progenitor self-renewal and differentiation. Nat. Immunol. *19*, 932–941. https://doi.org/10.1038/s41590-018-0184-1.

83. Siwek, W., Tehrani, S.S.H., Mata, J.F., and Jansen, L.E.T. (2020). Activation of Clustered IFNgamma Target Genes Drives Cohesin-Controlled Transcriptional Memory. Mol. Cell *80*, 396–409. https://doi.org/10.1016/j.molcel.2020.10.005.

84. Serra, F., Nieto-Aliseda, A., Fanlo-Escudero, L., Rovirosa, L., Cabrera-Pasadas, M., Lazarenkov, A., Urmeneta, B., Alcalde-Merino, A., Nola, E.M., Okorokov, A.L., et al. (2024). p53 rapidly restructures 3D chromatin organization to trigger a transcriptional response. Nat. Commun. *15*, 2821. https://doi.org/10.1038/s41467-024-46666-1.

85. Garcia, S.A., Swiersy, A., Radhakrishnan, P., Branchi, V., Kanth Nanduri, L., Gyorffy, B., Betzler, A.M., Bork, U., Kahlert, C., Reissfelder, C., et al. (2016). LDB1 overexpression is a negative prognostic factor in colorectal cancer. Oncotarget *7*, 84258–84270. https://doi.org/10.18632/oncotarget.12481.

86. Li, Y., Zhang, Z., Yu, J., Yin, H., Chu, X., Cao, H., Tao, Y., Zhang, Y., Li, Z., Wu, S., et al. (2024). Enhancer looping protein LDB1 modulates MYB expression in T-ALL cell lines in vitro by cooperating with master transcription factors. J. Exp. Clin. Cancer Res. *43*, 283. https://doi.org/10.1186/s13046-024-03199-1.

87. Gayther, S.A., Batley, S.J., Linger, L., Bannister, A., Thorpe, K., Chin, S.F., Daigo, Y., Russell, P., Wilson, A., Sowter, H.M., et al. (2000). Mutations truncating the EP300 acetylase in human cancers. Nat. Genet. *24*, 300–303. https://doi.org/10.1038/73536.

88. Attar, N., and Kurdistani, S.K. (2017). Exploitation of EP300 and CREBBP Lysine Acetyltransferases by Cancer. Cold Spring Harb. Perspect. Med. *7*, a026534. https://doi.org/10.1101/cshperspect.a026534.

89. Schiano, C., Casamassimi, A., Rienzo, M., de Nigris, F., Sommese, L., and Napoli, C. (2014). Involvement of Mediator complex in malignancy. Biochim. Biophys. Acta *1845*, 66–83. https://doi.org/10.1016/j.bbcan.2013.12.001.

90. Weber, H., and Garabedian, M.J. (2018). The mediator complex in genomic and non-genomic signaling in cancer. Steroids *133*, 8–14. https://doi.org/10.1016/j.steroids.2017.11.007.

91. Rhodes, J.M., Bentley, F.K., Print, C.G., Dorsett, D., Misulovin, Z., Dickinson, E.J., Crosier, K.E., Crosier, P.S., and Horsfield, J.A. (2010). Positive regulation of c-Myc by cohesin is direct, and evolutionarily conserved. Dev. Biol. *344*, 637–649. https://doi.org/10.1016/j.ydbio.2010.05.493.

92. McEwan, M.V., Eccles, M.R., and Horsfield, J.A. (2012). Cohesin is required for activation of MYC by estradiol. PLoS One *7*, e49160. https://doi.org/10.1371/journal.pone.0049160.

93. Schuijers, J., Manteiga, J.C., Weintraub, A.S., Day, D.S., Zamudio, A.V., Hnisz, D., Lee, T.I., and Young, R.A. (2018). Transcriptional Dysregulation of MYC Reveals Common Enhancer-Docking Mechanism. Cell Rep. *23*, 349–360. https://doi.org/10.1016/j.celrep.2018.03.056.

94. Crump, N.T., Ballabio, E., Godfrey, L., Thorne, R., Repapi, E., Kerry, J., Tapia, M., Hua, P., Lagerholm, C., Filippakopoulos, P., et al. (2021). BET inhibition disrupts transcription but retains enhancer-promoter contact. Nat. Commun. *12*, 223. https://doi.org/10.1038/s41467-020-20400-z.

95. Ross, J., Miron, C.E., Plescia, J., Laplante, P., McBride, K., Moitessier, N., and Möröy, T. (2021). Targeting MYC: From understanding its biology to drug discovery. Eur. J. Med. Chem. *213*, 113137. https://doi.org/10.1016/j.ejmech.2020.113137.

96. Gilbert, L.A., Horlbeck, M.A., Adamson, B., Villalta, J.E., Chen, Y., Whitehead, E.H., Guimaraes, C., Panning, B., Ploegh, H.L., Bassik, M.C., et al. (2014). Genome-Scale CRISPR-Mediated Control of Gene Repression and Activation. Cell *159*, 647–661. https://doi.org/10.1016/j.cell.2014.09.029.

97. Krijger, P.H.L., Geeven, G., Bianchi, V., Hilvering, C.R.E., and de Laat, W. (2020). 4C-seq from beginning to end: A detailed protocol for sample preparation and data analysis. Methods *170*, 17–32. https://doi.org/10.1016/j.ymeth.2019.07.014.

98. Love, M.I., Huber, W., and Anders, S. (2014). Moderated estimation of fold change and dispersion for RNA-seq data with DESeq2. Genome Biol. *15*, 550. https://doi.org/10.1186/s13059-014-0550-8.

99. Wang, B., Wang, M., Zhang, W., Xiao, T., Chen, C.H., Wu, A., Wu, F., Traugh, N., Wang, X., Li, Z., et al. (2019). Integrative analysis of pooled CRISPR genetic screens using MAGeCKFlute. Nat. Protoc. *14*, 756–780. https://doi.org/10.1038/s41596-018-0113-7.

100. Kaminow, B., Yunusov, D., and Dobin, A. (2021). STARsolo: accurate, fast and versatile mapping/quantification of single-cell and single-nucleus RNA-seq data. Preprint at bioRxiv. https://doi.org/10.1101/2021.05.05.442755.

101. Li, H., and Durbin, R. (2010). Fast and accurate long-read alignment with Burrows-Wheeler transform. Bioinformatics *26*, 589–595. https://doi.org/10.1093/bioinformatics/btp698.

102. Ramirez, F., Ryan, D.P., Gruning, B., Bhardwaj, V., Kilpert, F., Richter, A.S., Heyne, S., Dundar, F., and Manke, T. (2016). deepTools2: a next generation web server for deep-sequencing data analysis. Nucleic Acids Res. *44*, W160–W165. https://doi.org/10.1093/nar/gkw257.

103. Liao, Y., Smyth, G.K., and Shi, W. (2014). featureCounts: an efficient general purpose program for assigning sequence reads to genomic features. Bioinformatics *30*, 923–930. https://doi.org/10.1093/bioinformatics/btt656.

104. Ewels, P.A., Peltzer, A., Fillinger, S., Patel, H., Alneberg, J., Wilm, A., Garcia, M.U., Di Tommaso, P., and Nahnsen, S. (2020). The nf-core framework for community-curated bioinformatics pipelines. Nat. Biotechnol. *38*, 276–278. https://doi.org/10.1038/s41587-020-0439-x.

105. Di Tommaso, P., Chatzou, M., Floden, E.W., Barja, P.P., Palumbo, E., and Notredame, C. (2017). Nextflow enables reproducible computational workflows. Nat. Biotechnol. *35*, 316–319. https://doi.org/10.1038/nbt.3820.

106. Danecek, P., Bonfield, J.K., Liddle, J., Marshall, J., Ohan, V., Pollard, M.O., Whitwham, A., Keane, T., McCarthy, S.A., Davies, R.M., and Li, H. (2021). Twelve years of SAMtools and BCFtools. GigaScience *10*, giab008. https://doi.org/10.1093/gigascience/giab008.

107. Roberts, T.C., Hart, J.R., Kaikkonen, M.U., Weinberg, M.S., Vogt, P.K., and Morris, K.V. (2015). Quantification of nascent transcription by bromouridine immunocapture nuclear run-on RT-qPCR. Nat. Protoc. *10*, 1198–1211. https://doi.org/10.1038/nprot.2015.076.

108. Hashimshony, T., Senderovich, N., Avital, G., Klochendler, A., de Leeuw, Y., Anavy, L., Gennert, D., Li, S., Livak, K.J., Rozenblatt-Rosen, O., et al. (2016). CEL-Seq2: sensitive highly-multiplexed single-cell RNA-Seq. Genome Biol. *17*, 77. https://doi.org/10.1186/s13059-016-0938-8.

109. Muraro, M.J., Dharmadhikari, G., Grun, D., Groen, N., Dielen, T., Jansen, E., van Gurp, L., Engelse, M.A., Carlotti, F., de Koning, E.J., and van Oudenaarden, A. (2016). A Single-Cell Transcriptome Atlas of the Human Pancreas. Cell Syst *3*, 385–394. https://doi.org/10.1016/j.cels.2016.09.002.

110. Gruning, B., Dale, R., Sjodin, A., Chapman, B.A., Rowe, J., Tomkins-Tinch, C.H., Valieris, R., Koster, J., and Bioconda, T. (2018). Bioconda: sustainable and comprehensive software distribution for the life sciences. Nat. Methods *15*, 475–476. https://doi.org/10.1038/s41592-018-0046-7.

111. Dekker, J., Belmont, A.S., Guttman, M., Leshyk, V.O., Lis, J.T., Lomvardas, S., Mirny, L.A., O'Shea, C.C., Park, P.J., Ren, B., et al. (2017). The 4D nucleome project. Nature *549*, 219–226. https://doi.org/10.1038/nature23884.

STAR★METHODS

KEY RESOURCES TABLE

REAGENT or RESOURCE	SOURCE	IDENTIFIER
Antibodies		
Mouse monoclonal anti-RAD21	Merck	Cat#05–908; RRID:AB_417383
Mouse monoclonal anti-GAPDH	Santa Cruz	Cat#sc-32233; RRID:AB_627679
Mouse monoclonal anti-FLAG	Merck	Cat#F1804; RRID:AB_262044
Mouse monoclonal anti-SF3B1	Santa Cruz	Cat#sc-514655; RRID:AB_2941964
Goat monoclonal anti-Mouse IgG (H + L), HRP Conjugate	Promega	Cat#W4021; RRID:AB_430834
Mouse monoclonal anti-BrdU	BD Pharmingen	Cat#555627; RRID:AB_395993
Bacterial and virus strains		
ElectroMAX Stbl4 Competent Cells	Invitrogen	Cat#11635018
One Shot TOP10 Chemically Competent E. coli	Invitrogen	Cat#C404010
Chemicals, peptides, and recombinant proteins		
RPMI 1640	Gibco	Cat#72400021
FBS	Sigma-Aldrich	Cat#F7524
Pen-strep	Gibco	Cat#15070063
Tryple	Gibco	Cat#12605010
Puromycin	Merck	Cat#P9620
Blasticidin	Sigma-Aldrich	Cat#15205
PEI	Polysciences	Cat#23966
Polybrene	Sigma-Aldrich	Cat#H9268-5G
5-Ph-IAA	MedChemExpress	Cat#HY-134653
dTAG7	TOCRIS	Cat#6912
5-Bromouridine	Sigma-Aldrich	Cat#850187
BlpI	ThermoFisher	Cat#ER0091
BstXI	ThermoFisher	Cat#ER1021
Csp6I	ThermoFisher	Cat#R0125
NlaIII	NEB	Cat#ER0211
T4 DNA Ligase	Roche	Cat#10799009001
Proteinase K	Roche	Cat#3115801001
TRIzol	Invitrogen	Cat#15596026
RNAseOUT	Invitrogen	Cat#10777019
Critical commercial assays		
NEBNext Ultra II Directional RNA Library Prep Kit	NEB	Cat#E7760S
NEBNext Poly(A) mRNA Magnetic Isolation Module	NEB	Cat#E7490S
Q5 High-Fidelity DNA Polymerase	NEB	Cat# M0491S
P-beads	Macherey Nagel	Cat#744100.34
Expand Long Template PCR system	Roche	Cat#11759060001
AMPure-XP beads	Beckman Coulter	Cat#A63881
Protein G Dynabeads	ThermoFisher	Cat#10004D
SuperSignal West Pico PLUS Chemiluminescent Substrate	ThermoFisher	Cat#34580

(*Continued on next page*)

Continued		
REAGENT or RESOURCE	SOURCE	IDENTIFIER
4–15% Mini-PROTEAN TGX Precast Protein Gel	Biorad	Cat#4561083
Deposited data		
Raw western blot images	This study	Mendeley Data; https://doi.org/10.17632/r9878ks8nm.1
Reporter CRISPRi screen	This study	GEO; GSE281575
RAD21-AID_BrU-seq	This study	GEO; GSE281753
MED23-FKBP_BrU-seq	This study	GEO; GSE281753
LDB1-FKBP_BrU-seq	This study	GEO; GSE281753
CRISPRi_KD_RNAseq_highthroughput	This study	GEO; GSE281752
CRISPRi_KD_RNAseq_total	This study	GEO; GSE280672
4C-seq	This study	GEO; GSE281419; GSE281420; GSE281576
ENCODE-rE2G predictions, K562	Gschwind et al.[10]; ENCODE	ENCFF202FID
MED14-AID_SLAMseq	Bell et al.[48]	GEO; GSE198944
JQ1_SLAMseq	Muhar et al.[53]	GEO; GSE100708
LDB1_ChIPseq	Guo et al.[49]	GEO; GSE142228
GENCODE Human reference genome GRCh38/hg38	GENCODE	https://ftp.ebi.ac.uk/pub/databases/gencode/Gencode_human/release_44/GRCh38.primary_assembly.genome.fa.gz
GENCODE Comprehensive gene annotation V44	GENCODE	https://ftp.ebi.ac.uk/pub/databases/gencode/Gencode_human/release_44/gencode.v44.annotation.gtf.gz
UCSC Human reference genome GRCh38/hg38	UCSC	http://hgdownload.cse.ucsc.edu/goldenpath/hg38
UCSC Human reference genome GRCh37/hg19	UCSC	http://hgdownload.cse.ucsc.edu/goldenpath/hg19
Experimental models: Cell lines		
K562_E0-GFP_dCas9-KRAB-BFP	Rinzema et al.[32]	N/A
K562_E-50-GFP_dCas9-KRAB-BFP	Rinzema et al.[32]	N/A
K562_E100-GFP_dCas9-KRAB-BFP	Rinzema et al.[32]	N/A
K562_EC100-GFP_dCas9-KRAB-BFP	Rinzema et al.[32]	N/A
K562_EC100-GFP_RAD21-AID; LV-Tir1	This study	N/A
K562_EC100-GFP_MED23-FKBP	This study	N/A
K562_EC100-GFP_LDB1-FKBP	This study	N/A
Oligonucleotides		
sgRNA library for CRISPRi screen	Table S1	N/A
Other oligonucleotides	Table S3	N/A
Recombinant DNA		
pU6-sgRNA EF1Alpha-puro-T2A-BFP	Gilbert et al.[96]	Addgene #60955
pHR-SFFV-dCas9-BFP-KRAB	Gilbert et al.[96]	Addgene #46911
pMK393 (mAID-mCherry-BSD)	Yesbolatova et al.[43]	Addgene #121194
pMK381 (AAVS1 CMV-OsTIR1F74G)	Yesbolatova et al.[43]	Addgene #140536
pSpCas9(BB)-2A-BFP	This study	N/A
RAD21-AID targeting construct	This study	N/A
pLV_Tir1 construct	This study	N/A
MED23-FKBP targeting construct	This study	N/A
LDB1-FKBP targeting construct	This study	N/A

(*Continued on next page*)

Continued

REAGENT or RESOURCE	SOURCE	IDENTIFIER
**Software and algorithms**		
CytExpert v2.3.1.22	Beckman Coulter	N/A
Code to analyze sequencing data	This study	Zenodo, https://doi.org/10.5281/zenodo.14731771
4DN ChIP-seq pipeline	4DN	https://github.com/4dn-dcic/chip-seq-pipeline2
pipe4C	Krijger et al.[97]	https://github.com/deLaatLab/pipe4C
DESeq2	Love et al.[98]	https://doi.org/10.18129/B9.bioc.DESeq2
Mageck MLE	Wang et al.[99]	https://sourceforge.net/projects/mageck/
STARsolo (v2.7.11a)	Kaminow et al.[100]	https://github.com/alexdobin/STAR
BWA (v0.7.17-r1188)	Li et al.[101]	https://github.com/lh3/bwa
deeptools (v3.5.4)	Ramirez et al.[102]	https://github.com/deeptools/deepTools
featurecounts (v2.0.6)	Liao et al.[103]	https://subread.sourceforge.net/
nf-core v3.14.0	Ewels et al.[104]	https://nf-co.re/
nextflow v23.10.1	Di Tommaso et al.[105]	https://www.nextflow.io/
salmon (1.10.1)	https://github.com/COMBINE-lab/salmon/	https://github.com/COMBINE-lab/salmon/
Samtools (v1.18)	Danecek et al.[106]	https://github.com/samtools/samtools
**Other**		
FACSAria Fusion Flow Cytometer	BD	N/A
Cytoflex S Flow Cytometer	Beckman Coulter	N/A
Amaxa Nucleofector II	Lonza	N/A
ImageQuant 800 imager	Amersham	N/A

EXPERIMENTAL MODEL AND STUDY PARTICIPANT DETAILS

Human erythroleukemia K562 cells (female) were cultured in RPMI 1640 (Gibco) supplemented with 10% FBS (Sigma-Aldrich) and 1% pen–strep (Gibco). Cells were grown at a maximal density of 5x10ˆ5 cells/ml. For all experiments where reporter GFP level was interrogated, experiments were performed maximum 1 week after FACS sorting cells GFP positive for 2 consecutive weeks with gating based on non-GFP expressing K562 cells. HEK293T cells were cultured in the same growth medium as K562 cells, and split using Tryple (Gibco). Cells were regularly tested for mycoplasma and split or refreshed every 2–3 days. Cell lines were not specifically authenticated.

METHOD DETAILS

CRISPRi sgRNA library cloning

The library was cloned in expression vectors as described before (Gilbert 2014). In short: oligos containing the sgRNA sequences were synthesized by Twist Bioscience (sequences in table S1). Oligos were amplified with primers 5′-ATTTTGCCCCTGGTTCTT-3′ and 5′-CCCTAAGAAATGAACTGG-3′, digested with BstXI and BlpI, purified and ligated into digested lentiviral targeting plasmid pU6-sgRNA EF1Alpha-puro-T2A-BFP (Addgene plasmid #60955). The library was then transformed to ElectroMAX Stbl4 Competent Cells (Thermo) and grown on bioassay dishes whilst assuring sufficient coverage of the library. After library isolation, the library was sequenced to confirm that its complexity was sufficient. sgRNA-library virus was produced by PEI transfection (Polysciences) of HEK293T cells using standard packaging vectors. Virus containing supernatant was harvested two days after transfection. Supernatant was filtered using a 0.45μm filter, snap frozen in liquid nitrogen and stored at −80 until use.

CRISPRi screen

dCas9-KRAB was randomly integrated into E0, E−50, E100, EC100 reporter cell lines via lentiviral transduction of a modified version of pHR-SFFV-dCas9-BFP-KRAB (Addgene plasmid #46911). dCas9-BFP-KRAB cells were bulk sorted for BFP integration and continuously cultured on blasticidin (0.7 μg/ml, Sigma-Aldrich, Cat#15205). For library CRISPRi screening, dCas9-BFP-KRAB, HBG-GFP reporter cells were transduced with sgRNA library virus at low MOI overnight with 8 μg/ml polybrene (Merck), and the next day refreshed in medium containing blasticidin. One day later, cells were selected using 1 μg/ml Puromycin (Sigma-Aldrich, Cat#P9620) and refreshed daily. After 5 days of selection, cells were sorted on a BD FACSAria Fusion Flow Cytometer. Live cells were gated for sgRNA-BFP with a threshold based on untransduced cells of the corresponding reporter containing

dCas9-KRAB-BFP. From this population, cells were sorted for the highest and lowest 10% GFP level for that specific reporter line. From these GFP high and GFP low populations, DNA was isolated using phenol chloroform extraction, followed by isopropanol precipitation. guideRNA sequences were amplified using Q5 PCR (NEB, Cat#M0491L) with primers containing overhangs for Illumina sequencing (see table S3).

Single-guide CRISPRi knock down

For targeted knock down of factors identified in the CRISPRi screens, per factor one sgRNA was cloned into the sgRNA-BFP backbone (pU6-sgRNA EF1Alpha-puro-T2A-BFP) using TOP10 cells (sequences in table S3). sgRNAs were individually transduced and selected in dCas9-KRAB expressing reporter cell lines in the same manner as the pooled screen protocol and as described in.[32] Cells were measured on Beckman Coulter Cytoflex S. Gating on live cells and BFP/GFP was performed in the same manner as for the pooled screens. GFP levels were quantified in the BFP++ population (expressing both dCas9-KRAB-BFP as well as the sgRNA) with gating based on non-transduced control cells (expressing only dCas9-KRAB-BFP). Median GFP levels were compared to the average of two non-targeting sgRNAs after subtraction of GFP intensity in a reporter-GFP cell line that did not contain an enhancer. Values were normalized for the effect of the HBG-promoter targeting sgRNA.

mAID tagging of RAD21

Fragments containing the mAID-mCherry and PGK-BSD sequences were PCR amplified from plasmid pMK393 (Addgene #121194) and cloned into a vector containing homology arms for the RAD21 C terminus. Cloning was performed using In-Fusion HD Cloning Kit (Takara Bio) and plasmids were verified with sanger sequencing. Targeting plasmids were transfected into the EC100 cell line together with pSpCas9(BB)-2A-BFP (modified version of PX458 (Addgene #48138)) containing an sgRNA targeting RAD21 C terminus. Transfections were performed using a Lonza Amaxa Nucleofector II with setting Program T-03. After transfection, cells were selected using 0.7 μg/mL blasticidin, and mCherry-positive cells were single-cell sorted into 96-well plates. Clonal lines were characterized using PCR and sanger sequencing to ensure correct homozygous tagging. For integration of OsTIR1, the OsTIRF74G sequence was PCR amplified from pMK381 (Addgene #140536), fused with a T2A-Puro sequence and cloned into a lentiviral backbone based on Addgene plasmid #46911. This construct was transduced into two RAD21-mAID clones, and these cells were selected with puromycin until untransduced control cells were all dead.

FKBP tagging of MED23 and LDB1

To target MED23 and LDB1 we used a 3xFLAG-FKBP_F36V tag (gift from Jop Kind). MED23 was targeted with an N-terminal construct of mCherry-P2A-FKBP, and LDB1 was tagged with C-terminal 3xFLAG-FKBP_F36V-P2A-mCherry-P2A-BlastR. As for RAD21, these constructs were transfected into EC100 with a corresponding sgRNA. For LDB1 targeting, cells were blasticidin selected. Cells were clonally sorted on high mCherry levels, genotyped using PCR, and sanger sequenced. FLAG western blot was performed to confirm correct target protein-tag fusion size.

Protein depletion

To perform acute RAD21 depletion, cells were incubated with 1μM 5-Ph-IAA (MedChemExpress, Cat#HY-134653). Depletion was confirmed using flow cytometry (for RAD21-mCherry) and western blot for endogenous RAD21 protein. To deplete MED23 and LDB1, dTAG-7 (TOCRIS, Cat#6912) was added to the medium in a final concentration of 500nM. Depletion was monitored using western blot for FLAG tag, and flow cytometry based measurement of reporter GFP.

Western blot

For detection of protein levels, equal amounts of cells were pelleted and directly resuspended in Laemmli buffer. After boiling at 95° for 10 min, samples were loaded on a 4–15% Mini-PROTEAN TGX Precast Protein Gel (Biorad Cat#4561083). Proteins were transferred to a PVDF membrane, which was blocked with 5% milk in PBS-T (0.1% Tween 20 in PBS), and incubated overnight at 4° with indicated antibodies. Antibodies used were: FLAG (F1804, Merck, 1:1000), RAD21 (05–908, Merck, 1:1000), GAPDH (sc-32233, Santa Cruz, 1:1000), SF3B1 (sc-514655, Santa Cruz, 1:100). After washing with PBS-T, secondary antibody anti-mouse HRP conjugate (W4021, Promega, 1:5000) was added to the blot for 1 h at room temperature. Blots were developed using SuperSignal West Pico PLUS Chemiluminescent Substrate (ThermoFisher) and visualized with a ImageQuant 800 imager (Amersham).

Reporter flow cytometry after acute depletion

To monitor EC100 reporter response to protein depletion, degron cell lines were treated with either 1μM 5-Ph-IAA (RAD21), 500nM dTAG7 (MED23 and LDB1), or DMSO as control. GFP expression was measured on Beckman Coulter Cytoflex S after 4H, 8H, 12H or 24H of depletion. Values are plotted normalized to cells treated with DMSO for the same time. To exclude the effect of ligand on reporter levels, 5-Ph-IAA or dTAG7 was added to parental EC100 cell line and GFP was measured after 24H and compared to DMSO.

4C-seq

4C-seq was performed as described.[97] In short, 5-10M cells were crosslinked with 2% formaldehyde for 10 min and quenched with 0.125M final concentration glycine on ice. Pellets were resuspended in lysis buffer (50 mM Tris-HCl pH 7.5, 0.5% NP-40, 1% Triton

X-100, 150 mM NaCl, 5 mM EDTA) for 20 min on ice. Then, SDS (0.3% final concentration) was added, followed by Triton X-100 (2.5% final concentration), and chromatin was digested *in situ* using 100U Csp6I (ThermoFisher Cat#ER0211). DNA was then ligated using T4 ligase (Roche Cat#10799009001), decrosslinked by overnight ProtK treatment (Roche Cat#3115801001), and purified using isopropanol precipitation. Samples were then digested using 50U NlaIII (NEB Cat#R0125), ligated at a concentration of 5ng template/ul, and final 4C templates were purified with P-Beads (Macherey Nagel Cat#744100.34). 4C PCRs were performed in a two-step manner using the Expand Long Template PCR system (Roche Cat#11759060001). Specific 4C primers were first used to amplify a viewpoint of interest (table S3), followed by a second round of PCR using primers containing Illumina sequencing adapters.[97]

Nascent RNA-sequencing (BrU-seq)

After indicated treatment of cells, nascent RNA was labeled by addition of BrU (5-Bromouridine, Sigma-Aldrich, #850187) to K562 cell culture medium in 2mM final concentration for 6 min. Cells were then immediately put on ice, centrifuged for 4 min at 200G at 4° and resuspended in TRIzol (Invitrogen). RNA was isolated using conventional TRIzol extraction and isopropanol precipitation, and final pellet was resuspended in DEPC. 50ug of total RNA was used for BrU pulldown, following a protocol adapted from Roberts 2015.[107] In short, per sample 6ug of Anti-BrdU antibody (BD Pharmingen, Cat#555627) was incubated with 30ul of Protein G Dynabeads (ThermoFisher) for 10 min at room temperature, and washed in PBS-T. Beads were then blocked with 0.05 μg/μl BSA in PBS-T for 30 min and washed with PBS-TR (PBS-T supplemented with RNAseOUT (ThermoFisher)). RNA was diluted in PBS-TR and heated at 65° for 5 min, after which it was added to antibody-coated beads at room temperature for 1 h in 300ul total volume. Beads were then washed three times in PBS-TR, and BrU-labeled enriched RNA was extracted using TRIzol and isopropanol precipitation. Pellets were resuspended in 5ul DEPC, which was used as input for NEBNext Ultra II Directional RNA Library Prep Kit (NEB, cat. no. E7645).

Poly(A) RNA-sequencing

For knockdown of target factors MED23, MED16, MED12 and CCNC, followed by RNA-seq, CRISPRi knockdowns were performed as described above. At sample harvest, 1 million live cells were sorted BFP-high (sgRNA-containing) for RNA isolation. NEBNext Poly(A) mRNA Magnetic Isolation Module (NEB, cat. no E7490) was used to enrich for polyA mRNA. Sequencing libraries were then produced using NEBNext Ultra II Directional RNA Library.

High-throughput bulk RNA-sequencing

For knockdown of other factors, CRISPRi knockdowns were performed as described above. High-throughput bulk RNA-seq plates were prepared by adding 1 μL of 7.5 ng/μL CEL-seq2 primers[108] to 96-well plates. These primers contain sample-specific barcodes that were used to barcode and amplify the mRNA from each sample. After combination of BFP-sorted cell suspension with the sample barcode, a mixture of dNTP's and synthetic ERCC spike in molecules were added to the plates using a liquid handling platform and cells were lysed at 65°C. Then, a Reverse Transcription mix with Superscript II enzyme was added to barcode and reverse transcribe the mRNA from all samples to cDNA. After incubation at 42°C, second strand synthesis was done by adding a mixture of DNA polymerase, ligase and RNAseH enzyme, after which material from all samples was pooled and purified. Next, material from all samples was frozen and stored, and part of it was amplified using *In Vitro* Transcription. The resulting amplified RNA was prepared into sequencing ready libraries following the SORT-seq library preparation protocol.[109] Sequencing libraries were indexed with Illumina Truseq small RNA primers.

Analysis of CRISPRi screen

Reads containing sgRNA sequences were trimmed and mapped to the sgRNA sequences in the custom library (table S1) as described in https://github.com/deLaatLab/Tjalsma_2025. For further analysis, Mageck MLE[99] was run using 624 negative control (non-targeting) sgRNAs present in the library for normalization. A cell line transduced with the library not sorted for GFP was set as baseline. Per TSS, a beta score was calculated per gate. The effect on each reporter was calculated by subtracting the beta scores of the GFP-high from the GFP-low population. These scores were normalized by dividing by the log2FC of the HBG reporter TSS sgRNA (HBG2_+_5275954.23-P1P2_5) in the specific clone and visualized in scatterplots using ggplot2. Results are in table S2.

Analysis of 4C-seq

4C-seq reads were mapped to the UCSC hg38 reference genome and processed using pipe4C (https://github.com/deLaatLab/pipe4C). For 4C profiles from the HBG-GFP reporter locus, reads were mapped against the UCSC hg19 reference genome modified with integrated reporter sequences (as described in[32]). Normalized 4C coverage was calculated using R (r-project.org). Counts at non-blind fragments were adjusted to 1 million intra-chromosomal mapped reads after exclusion of the two highest-count fragments. Count data was smoothed using a running mean with a window size of 21 fragments using the R package caTools v1.18.2.

Nascent RNA-sequencing (BrU-seq) analysis

Raw reads were aligned to the hg38 reference genome (GENCODE primary assembly GRCh38), modified to include the d2EGFP_SV40polyA sequence as separate chromosome, using BWA (v0.7.17-r1188) and filtered with a minimum mapping quality (MQ) of 15 using Samtools (v1.18).

Bigwig files were created with deeptools (v3.5.4) with the following parameters: –ignoreForNormalization chrM, –binSize 10, –minMappingQuality 15, –normalizeUsing RPGC, and –effectiveGenomeSize 2913022398.

Read counts were generated with featureCounts (v2.0.6) using the GENCODE comprehensive gene annotation (release V44 CHR) as the annotation reference. Read counts were filtered for minimal 5 counts in the DMSO samples and normalized using DeSeq2 (1.38.3) with DMSO as the reference condition. For analysis of *HBB* genes (*HBG1, HBG2, HBE1*) in BrU-seq data, only reads mapping to introns were used for quantification.

Poly(A) RNA-sequencing analysis

The raw sequencing data were processed using nf-core/rnaseq v3.14.0 of the nf-core collection of workflows,[104] utilizing reproducible software environments from the Bioconda[110] project. The pipeline was executed with Nextflow v23.10.1[103]. Sequencing reads were aligned to the hg38 reference genome (Gencode primary assembly GRCh38) and GENCODE comprehensive gene annotation (V44) using STAR (2.7.10a). Post-alignment, gene-level quantification was performed using Salmon (1.10.1). Gene counts were normalized using the median ratio method in DESeq2 and log2FC values were calculated with gRNA_Control as reference.

High-throughput bulk RNA-sequencing analysis

Raw reads were used as input for STARsolo (v2.7.11a) with parameters –soloType CB_UMI_Simple, –soloCBstart 7, –soloCBlen 8, –soloUMIstart 1, –soloUMIlen 6, –soloBarcodeReadLength 0, –soloStrand Forward, –soloCBmatchWLtype 1MM_multi, –soloUMIdedup NoDedup, –soloMultiMappers Unique EM, –soloUMIfiltering -. As reference the GENCODE primary assembly GRCh38 and the GENCODE comprehensive gene annotation (release V44 CHR) were used, excluding globin transcripts ENST00000642908.1, ENST00000647543.1, ENST00000648735.1, ENST00000380252.6, ENST00000646569.1, ENST00000643199.1, ENST00000380259.7, ENST00000292896.3, ENST00000380237.5, ENST00000380315.2, ENST00000475226.1, ENST00000643122.1, ENST00000429817.1. Raw counts of the two replicates with the lowest counts were collapsed, resulting in 2 replicates per factor. Next counts were normalized using DeSeq2 1.38.3 with gRNA_Control_T as reference.

E2G-based analysis

ENCODE-rE2G (E2G) predictions for K562 cells were downloaded from ENCODE (thresholded element gene links, ENCFF202FID). RNA-sequencing data at gene level was added to the E2G table using Ensembl ID. We considered genes as regulated by enhancers using the following conditions. No enhancer: no link between the TSS and any element above 2kb that has an E2G score $\geq$0.3. Short-range: at least one link to an element with a distance between 2 and 10kb with an E2G score $\geq$0.8, and no link with any element above 10kB with an E2G score $\geq$0.3. Mid-range: at least one link to an element with a distance between 10 and 40kB with an E2G score $\geq$0.8, and no link with any element above 40kB with an E2G score $\geq$0.3. Long-range: at least one link to an element with a distance between 50kb and 500kb with an E2G score $\geq$0.8. Genes that did not fall into one of these groups were not included in the E2G analyses. For the BrU-seq and total RNA-seq data, we excluded genes with a baseMean lower than 50. For the MED14 SLAM-seq data, we excluded genes with a baseMean lower than 8. When intersecting MED14 and RAD21 datasets, the gene set from the MED14 data was used.

For further comparison between enhancer-connected groups, the log2 fold change values resulting from DEseq2 were used. When multiple elements were connected to a gene within the same group (for example, 2 enhancers located more than 50kb away from the TSS with both an E2G score above 0.8), the enhancer with the highest E2G score was used for further analysis. For the CCD analysis after RAD21 depletion, if any of the enhancer-promoter links above the cutoffs was present within a CCD, the gene was considered in the category ‘inside CCD’. For comparing features across the designated enhancers, the enhancers linked to the genes as defined in the short, mid, and long groups were ranked on the specific feature in the E2G table. Genes were then separated as connected to either the lowest or highest 25% that feature at their linked enhancers and their log2FCs were compared.

For integration with LDB1 ChIP-seq data, this was performed in the same manner but genes were ranked on average LDB1 coverage on their linked enhancer and then separated as connected to either the lowest or highest 25% LDB1-levels.

Published SLAM-seq data

For MED14 SLAM-seq data[48] transcript counts were downloaded from GEO(GSE198944), aggregated to gene level (GENCODE V44) and normalized with DEseq2. For JQ1 SLAM-seq data,[53] DEseq2 outputs were downloaded from GEO (GSE100708). DEseq2 log2-FoldChange for each gene was then merged with the E2G table using Ensembl ID and used for comparison between enhancer-connected categories.

ChIP-seq analysis

ChIP-seq reads for LDB1[49] were mapped to the hg38 reference genome and processed using the 4DN ChIP-seq pipeline (https://github.com/4dn-dcic/chip-seq-pipeline2) from the 4D Nucleome Network (4DN),[111] which uses the ENCODE ChIP-seq pipeline v2.1.6 (https://github.com/ENCODE-DCC/chip-seq-pipeline2). As a measure for relative coverage, the average fold change for every unique enhancer region in the E2G dataset was calculated from the fold change BigWigs based on two replicates.

QUANTIFICATION AND STATISTICAL ANALYSIS

Statistical analyses performed and sample sizes are described in the figure legends and methods section. DEseq2 normalized counts were used to calculate log2FC and FDR adjusted Wald test for the endogenous *HBB* genes. For the E2G-based analysis, DEseq2 log2FCs of the different enhancer-regulated groups as defined above were compared. R (version 4.3.2) was used to calculate two-sided Wilcoxon rank-sum test or one-sided Komolgorov-Smirnov test values as indicated. When subdividing enhancer groups based on features, the number of genes per group is depicted above the individual plots or in the figure legends. Statistical significance was defined as follows: ns (not significant) = $p > 0.05$, * = $p < 0.05$, ** = $p < 0.01$, *** = $p < 0.001$. Boxplots represent the 25th percentile, the median, and 75th percentile, and the whiskers indicate 1.5 times the interquartile range (IQR), with dots showing values outside this range.

Cell Genomics, Volume *5*

Supplemental information

Long-range enhancer-controlled genes are hypersensitive to regulatory factor perturbations

Sjoerd J.D. Tjalsma, Niels J. Rinzema, Marjon J.A.M. Verstegen, Michelle J. Robers, Andrea Nieto-Aliseda, Richard A. Gremmen, Amin Allahyar, Mauro J. Muraro, Peter H.L. Krijger, and Wouter de Laat

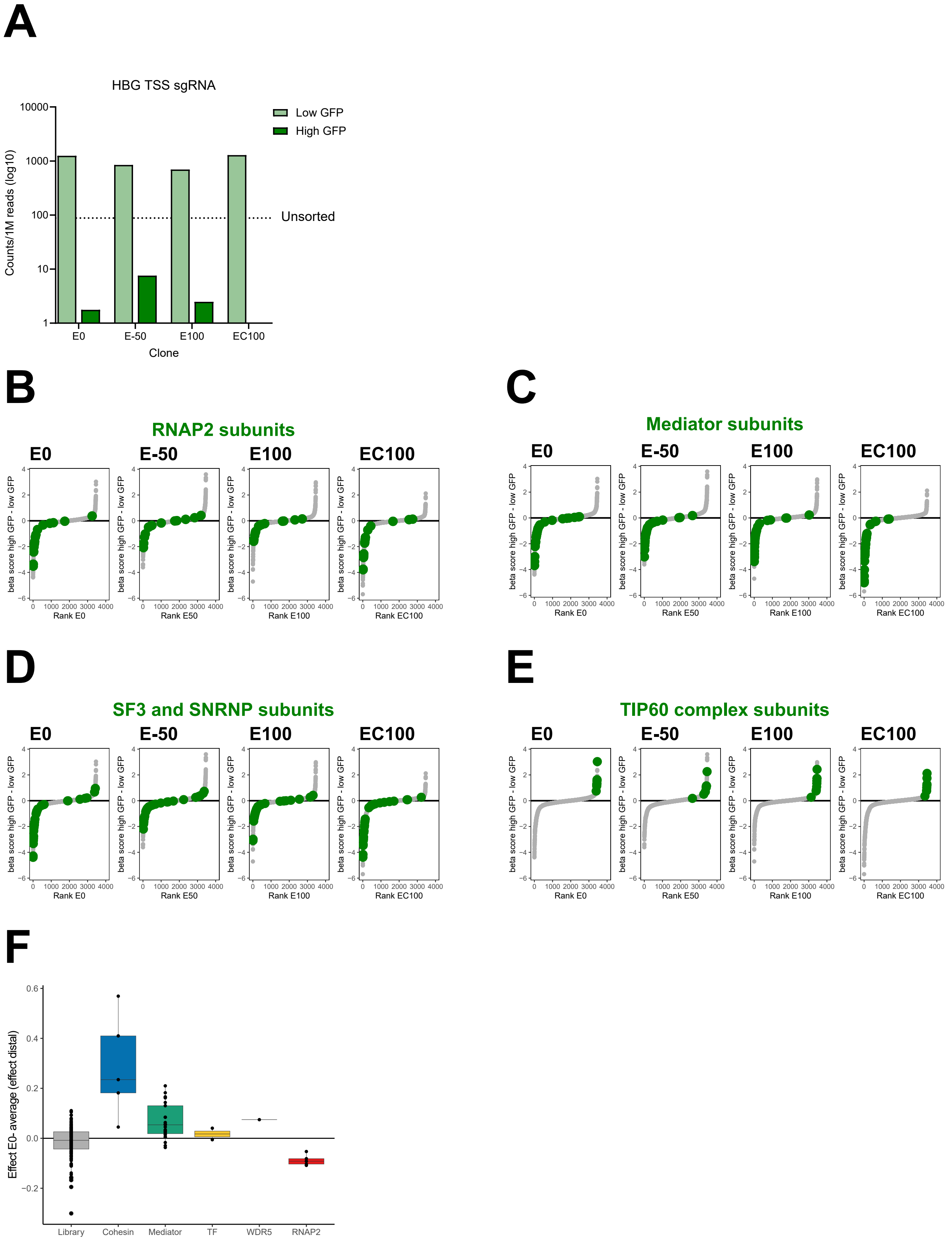

Figure S1. Reporter CRISPRi screen, related to Figure 1. (A) Read count of sgRNA directly targeting the HBG-reporter promoter, in the low GFP (light green) or high GFP (dark green) conditions, normalized for sequencing depth. The dotted line indicates the counts in the library isolated for unsorted cells. **(B-E)** Distribution of subunits of RNAP2 **(B)**, mediator **(C)**, the SF3 or SNRNP complexes **(D)**, and the TIP60 complex **(E)** in the CRISPRi screens per reporter cell line. (**F)** Box plots comparing the average distal enrichment score with the E0 score for factors affecting distal reporter expression. Factors with a normalized score of ≤ -0.1 in at least one of the distal gates were compared to E0 and ranked on the difference with the average of the three distal reporters. Each complex highlighted in figure 1 is plotted as a separate box plot.

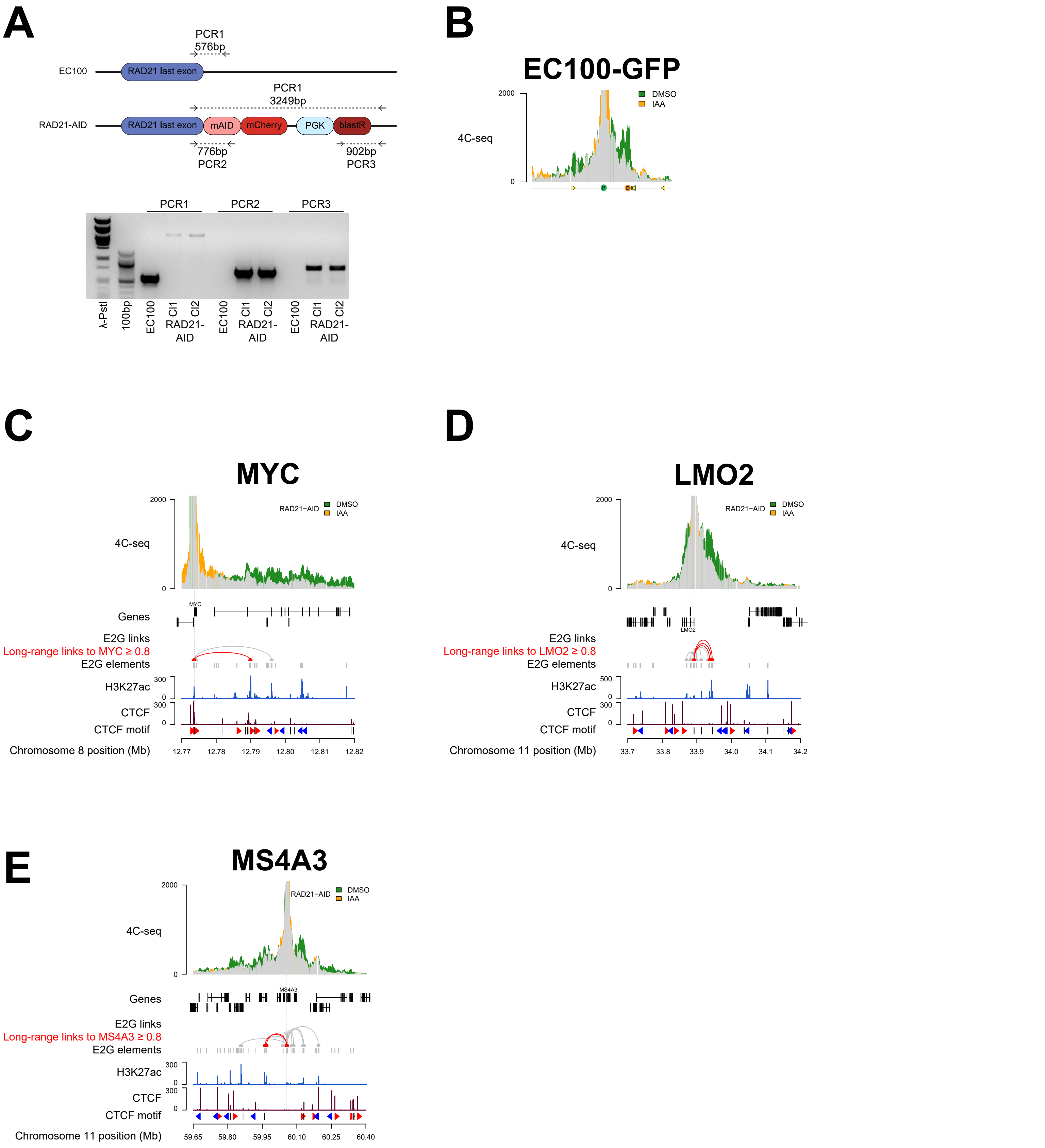

Figure S2. Generation and validation of RAD21-AID cell lines, related to Figure 2. (A) Genotyping PCRs of RAD21-AID clones. EC100 = parental cell line (untargeted). **(B-E)** 4C-seq profiles of EC100-GFP reporter cells, treated for 2 hours with DMSO or 5-Ph-IAA (IAA). The average profile of two clones is plotted as overlay of DMSO vs IAA. In green, interactions specific for DMSO samples, in yellow interactions specific for IAA samples, in grey shared interactions. Viewpoints: **(B)** EC100-GFP reporter locus. **(C)** MYC. **(D)** LMO2. **(E)** MS4A3. For **(B)** yellow triangles denote CTCF sites. E: enhancer. P: reporter promoter.

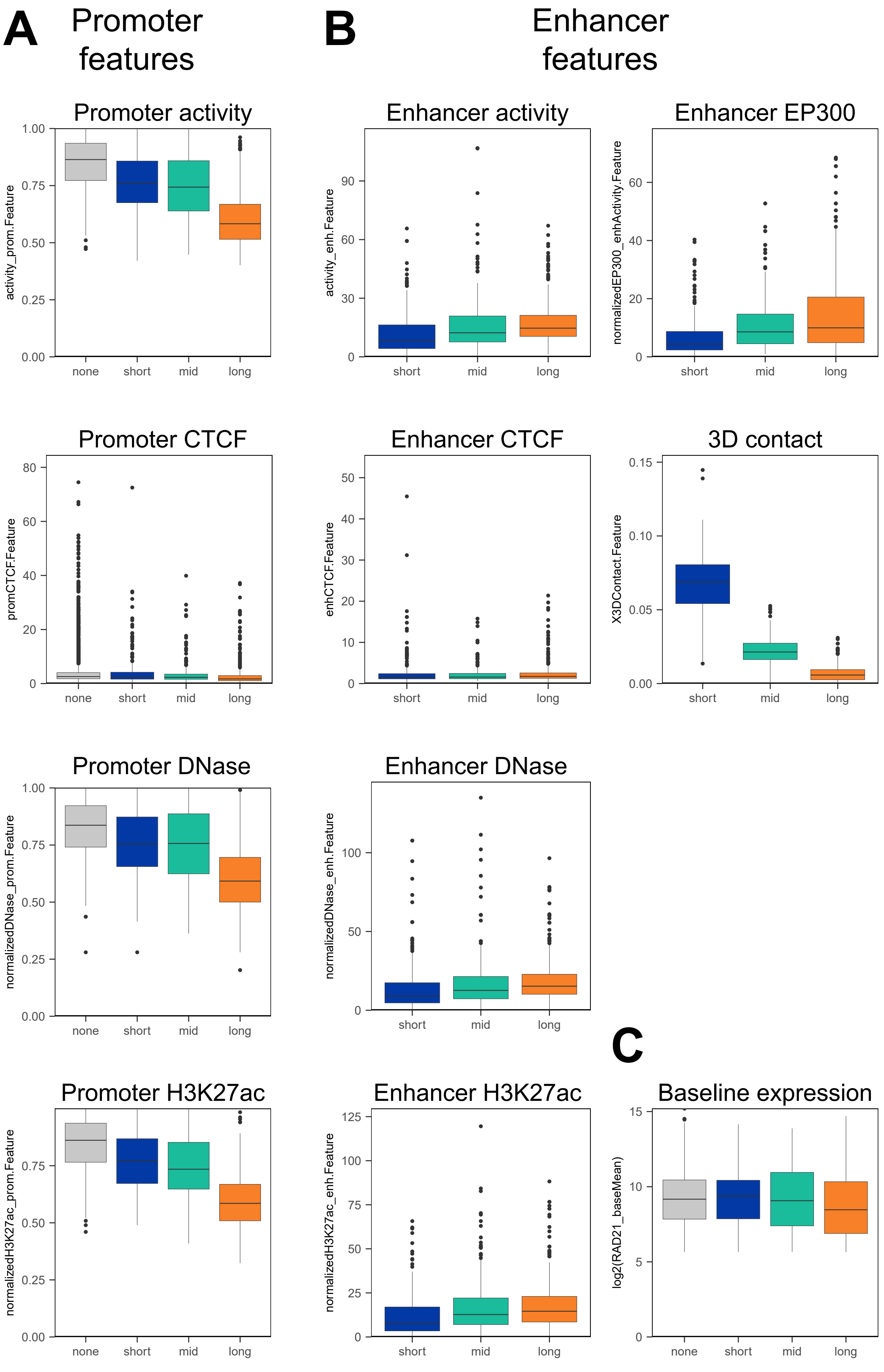

Figure S3. Characterization of enhancer-contacting genes, related to Figure 2. Features of promoters **(A)** and enhancers **(B)** in the ENCODE-rE2G datasets. For genes that contacted multiple enhancers within the same group (eg. 2 long-range enhancers both with an ABC score ≥ 0.8), the enhancer with the highest ABC score was picked for these analyses. **(C)** Baseline counts of enhancer categorized genes (baseMean counts from DEseq2).

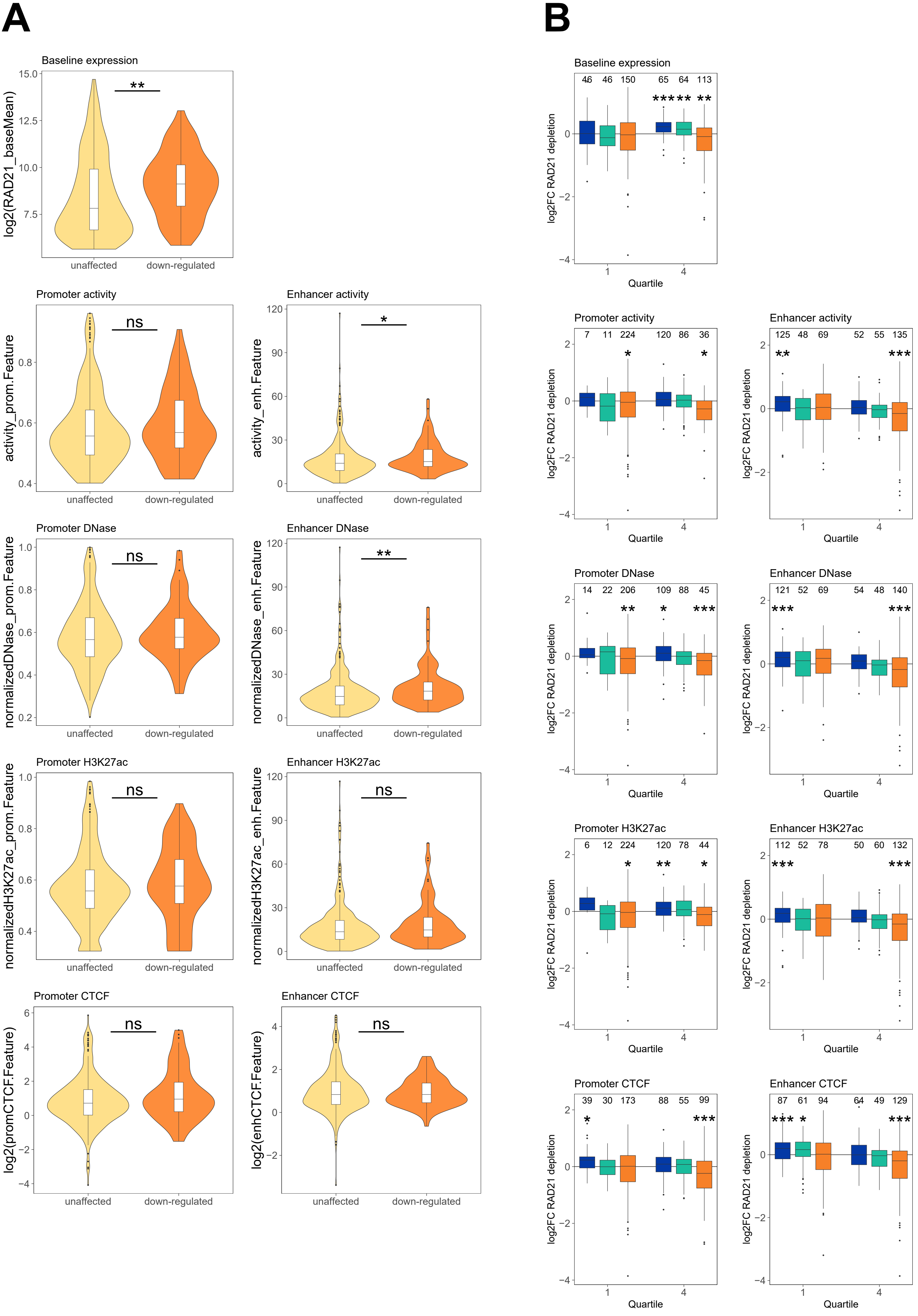
A
B
Baseline expression
log2(RAD21_baseMean)
**
unaffected
down-regulated
Promoter activity
activity_prom.Feature
ns
Enhancer activity
activity_enh.Feature
*
Promoter DNase
normalizedDNase_prom.Feature
Enhancer DNase
normalizedDNase_enh.Feature
Promoter H3K27ac
normalizedH3K27ac_prom.Feature
Enhancer H3K27ac
normalizedH3K27ac_enh.Feature
Promoter CTCF
log2(promCTCF.Feature)
Enhancer CTCF
log2(enhCTCF.Feature)
log2FC RAD21 depletion
Quartile
46 46 150 65 64 113
7 11 224 120 86 36
125 48 69 52 55 135
14 22 206 109 88 45
121 52 69 54 48 140
6 12 224 120 78 44
112 52 78 50 60 132
39 30 173 88 55 99
87 61 94 64 49 129

Figure S4. Features of RAD21 responsive genes, related to Figure 2. (A) Comparison of enhancer features of long-range enhancers found down-regulated (log2FC < -0.8, FDR < 0.05) or unaffected (-0.2 < log2FC < 0.2) by RAD21 depletion. n=332 for unaffected, n= 72 for down-regulated. **(B)** Comparison of response to RAD21 depletion for genes connected to enhancers with specific features. For every feature, the strongest enhancer per gene in the short (blue), mid (green), and long orange) categories were pooled and ranked on the feature. Then, the lowest 25% (quartile 1) and the highest 25% (quartile 4) of these enhancers were selected, and the response of their connected genes to RAD21 depletion was depicted as log2FC. p-values calculated with 2-sided Wilcoxon rank-sum test of the specific group versus the no enhancer genes. ns=non-significant, *=p<0.05, **=p<0.01, ***=p<0.001.

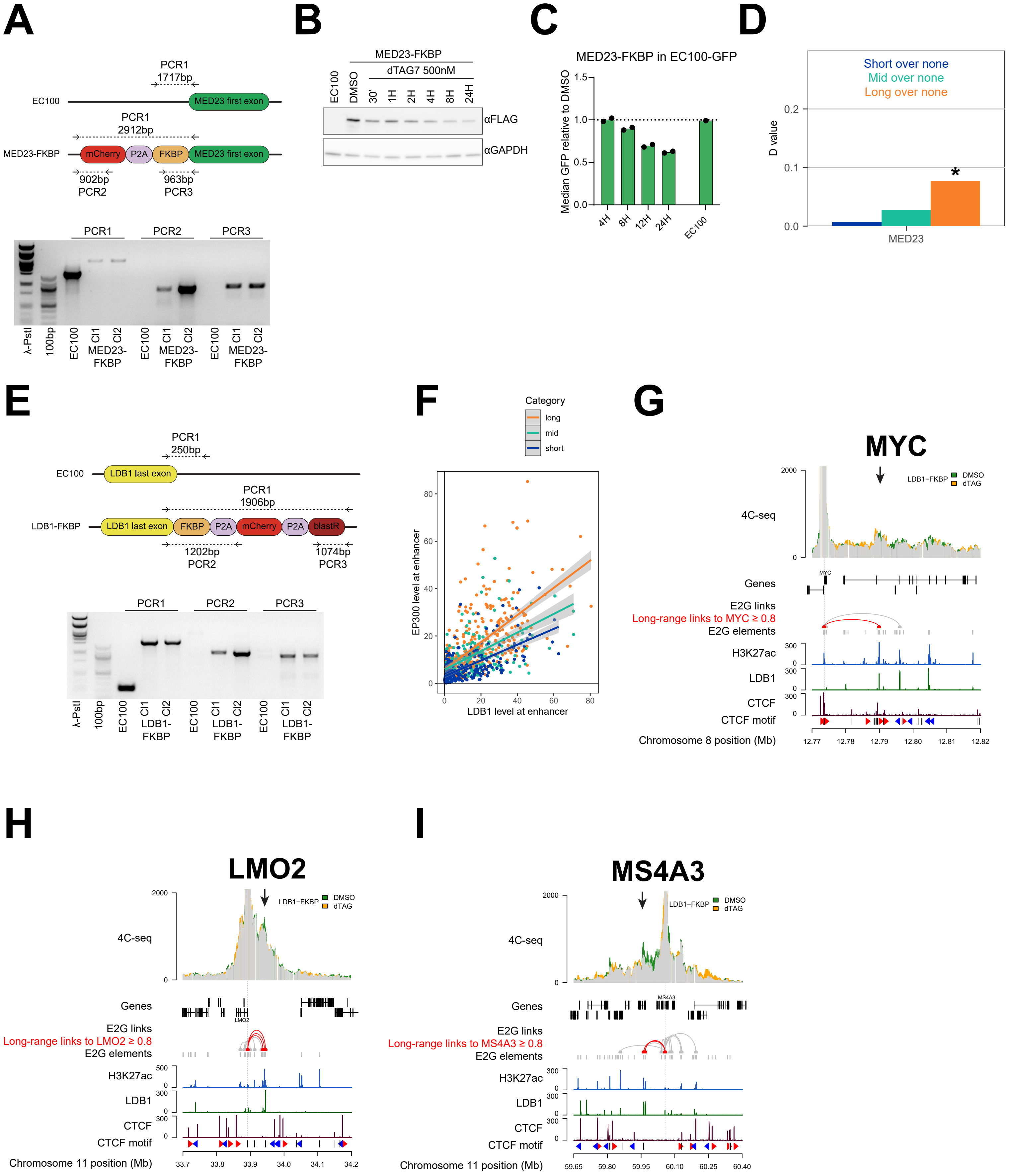

Figure S5. Generation and validation of MED23 and LDB1 degron cell lines, related to Figure 3. (A) Genotyping PCRs of FKBP-MED23 clonal cell lines. **(B)** Western blot for FLAG-tagged FKBP-MED23, fter treatment with 500nM dTAG7 for indicated time points, one representative FKBP-MED23 clone. **(C)** FACS validation of GFP reporter after MED23 depletion. Each dot represents one FKBP-MED23 clone. **(D)** D values of one-sided Komolgorov-Smirnov test, for three enhancer categories over no enhancer genes, *=p<0.05. n=2 cell lines. **(E)** Genotyping PCRs of LDB1-FKBP clonal cell lines. **(F)** Scatter plot comparing linked E2G enhancers in EP300 level (y-axis) versus LDB1 level (x-axis). **(G-I)** 4C-seq profiles of LDB1-FKBP cells, treated for 4 hours with DMSO or dTAG7 (dTAG). The average profile of two clones is plotted as overlay of DMSO vs dTAG. In green, interactions specific for DMSO samples, in yellow interactions specific for dTAG samples, in grey shared interactions. Viewpoints: **(G)** MYC, **(H)** LMO2, **(I)** MS4A3. Arrow denotes strong enhancer with LDB1 binding.

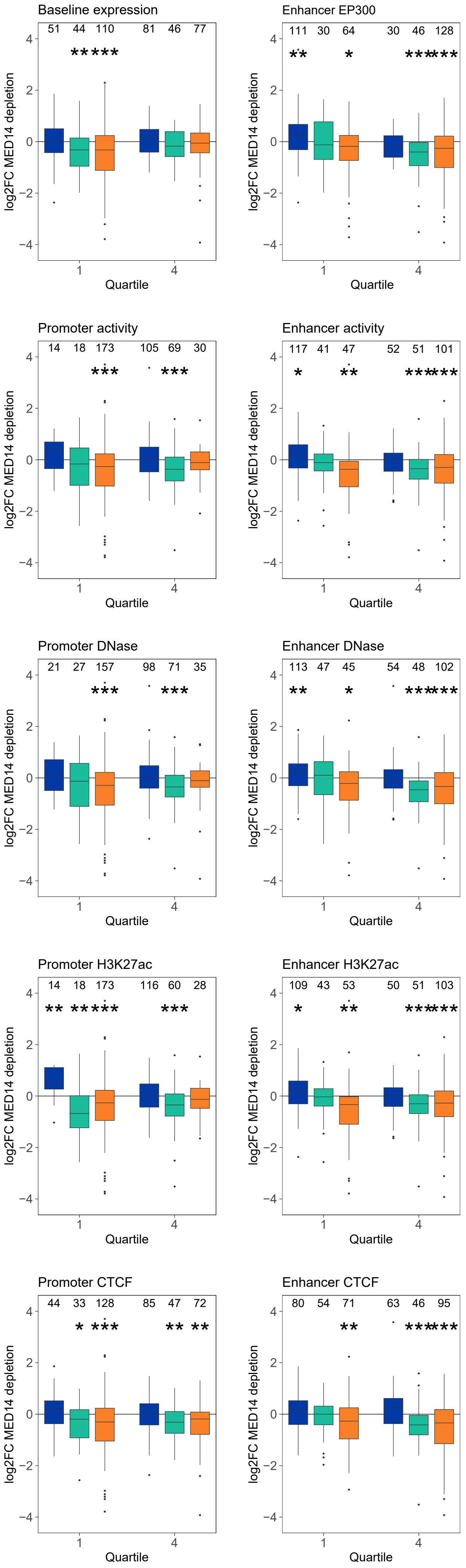

Figure S6. Features of MED14 responsive genes, related to Figure 3. Comparison of response to MED14 depletion for genes connected to enhancers with specific features. For every feature, the strongest enhancer per gene in the short, mid, and long categories were pooled and ranked on the feature. Then, the lowest 25% (quartile 1) and the highest 25% (quartile 4) of these enhancers were selected, and the response of their connected genes to MED14 depletion was depicted as log2FC. p-values calculated with 2-sided Wilcoxon rank-sum test of the specific group versus the no enhancer genes. ns=non-significant, *=p<0.05, **=p<0.01, ***=p<0.001. Only for visualization purposes, genes with a log2FC outside the (-4,4) range were not plotted.

Figure S7

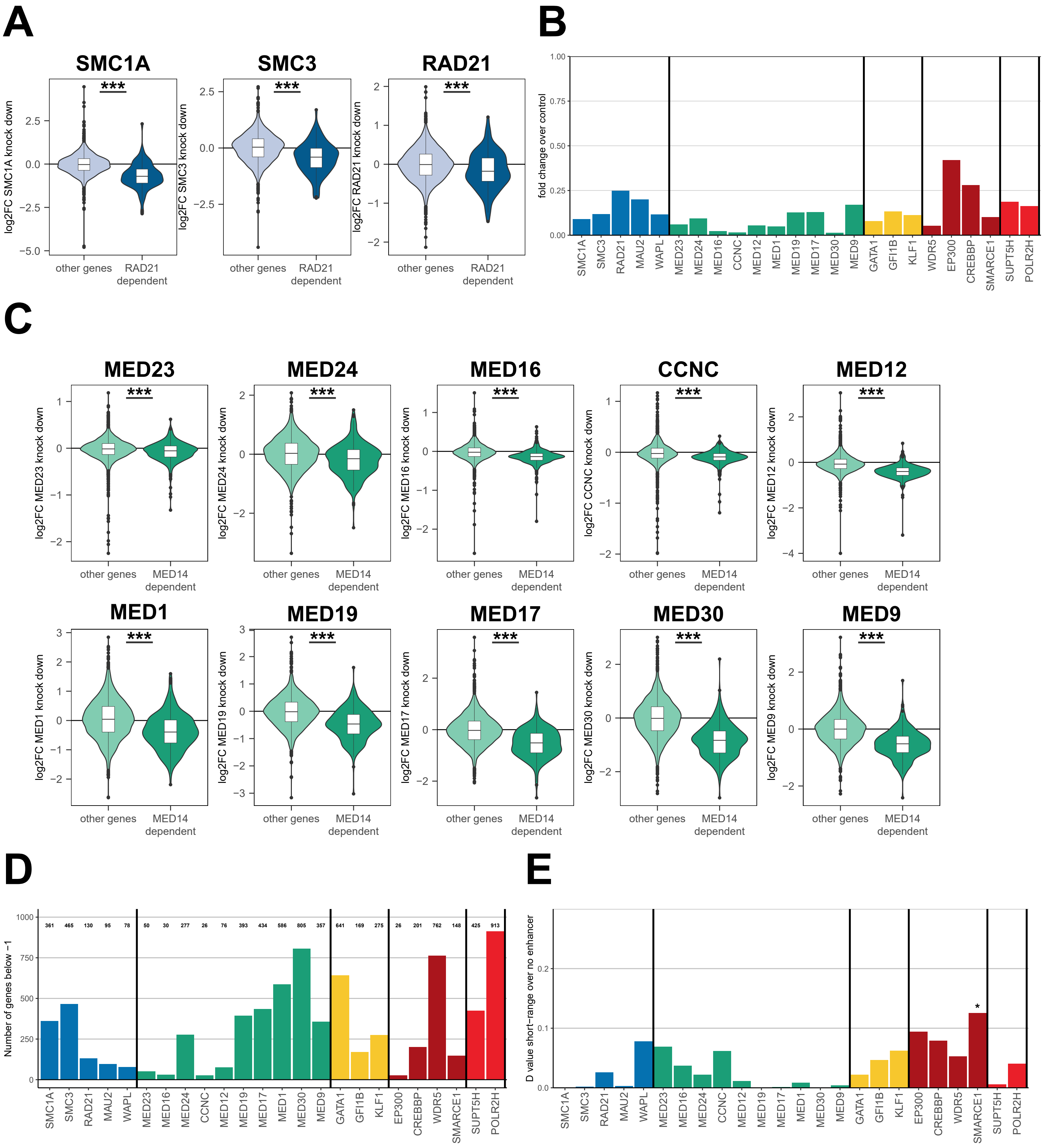

Figure S7. CRISPRi knock down and RNA-seq of regulatory factors, related to Figure 4. (A) log2 fold change of genes downregulated after acute RAD21 depletion (RAD21 dependent, log2FC <-0.8, FDR<0.05), compared to all other genes (RAD21 independent), after knock down of SMC1A, SMC3 or RAD21. 2-sided Wilcoxon rank-sum test, ***=p<0.001. **(B)** Fold change of target factors after knock down, compared to a non-targeting control sgRNA. **(C)** log2 fold change of genes downregulated after acute MED14 depletion (MED14 dependent, log2FC <-0.8, FDR<0.05), compared to all other, after knock down of subunits of the mediator complex. 2-sided Wilcoxon rank-sum test, ***=p<0.001. **(D)** Number of genes downregulated after target knock down, log2 fold change < -0.8. **(E)** Komolgorov-Smirnov D values of gene sets after knock down of target factors, short-range genes compared to no enhancer genes. One-sided test, *=p<0.05.

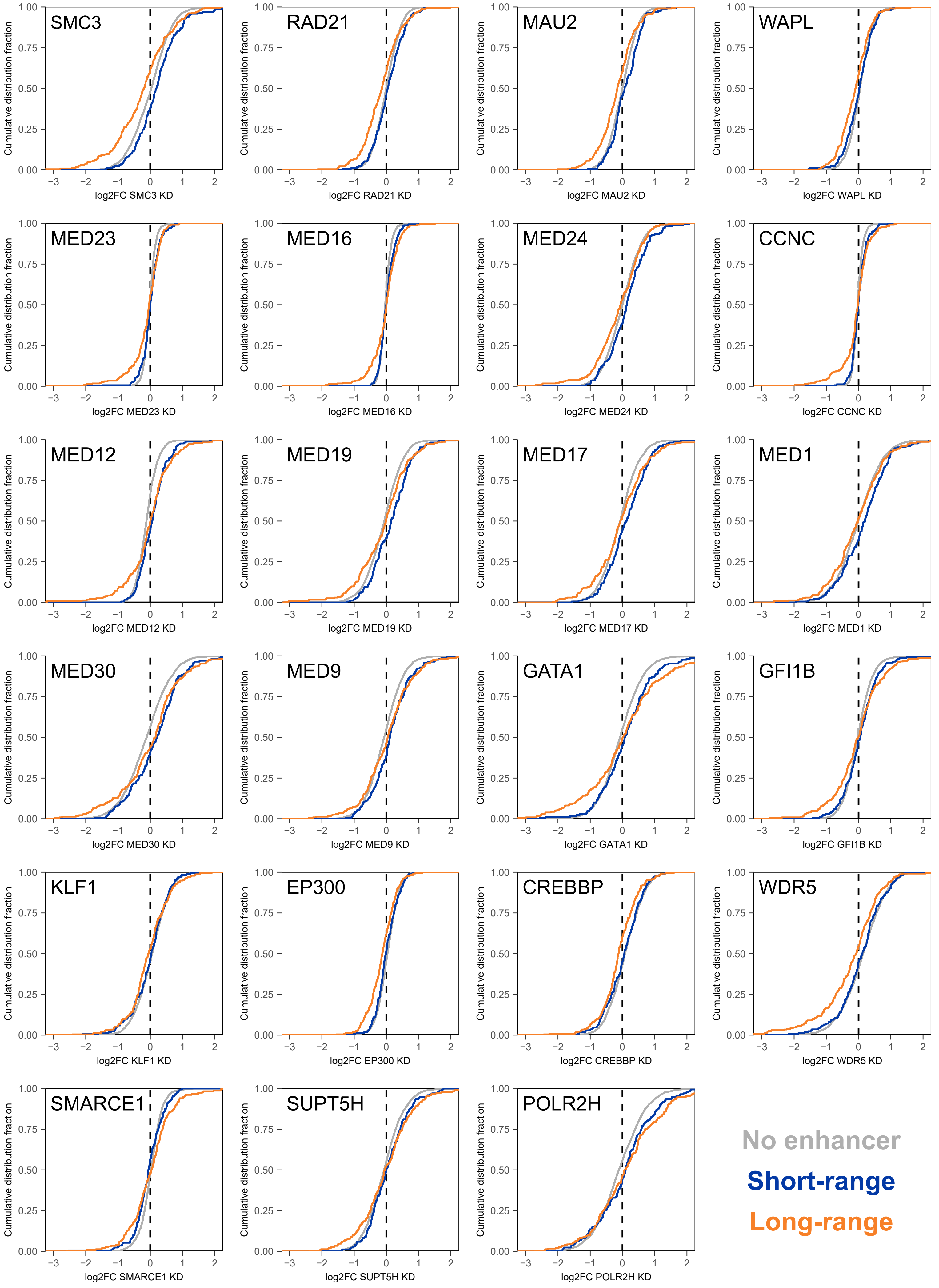

Figure S8. Cumulative distribution curves of log2 fold changes of enhancer-categorized genes after knock down of factors, related to Figure 4.

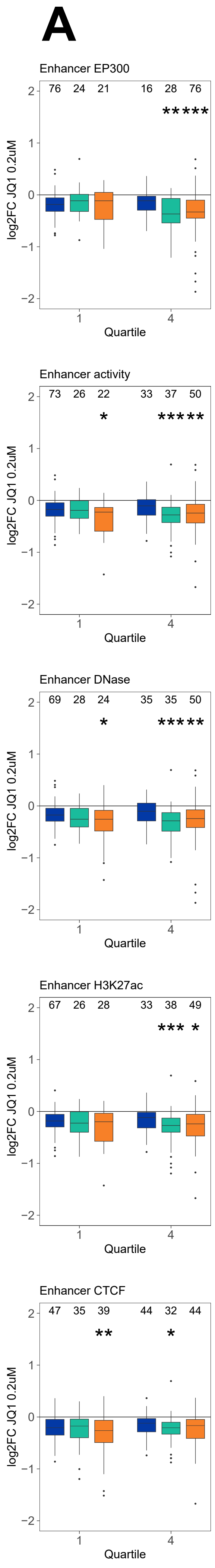

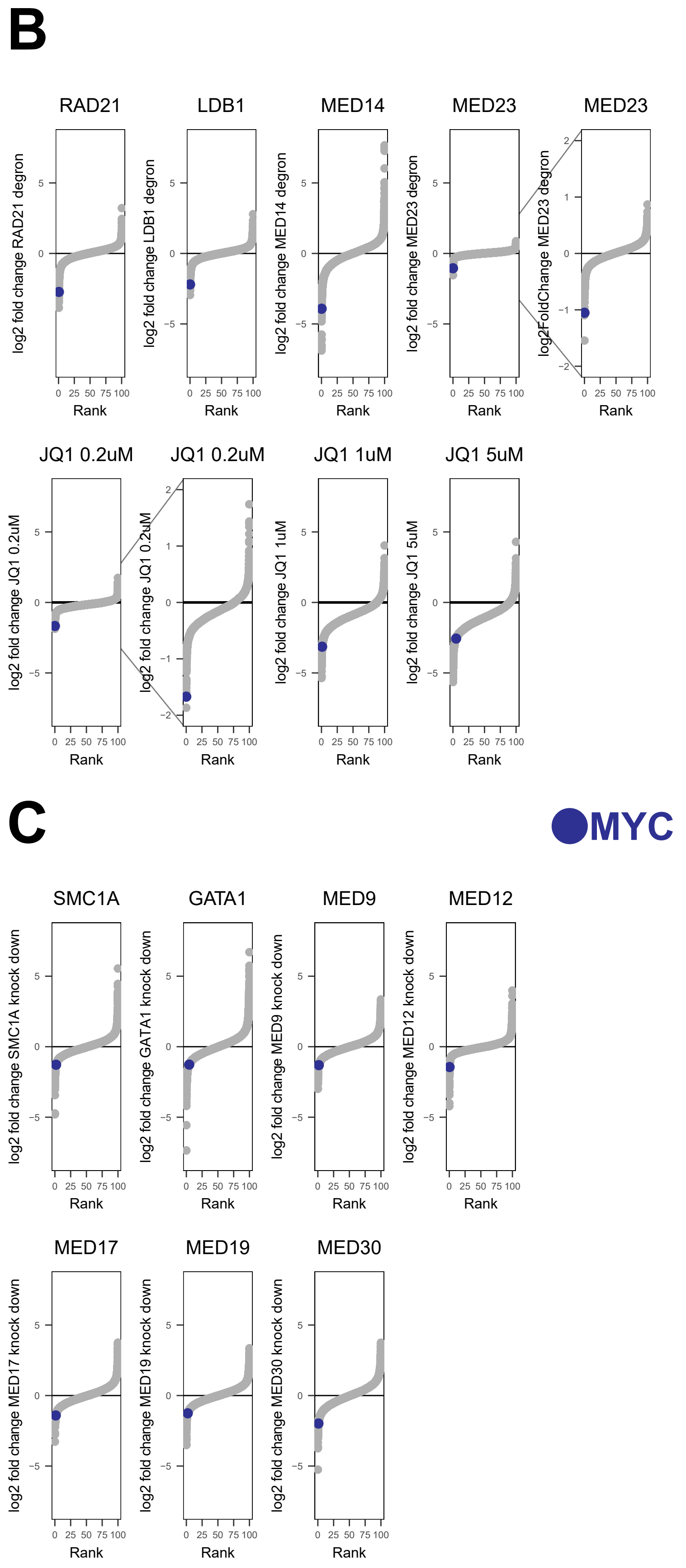

Figure S9. Features of JQ1 responsive genes, and MYC, related to Figure 4. (A) Comparison of response to JQ1 treatment (200nM) for genes connected to enhancers with specific features. For every feature, the strongest enhancer per gene in the short, mid, and long categories were pooled and ranked on the feature. Then, the lowest 25% (quartile 1) and the highest 25% (quartile 4) of these enhancers were selected, and the response of their connected genes to JQ1 treatment was depicted as log2FC. p-values calculated with 2-sided Wilcoxon rank-sum test of the specific group versus the no enhancer genes. ns=non-significant, *=p<0.05, **=p<0.01, ***=p<0.001. **(B)** All genes in nascent RNA-seq after acute factor perturbations or JQ1 treatment, or **(C)** all genes in total RNA-seq after CRISPRi factor knock down, were ranked on log2 fold change, and MYC was plotted. Ranks were plotted as a percentage of the total number of genes in the data set.
